# A revision of *Peronospora* species on *Veronica* unravels a species-rich group of downy mildew pathogens with host shifts to economically important ornamental plants

**DOI:** 10.3897/imafungus.17.186696

**Published:** 2026-03-23

**Authors:** Man Mu, Young-Joon Choi, Thomas Brand, Sebastian Ploch, Hermann Voglmayr, Marco Thines

**Affiliations:** 1 Institute of Ecology, Evolution and Diversity, Department of Biological Sciences, Goethe University, Max-von-Laue-Str. 13, 60439 Frankfurt am Main, Germany Chamber of Agriculture Lower Saxony, Plant Protection Service Oldenburg Germany; 2 Senckenberg Biodiversity and Climate Research Centre, Senckenberganlage 25, 60325 Frankfurt am Main, Germany Senckenberg Biodiversity and Climate Research Centre Frankfurt am Main Germany; 3 Department of Biological Science, College of Ocean, Natural Sciences, and Engineering, Kunsan National University, 54150 Gunsan, Republic of Korea College of Ocean, Natural Sciences, and Engineering, Kunsan National University Gunsan Republic of Korea; 4 Chamber of Agriculture Lower Saxony, Plant Protection Service, Sedanstr. 4, 26121 Oldenburg, Germany Department of Botany and Biodiversity Research, University of Vienna Wien Austria; 5 Department of Botany and Biodiversity Research, University of Vienna, Rennweg 14, 1030 Wien, Austria Department of Biological Sciences, Goethe University Frankfurt am Main Germany

**Keywords:** Evolution, new taxa, *
Oomycota
*, *
Peronosporaceae
*, phylogeny, *
Plantaginaceae
*

## Abstract

*Peronospora* is the largest genus of the *Oomycota*, responsible for causing downy mildew on a wide range of cultivated and ornamental plants worldwide. Although more than 400 *Peronospora* species have been described, many host-pathogen relationships have not been thoroughly explored, particularly in relation to the phylogenetic connections of pathogens from type hosts and additional hosts reported for the various described species. At the same time, infections of *Peronospora* on economically important hosts are an emerging threat, often with uncertainty regarding the causal agents. In this study, as an example for obligate biotrophic pathogens, 105 specimens of *Peronospora* parasitising species of the genus *Veronica* and one specimen parasitising the related host genus *Paederota* (both *Plantaginaceae*), were analysed for their morphology and phylogenetic relationships using multi-locus reconstructions. As a result, nine new *Peronospora* species parasitising *Veronica* were identified and are described in this manuscript, while the morphology of seven previously described *Peronospora* species was re-examined. While generally, a high degree of host specialisation was found, *Peronospora verna* and *P. grisea* were found to be indifferentiable, suggesting a recent host shift, and *P. silvestris* was found to also infect *Globularia nudicaulis*. Likewise it was found that infections on the ornamental subgenus *Hebe* caused by *P. palustris* and *P. petricosa* are the result of host shifts of European species onto non-native hosts. The presence of *Peronospora* on nearly 500 *Veronica* species that are not type hosts for any described *Peronospora* species should be re-examined, as these occurrences likely include many previously overlooked species with unknown pathogenicity.

## Introduction

Although more than 400 species of *Peronospora* have been described so far (Constantinescu 1991; Thines and Choi 2016), many of these species were lumped into classifications based on a broad species concept, primarily relying on the host plant family for identification. Thus, many *Peronospora* species have been reported on multiple host species, despite recent studies demonstrating that most *Peronospora* species exhibit high host specificity. Therefore, it is necessary to re-examine host-pathogen relationships across different hosts. Additionally, many species that have been phylogenetically characterised were misidentified due to conflicting reports of host associations or the absence of type host specimens to confirm species identification on additional hosts. In addition to this, new *Peronospora* records have been emerging on economically important hosts (Thines and Choi 2016), for some of which it could be confirmed that they are the result of host shifts to hosts previously naïve to the pathogens, e.g. the new occurrence of *Peronospora belbahrii* on *Lavandula angustifolia* (Thines et al. 2020), or *Peronospora aquilegiicola* on cultivated columbines (Thines et al. 2019). Similarly, *Veronica* species of the economically important subgenus *Hebe* have been reported as hosts to *Peronospora* in Europe (Polizzi et al. 2004; Thines and Choi 2016) and other parts of the world (Jafar 1962; Cunnington 2008), yet their identity could not be clarified so far.

*Veronica* is a widely distributed genus of herbaceous to shrubby plants and includes medicinal herbs and ornamental plants distributed over several countries. So far, nine *Peronospora* species infecting *Veronica* have been validly published (Table 1).

**Table 1.** *Peronospora* species described so far and their reported hosts.

	Reported hosts	Type host	Known geographic range	Notes	Citations
*Peronospora agrestis*	*Veronica agrestis*, *V. arvensis*, *V. chamaedrys*, *V. opaca*, *V. persica*, *V. polita*	*V. polita*	Europe, Asia	Commonly reported in Europe	Gäumann (1918, 1923); Stevenson (1926); Notizen (1934); Dennis (1986); Yu (1998); Voglmayr (2003)
*Peronospora aquatica*	*V. anagallis-aquatica* (syn. *Veronica anagallis* Cham. & Schltdl.), *V. undulata*	*V. anagallis-aquatica*	Europe, Asia, North America, Taiwan	Commonly reported, includes a Taiwan record on V. undulata	Gäumann (1918, 1923); Yu (1998); Göker et al. (2003), 2007; PDI Taiwan (https://mycolab.pp.nchu.edu.tw/PDI_Taiwan/)
*Peronospora arvensis*	*V. hederifolia*, *V. sublobata*, *V. triloba*, *V. triphyllos*	*V. hederifolia*	Europe	Commonly reported in Europe	Gäumann (1918, 1923); Stevenson (1926); Voglmayr (2003); Klenke and Scholler (2015); Ellis (2025)
*Peronospora grisea*	*V. arvensis*, *V. beccabunga*, *V. hederifolia*, *V. peregrina*, *V. serpyllifolia*, *V. verna*	*V. beccabunga*	Europe, North America	Reported on both aquatic and ruderal hosts	Unger (1847); de Bary (1863); Swingle (1887); Gäumann (1923); Stevenson (1926); Riethmüller et al. (2002); Voglmayr (2003)
*Peronospora palustris*	*V. scutellata*	*V. scutellata*	Europe	Recorded from wetland *Veronica*	Gäumann (1918, 1923); Stevenson (1926)
*Peronospora saxatilis*	*V. fruticans*	*V. fruticans*	Switzerland	Reported from alpine habitats	Gäumann (1918, 1923)
*Peronospora silvestris*	*V. officinalis*, *V. urticifolia*	*V. officinalis*	Europe	Reported from forest-dwelling and ruderal hosts	Gäumann (1918, 1923); Riethmüller et al. (2002)
*Peronospora verna*	*V. arvensis*, *V. chamaedrys*, *V. praecox*, *V. prostrata*, *V. serpyllifolia*, *V. speciosa*, *V. teucrium*, *V. tournefortii*, *V. verna*	*V. serpyllifolia*	Europe, Asia, North America	Commonly reported on various hosts in the northern hemisphere	Gäumann (1918, 1923)
*Peronospora veronicae-cymbalariae*	*V. cymbalaria*	*V. cymbalaria*	Palestine	So far known only from the type locality	Rayss (1945)

Since these reports, *Peronospora* species on *Veronica* have rarely been documented. Considering the many *Veronica* species that are reportedly infected by downy mildews (Klenke and Scholler 2015), some of which are very frequent (Thines and Kruse 2017), and the high degree of host specificity observed in *Peronospora* (Choi et al. 2008, 2015b; García-Blázquez et al. 2008; Voglmayr et al. 2014; Hoffmeister et al. 2020; Ploch et al. 2022; Mu et al. 2024a, b, c), it is likely that several *Peronospora* species parasitising *Veronica* have been previously overlooked. At the same time, it remains unclear if the downy mildew of *Veronica* subgenus *Hebe* is caused by pathogens that can be related to known species of *Peronospora* or if previously overlooked species are the causal agents.

*Peronospora* on *Veronica* likely includes many undescribed species, considering that about 500 species of *Veronica* have been reported as hosts for downy mildew, yet only nine *Peronospora* species were validly described as parasites of the genus. Since *Veronica* species are also widely used as ornamental plants, understanding the diversity and host specialisation of downy mildew species on these plants is also critical for devising phytosanitary measures and the prevention of plant diseases. Thus, to disentangle relationships of *Peronospora* on *Veronica*, multigene analyses, in addition to detailed morphological examinations, were done in the current study.

## Materials and methods

### Plant and oomycete material

In total, 105 *Peronospora* specimens originating from *Veronica* and one specimen from *Paederota* were examined from several countries and regions in Eurasia, Oceania, and South America, including specimens from Argentina, Austria, Australia, the Czech Republic, Germany, Greece, Palestine, South Korea, Spain, Sweden, and Switzerland.

The samples were either collected by the authors or loaned from the following herbaria (acronyms according to Index Herbariorum https://sweetgum.nybg.org/science/ih/) DAR, GLM, KUS-F, WU, and ZT, or received from private collections J.K., collection of Julia Kruse, Germany; M., collection of Marco Thines, Germany; R.D., Robert Delhey in the Phytopathology Lab of Bahía Blanca, Argentina; T.B., collection of Thomas Brand, Germany; Details of the specimens used in this study are provided in Table 2.

**Table 2.** Information on the specimens and sequences used in this study.

Species	Herb. No.	Host	Year	Geographic origin	GenBank accession number										Reference
					ITS	LSU D1-3	LSU D6-8	*COX*1	*COX*2	NAD1	*RPS*10	ITS (host)	*matK* (host)	*trnL-F* (host)	
*Peronospora agrestis*	GLM-F068541	*Veronica polita*	1984	Germany	PV999305	PV999406	PV999507	PX025038	PX025144	PX025249	PX025352	PX021816	PX024958	PX024954	This study
*Peronospora agrestis*	WU-MYC0032792	*Veronica polita*	2004	Austria	PV999306	PV999407	PV999508	PX025039	PX025145	PX025250	PX025353	PX021817	PX024959	—	This study
*Peronospora agrestis*	GLM-F073381	*Veronica persica*	2003	Germany	PV999307	PV999408	PV999509	PX025040	PX025146	PX025251	PX025354	PX021818	—	—	This study
*Peronospora agrestis*	GLM-F074038	*Veronica persica*	1998	Germany	PV999308	PV999409	PV999510	PX025041	PX025147	PX025252	PX025355	PX021819	—	—	This study
*Peronospora agrestis*	GLM-F078101	*Veronica persica*	2002	Germany	PV999309	PV999410	PV999511	PX025042	PX025148	PX025253	PX025356	PX021820	—	—	This study
*Peronospora agrestis*	M.600	*Veronica persica*	—	—	PV999310	PV999411	PV999512	PX025043	PX025149	PX025254	PX025357	PX021821	—	—	This study
*Peronospora agrestis*	GLM-F068565	*Veronica filiformis*	1984	Germany	PV999311	PV999412	PV999513	PX025044	PX025150	PX025255	PX025358	PX021822	—	—	This study
*Peronospora agrestis*	J.K.0460	*Veronica filiformis*	2013	Germany	PV999312	PV999413	PV999514	PX025045	PX025151	PX025256	PX025359	PX021823	—	—	This study
*Peronospora agrestis*	WU-MYC0032787	*Veronica filiformis*	2001	Austria	PV999313	PV999414	PV999515	PX025046	PX025152	PX025257	PX025360	PX021824	—	—	This study
*Peronospora aquatica*	GLM-F069669	*Veronica anagallis-aquatica*	1996	Germany	PV999314	PV999415	PV999516	PX025047	PX025153	PX025258	PX025361	PX021825	PX024960	—	This study
*Peronospora aquatica*	GLM-F073366	*Veronica anagallis-aquatica*	2003	Germany	PV999315	PV999416	PV999517	PX025048	PX025154	PX025259	PX025362	PX021826	PX024961	—	This study
*Peronospora aquatica*	GLM-F075951	*Veronica anagallis-aquatica*	2005	Germany	PV999316	PV999417	PV999518	PX025049	PX025155	PX025260	PX025363	PX021827	PX024962	—	This study
*Peronospora aquatica*	ZT81575	*Veronica anagallis-aquatica* (Syn. *V. anagallis*)	1910	Sweden	—	—	—	PX025050	PX025156	—	—	—	—	—	This study
*Peronospora arvensis*	GLM-F062270	*Veronica hederifolia*	2005	Germany	PV999317	PV999418	PV999519	PX025051	PX025157	PX025261	PX025364	PX021828	PX024963	—	This study
*Peronospora arvensis*	GLM-F063010	*Veronica hederifolia*	2004	Germany	PV999318	PV999419	PV999520	PX025052	PX025158	PX025262	PX025365	PX021829	PX024964	—	This study
*Peronospora arvensis*	GLM-F063062	*Veronica hederifolia*	2004	Germany	PV999319	PV999420	PV999521	PX025053	PX025159	PX025263	PX025366	PX021830	PX024965	—	This study
*Peronospora arvensis*	GLM-F067424	*Veronica hederifolia*	1992	Germany	PV999320	PV999421	PV999522	PX025054	PX025160	PX025264	PX025367	PX021831	PX024966	—	This study
*Peronospora arvensis*	GLM-F068053	*Veronica hederifolia*	1993	Germany	PV999321	PV999422	PV999523	PX025055	PX025161	PX025265	PX025368	PX021832	PX024967	—	This study
*Peronospora arvensis*	GLM-F068191	*Veronica hederifolia*	1986	Germany	PV999322	PV999423	PV999524	PX025056	PX025162	PX025266	PX025369	PX021833	PX024968	—	This study
*Peronospora arvensis*	GLM-F069391	*Veronica hederifolia*	1985	Germany	PV999323	PV999424	PV999525	PX025057	PX025163	PX025267	PX025370	PX021834	PX024969	—	This study
*Peronospora arvensis*	GLM-F073461	*Veronica hederifolia*	2003	Germany	PV999324	PV999425	PV999526	PX025058	PX025164	PX025268	PX025371	PX021835	PX024970	—	This study
*Peronospora arvensis*	GLM-F073863	*Veronica hederifolia*	1998	Germany	PV999325	PV999426	PV999527	PX025059	PX025165	PX025269	PX025372	PX021836	PX024971	—	This study
*Peronospora arvensis*	GLM-F074159	*Veronica hederifolia*	2001	Germany	PV999326	PV999427	PV999528	PX025060	PX025166	PX025270	PX025373	PX021837	PX024972	—	This study
*Peronospora arvensis*	GLM-F074167	*Veronica hederifolia*	2001	Germany	PV999327	PV999428	PV999529	PX025061	PX025167	PX025271	PX025374	PX021838	PX024973	—	This study
*Peronospora arvensis*	GLM-F074174	*Veronica hederifolia*	2001	Germany	PV999328	PV999429	PV999530	PX025062	PX025168	PX025272	PX025375	PX021839	PX024974	—	This study
*Peronospora arvensis*	GLM-F074186	*Veronica hederifolia*	2001	Germany	PV999329	PV999430	PV999531	PX025063	PX025169	PX025273	PX025376	PX021840	PX024975	—	This study
*Peronospora arvensis*	GLM-F074206	*Veronica hederifolia*	2001	Germany	PV999330	PV999431	PV999532	PX025064	PX025170	PX025274	PX025377	PX021841	PX024976	—	This study
*Peronospora arvensis*	GLM-F075761	*Veronica hederifolia*	2005	Germany	PV999331	PV999432	PV999533	PX025065	PX025171	PX025275	PX025378	PX021842	PX024977	—	This study
*Peronospora arvensis*	GLM-F076685	*Veronica hederifolia*	2002	Germany	PV999332	PV999433	PV999534	PX025066	PX025172	PX025276	PX025379	PX021843	PX024978	—	This study
*Peronospora arvensis*	GLM-F079120	*Veronica hederifolia*	1990	Germany	PV999333	PV999434	PV999535	PX025067	PX025173	PX025277	PX025380	PX021844	PX024979	—	This study
*Peronospora arvensis*	GLM-F081427	*Veronica hederifolia*	2008	Germany	PV999334	PV999435	PV999536	PX025068	PX025174	PX025278	PX025381	PX021845	PX024980	—	This study
*Peronospora arvensis*	GLM-F081506	*Veronica hederifolia*	2008	Germany	PV999335	PV999436	PV999537	PX025069	PX025175	PX025279	PX025382	PX021846	PX024981	—	This study
*Peronospora arvensis*	GLM-F090770	*Veronica hederifolia*	2008	Germany	PV999336	PV999437	PV999538	PX025070	PX025176	PX025280	PX025383	PX021847	PX024982	—	This study
*Peronospora arvensis*	MG1856	*Veronica hederifolia*	—	Germany	—	AY035491	AY273957	—	DQ365719	DQ361186	—	—	—	—	Riethmüller et al. 2002
*Peronospora arvensis*	ZT81621	*Veronica hederifolia*	1878	Germany	—	—	—	PX025071	PX025177	—	—	PX021848	PX024983	—	This study
*Peronospora arvensis*	ZT81629	*Veronica hederifolia*	1916	Germany	—	—	—	PX025072	PX025178	—	—	—	—	—	This study
*Peronospora arvensis*	WU-MYC0022883	*Veronica triloba*	1999	Austria	AY198244	—	—	—	—	—	—	—	—	—	Voglmayr 2003
*Peronospora brevibrachia*	GLM-F068222	*Veronica chamaedrys*	1988	Germany	PV999337	PV999438	PV999539	PX025073	PX025179	PX025281	PX025384	PX021849	PX024984	—	This study
*Peronospora brevibrachia*	**GLM-F074111**	*Veronica chamaedrys*	1998	Germany	PV999338	PV999439	PV999540	PX025074	PX025180	PX025282	PX025385	PX021850	PX024985	—	This study
*Peronospora brevibrachia* (“*P. agrestis*” misid.)	WU-MYC0022873	*Veronica chamaedrys*	1999	Austria	AY198243	—	—	—	—	—	—	—	—	—	Voglmayr 2003
*Peronospora chionodendron*	GLM-F064157	*Veronica verna*	2004	Germany	PV999363	PV999464	PV999567	PX025099	PX025205	PX025309	PX025412	PX021875	PX025000	—	This study
*Peronospora chionodendron*	GLM-F064162	*Veronica verna*	2004	Germany	PV999364	PV999465	PV999568	PX025100	PX025206	PX025310	PX025413	PX021876	PX025001	—	This study
*Peronospora chionodendron*	GLM-F076762	*Veronica verna*	2002	Germany	PV999365	PV999466	PV999569	PX025101	PX025207	PX025311	PX025414	PX021877	PX025002	—	This study
*Peronospora chionodendron*	**GLM-F076884**	*Veronica verna*	2000	Germany	PV999366	PV999467	PV999570	PX025102	PX025208	PX025312	PX025415	PX021878	PX025003	—	This study
*Peronospora chionodendron*	GLM-F078081	*Veronica verna*	2003	Germany	PV999367	PV999468	PV999571	PX025103	PX025209	PX025313	PX025416	PX021879	PX025004	—	This study
*Peronospora conglomerata*	GLM-F074466	*Geranium pusillum*	2003	Germany	PV919779	PV919807	PV999541	KJ654067	KJ654216	PX025283	PX025386	PV918515	—	—	Choi et al. 2015a, This study
*Peronospora erodii*	GLM-F074456	*Erodium cicutarium*	2003	Germany	PV919782	PV919810	PV999542	KJ654060	KJ654209	PX025284	PX025387	—	—	—	Choi et al. 2015a, This study
*Peronospora fidelia*	**GLM-F063005**	*Veronica triphyllos*	2004	Germany	PV999339	PV999440	PV999543	PX025075	PX025181	PX025285	PX025388	PX021851	—	—	This study
*Peronospora fidelia*	WU-MYC0032801	*Veronica triphyllos*	2011	Austria	PV999340	PV999441	PV999544	PX025076	PX025182	PX025286	PX025389	PX021852	—	—	This study
*Peronospora fidelia*	WU-MYC0032805	*Veronica triphyllos*	2012	Austria	PV999341	PV999442	PV999545	PX025077	PX025183	PX025287	PX025390	PX021853	—	—	This study
*Peronospora fidelia*	WU-MYC0032806	*Veronica triphyllos*	2012	Austria	PV999342	PV999443	PV999546	PX025078	PX025184	PX025288	PX025391	PX021854	—	—	This study
*Peronospora fidelia*	WU-MYC0032808	*Veronica triphyllos*	2012	Austria	PV999343	PV999444	PV999547	PX025079	PX025185	PX025289	PX025392	PX021855	—	—	This study
*Peronospora fidelia*	WU-MYC0032815	*Veronica triphyllos*	2013	Austria	PV999344	PV999445	PV999548	PX025080	PX025186	PX025290	PX025393	PX021856	—	—	This study
*Peronospora gracilis*	**GLM-F069988**	*Veronica praecox*	1990	Germany	PV999345	PV999446	PV999549	PX025081	PX025187	PX025291	PX025394	PX021857	PX024986	—	This study
*Peronospora gracilis*	GLM-F074185	*Veronica praecox*	2001	Germany	PV999346	PV999447	PV999550	PX025082	PX025188	PX025292	PX025395	PX021858	PX024987	—	This study
*Peronospora grisea*	GLM-F063053	*Veronica beccabunga*	2004	Germany	PV999347	PV999448	PV999551	PX025083	PX025189	PX025293	PX025396	PX021859	PX024988	—	This study
*Peronospora grisea*	GLM-F064183	*Veronica beccabunga*	2004	Germany	PV999348	PV999449	PV999552	PX025084	PX025190	PX025294	PX025397	PX021860	PX024989	—	This study
*Peronospora grisea*	GLM-F075648	*Veronica beccabunga*	2005	Germany	PV999349	PV999450	PV999553	PX025085	PX025191	PX025295	PX025398	PX021861	PX024990	—	This study
*Peronospora grisea*	GLM-F075736	*Veronica beccabunga*	2005	Germany	PV999350	PV999451	PV999554	PX025086	PX025192	PX025296	PX025399	PX021862	PX024991	—	This study
*Peronospora grisea*	GLM-F075774	*Veronica beccabunga*	2005	Germany	PV999351	PV999452	PV999555	PX025087	PX025193	PX025297	PX025400	PX021863	PX024992	—	This study
*Peronospora grisea*	GLM-F075832	*Veronica beccabunga*	2005	Germany	PV999352	PV999453	PV999556	PX025088	PX025194	PX025298	PX025401	PX021864	PX024993	—	This study
*Peronospora grisea*	GLM-F076527	*Veronica beccabunga*	2002	Germany	PV999353	PV999454	PV999557	PX025089	PX025195	PX025299	PX025402	PX021865	PX024994	—	This study
*Peronospora grisea*	AR197	*Veronica beccabunga*	—	Germany	—	AY035492	—	—	—	—	—	—	—	—	Riethmüller et al. 2002
*Peronospora grisea*	GLM-F075766	*Veronica serpyllifolia*	2005	Germany	PV999354	PV999455	PV999558	PX025090	PX025196	PX025300	PX025403	PX021866	PX024995	—	This study
*Peronospora grisea*	GLM-F076783	*Veronica serpyllifolia*	2002	Germany	PV999355	PV999456	PV999559	PX025091	PX025197	PX025301	PX025404	PX021867	PX024996	—	This study
*Peronospora grisea*	GLM-F076812	*Veronica serpyllifolia*	2002	Germany	PV999356	PV999457	PV999560	PX025092	PX025198	PX025302	PX025405	PX021868	PX024997	—	This study
*Peronospora grisea*	GLM-F078065	*Veronica serpyllifolia*	2002	Germany	PV999357	PV999458	PV999561	PX025093	PX025199	PX025303	PX025406	PX021869	PX024998	—	This study
*Peronospora grisea*	GLM-F079274	*Veronica serpyllifolia*	2007	Sweden	PV999358	PV999459	PV999562	PX025094	PX025200	PX025304	PX025407	PX021870	PX024999	—	This study
*Peronospora grisea*	WU-MYC0022901	*Veronica serpyllifolia*	2000	Austria	AY198241	—	—	—	—	—	—	—	—	—	Voglmayr 2003
*Peronospora microspora*	**GLM-F073861**	*Veronica teucrium*	1998	Germany	PV999359	PV999460	PV999563	PX025095	PX025201	PX025305	PX025408	PX021871	—	—	This study
*Peronospora microspora*	GLM-F076577	*Veronica teucrium*	2002	Germany	PV999360	PV999461	PV999564	PX025096	PX025202	PX025306	PX025409	PX021872	—	—	This study
*Peronospora microspora*	GLM-F077783	*Veronica teucrium*	1999	Germany	PV999362	PV999462	PV999565	PX025097	PX025203	PX025307	PX025410	PX021873	—	—	This study
*Peronospora microspora*	GLM-F078282	*Veronica teucrium*	1999	Germany	PV999361	PV999463	PV999566	PX025098	PX025204	PX025308	PX025411	PX021874	—	—	This study
*Peronospora obscura*	**GLM-F046912**	*Veronica urticifolia*	2000	Austria	PV999368	PV999469	PV999572	PX025104	PX025210	PX025314	PX025417	PX021880	PX025005	—	This study
*Peronospora obscura*	WU-MYC0032785	*Veronica urticifolia*	2000	Austria	PV999369	PV999470	PV999573	PX025105	PX025211	PX025315	PX025418	PX021881	PX025006	—	This study
*Peronospora obscura*	WU-MYC0032793	*Paederota lutea (*Syn*.Veronica lutea*)	2009	Austria	PV999370	PV999471	PV999574	PX025106	PX025212	PX025316	PX025419	PX021882	PX025007	—	This study
*Peronospora obscura* (“*P. silvestris*” misid.)	AR194	*Veronica urticifolia*	—	Germany	—	AY035490	—	—	—	—	—	—	—	—	Riethmüller et al. 2002
*Peronospora palustris*	GLM-F062903	*Veronica scutellata*	2004	Germany	PV999371	PV999472	PV999575	PX025107	PX025213	PX025317	PX025420	PX021883	PX025008	—	This study
*Peronospora palustris*	GLM-F065747	*Veronica scutellata*	2004	Germany	PV999372	PV999473	PV999576	PX025108	PX025214	PX025318	PX025421	PX021884	PX025009	—	This study
*Peronospora palustris*	GLM-F074295	*Veronica scutellata*	2003	Germany	PV999373	PV999474	PV999577	PX025109	PX025215	PX025319	PX025422	PX021885	PX025010	—	This study
*Peronospora palustris*	T.B.2017	*Veronica armstrongii* (Syn. *Hebe armstrongii*)	2017	Germany	PV999374	PV999475	PV999578	PX025110	PX025216	PX025320	PX025423	—	—	PX024955	This study
*Peronospora palustris*	T.B.2020	*Veronica diosmifolia* (Syn. *Hebe diosmifolia*)	2020	Germany	PV999375	PV999476	PV999579	PX025111	PX025217	PX025321	PX025424	PX021886	PX025011	PX024956	This study
*Peronospora petricosa*	GLM-F063013	*Veronica arvensis*	2004	Germany	PV999376	PV999477	PV999580	PX025112	PX025218	PX025322	PX025425	PX021887	PX025012	—	This study
*Peronospora petricosa*	**GLM-F063014**	*Veronica arvensis*	2004	Germany	PV999377	PV999478	PV999581	PX025113	PX025219	PX025323	PX025426	PX021888	PX025013	—	This study
*Peronospora petricosa*	GLM-F063028	*Veronica arvensis*	2004	Germany	PV999378	PV999479	PV999582	PX025114	PX025220	PX025324	PX025427	PX021889	PX025014	—	This study
*Peronospora petricosa*	GLM-F063058	*Veronica arvensis*	2004	Germany	PV999379	PV999480	PV999583	PX025115	PX025221	PX025325	PX025428	PX021890	PX025015	—	This study
*Peronospora petricosa*	GLM-F065698	*Veronica arvensis*	2005	Germany	PV999380	PV999481	PV999584	PX025116	PX025222	PX025326	PX025429	PX021891	PX025016	—	This study
*Peronospora petricosa*	GLM-F074187	*Veronica arvensis*	2001	Germany	PV999381	PV999482	PV999585	PX025117	PX025223	PX025327	PX025430	PX021892	PX025017	—	This study
*Peronospora petricosa*	GLM-F075720	*Veronica arvensis*	2005	Germany	PV999382	PV999483	PV999586	PX025118	PX025224	PX025328	PX025431	PX021893	PX025018	—	This study
*Peronospora petricosa*	GLM-F075728	*Veronica arvensis*	2005	Germany	PV999383	PV999484	PV999587	PX025119	PX025225	PX025329	PX025432	PX021894	PX025019	—	This study
*Peronospora petricosa*	GLM-F077818	*Veronica arvensis*	1999	Germany	PV999384	PV999485	PV999588	PX025120	PX025226	PX025330	PX025433	PX021895	PX025020	—	This study
*Peronospora petricosa*	GLM-F078032	*Veronica arvensis*	2002	Germany	PV999385	PV999486	PV999589	PX025121	PX025227	PX025331	PX025434	PX021896	PX025021	—	This study
*Peronospora petricosa*	KUS-F17294	*Veronica arvensis*	2000	South Korea	PV999386	PV999487	PV999590	PX025122	PX025228	PX025332	PX025435	PX021897	PX025022	—	This study
*Peronospora petricosa*	KUS-F25712	*Veronica arvensis*	2011	South Korea	PV999387	PV999488	PV999591	PX025123	PX025229	PX025333	PX025436	PX021898	PX025023	—	This study
*Peronospora petricosa*	R.D.854	*Veronica arvensis*	1991	Argentina	PV999388	PV999489	PV999592	PX025124	PX025230	PX025334	PX025437	PX021899	PX025024	—	This study
*Peronospora petricosa*	WU-MYC0032799	*Veronica arvensis*	2011	Austria	PV999389	PV999490	PV999593	PX025125	PX025231	PX025335	PX025438	PX021900	PX025025	—	This study
*Peronospora petricosa*	DAR69945	*Veronica odora* (Syn. *Hebe buxifolia*)	—	Australia	PV999390	PV999491	PV999594	PX025126	PX025232	PX025336	PX025439	PX021901	—	—	This study
*Peronospora petricosa*	ZT81370	*Veronica arvensis*	1941	Switzerland	—	—	—	PX025127	PX025233	—	—	PX021902	—	—	This study
*Peronospora petricosa* (“*P. verna*” misid.)	MG1969	*Veronica arvensis*	2001	Germany	—	AY271994	—	—	DQ365735	—	—	—	—	—	Göker et al. 2003
*Peronospora sagittaria*	**GLM-F074298**	*Veronica catenata*	2003	Germany	PV999391	PV999492	PV999595	PX025128	PX025234	PX025337	PX025440	PX021903	—	—	This study
*Peronospora sagittaria*	GLM-F075865	*Veronica catenata*	2005	Germany	PV999392	PV999493	PV999596	PX025129	PX025235	PX025338	PX025441	PX021904	PX025026	—	This study
*Peronospora sagittaria* (“*P. aquatica*” misid.)	MG1968	*Veronica anagallis-aquatica*	—	Germany	—	AY271991	AY273956	—	DQ365718	DQ361185	—	—	—	—	Göker et al. 2003
*Peronospora seminaria*	**GLM-F125572**	*Veronica longifolia*	2023	Germany	PV999393	PV999494	PV999597	PX025130	PX025236	PX025339	PX025442	PX021905	PX025027	PX024957	This study
*Peronospora seminaria*	WU-MYC0032804	*Veronica longifolia*	2011	Czech Republic	PV999394	PV999495	PV999598	PX025131	PX025237	PX025340	PX025443	PX021906	PX025028	—	This study
*Peronospora seminaria*	WU-MYC0032814	*Veronica longifolia*	2012	Austria	PV999395	PV999496	PV999599	PX025132	PX025238	PX025341	PX025444	PX021907	PX025029	—	This study
*Peronospora silvestris*	WU-MYC0032802	*Veronica officinalis*	2011	Austria	PV999396	PV999497	PV999600	PX025133	PX025239	PX025342	PX025445	PX021908	PX025030	—	This study
*Peronospora silvestris*	WU-MYC0032803	*Veronica officinalis*	2011	Austria	PV999397	PV999498	PV999601	PX025134	PX025240	PX025343	PX025446	PX021909	PX025031	—	This study
*Peronospora silvestris*	GLM-F135692	*Globularia nudicaulis*	2016	Switzerland	PV999398	PV999499	PV999602	PX025135	PX025241	PX025344	PX025447	PX021910	—	—	This study
*Peronospora silvestris*	GLM-F135693	*Globularia nudicaulis*	2016	Switzerland	PV999399	PV999500	PV999603	PX025136	PX025242	PX025345	PX025448	PX021911	—	—	This study
*Peronospora* sp.	WU-MYC0032791	*Veronica triloba*	2004	Austria	PV999400	PV999501	PV999604	PX025137	PX025243	PX025346	PX025449	PX021912	PX025032	—	This study
*Peronospora* sp.	WU-MYC0032807	*Veronica triloba*	2012	Austria	PV999401	PV999502	PV999605	PX025138	PX025244	PX025347	PX025450	PX021913	PX025033	—	This study
*Peronospora veronicae-cymbalariae*	WU-MYC0032796	*Veronica cymbalaria*	2011	Spain	PV999402	PV999503	PV999606	PX025139	PX025245	PX025348	PX025451	PX021914	PX025034	—	This study
*Peronospora veronicae-cymbalariae*	WU-MYC0032810	*Veronica cymbalaria*	2012	Greece	PV999403	PV999504	PV999607	PX025140	PX025246	PX025349	PX025452	PX021915	PX025035	—	This study
*Peronospora veronicae-cymbalariae*	WU-MYC0032812	*Veronica cymbalaria*	2012	Greece	PV999404	PV999505	PV999608	PX025141	PX025247	PX025350	PX025453	PX021916	PX025036	—	This study
*Peronospora veronicae-cymbalariae*	WU-MYC0032813	*Veronica cymbalaria*	2012	Greece	PV999405	PV999506	PV999609	PX025142	PX025248	PX025351	PX025454	PX021917	PX025037	—	This study
*Peronospora veronicae-cymbalariae*	**ZT85386**	*Veronica cymbalaria*	1937	Palestine	—	—	—	PX025143	—	—	—	—	—	—	This study
*Pseudoperonospora cubensis*	GLM-F049006	*Cucumis sp*.	1997	Germany	MH730807	MH730848	MH730828	MH730767	MH730773	MH730869	MH730893	—	—	—	Thines et al. 2019
Holotypes are printed in bold.															

### Morphology

Morphological characteristics of conidiophores, conidia, and oospores were examined using dried herbarium specimens, following the methodology outlined by Mu et al. (2024a). In summary, conidiophores were scraped from dried herbarium specimens into a lactic acid solution, heated shortly to the boiling point, and mounted under coverslips for observation with a Zeiss Imager M2 microscope (Zeiss, Oberkochen, Germany) equipped with differential interference contrast. Measurements are reported as (minimum–) standard deviation towards the minimum–mean–standard deviation towards the maximum (–maximum). The length-to-breadth ratio of conidia and the longer-to-shorter ratio of the ultimate branchlets were rounded to the nearest 0.05; the other measured values were rounded to the nearest 0.5 µm.

### DNA extraction, PCR, and sequencing

Genomic DNA was extracted using the BioSprint 96 DNA Plant Kit (Qiagen, Venlo, Netherlands) on a KingFisher Flex (Thermo Scientific, Waltham, MS, USA) automated extraction instrument, according to the protocol provided by the manufacturer. For older herbarium specimens (~100 years old) from the ZT collection, Proteinase K was added during the lysis step.

Two nuclear loci (ITS, nrLSU) and five mitochondrial loci (*COX*1, *COX*2, *COX*2-1 spacer, *NAD1*, and *RPS10*) were amplified using oomycete-specific primers, as previously outlined by Choi et al. (2015b). For the host DNA, PCR amplification was performed with the primers matK-xf (Ford et al. 2009) and matK-MALP (Dunning and Savolainen 2010) for the *matK* regions, and the primers ITS1-Plant and ITS4 (Navarro et al. 2003; White et al. 1990, respectively) for the ITS regions, and the primers trnL_c and trnF_f (Taberlet et al. 1991) for the *trnL-F* region. Amplicons were bidirectionally sequenced at the Senckenberg Biodiversity and Climate Research Centre (SBiK-F, Frankfurt, Germany) using the same primers as those used for the initial amplification.

### Phylogenetic analyses

The sequences obtained in this study were edited and assembled using Geneious v5.6 (Biomatters, Auckland, New Zealand). Alignments were conducted using MAFFT v7 (Katoh and Standley 2013) and employing the Q-INS-i algorithm (Katoh and Toh 2008). Phylogenetic analyses in this study followed the methods described by Mu et al. (2024a, b). In short, TrEase (Mishra et al. 2023) was used for initial computation of phylogenetic trees using standard settings, and subsequently, the standalone version of RAxML v7 (Stamatakis 2006) was used for Maximum Likelihood (ML) analyses, with 1,000 rounds of random addition of sequences and 1,000 fast bootstrap replicates. MEGA v7 (Kumar et al. 2016) was used for Minimum Evolution (ME) inference with default settings, except for using the Tamura-Nei substitution model and treating indels as pairwise deletion. Support for internal nodes was estimated by 1,000 bootstrap replicates (Felsenstein 1985). MrBayes v3.1.6 (Ronquist et al. 2012) was used for Bayesian inference (BI). The best-fitting model of sequence evolution approximating GTR was selected using the Akaike Information Criterion (AIC) as implemented in MrModeltest version 2.3 (Nylander 2004). BI Analyses were run for 8 million generations, with trees sampled every 10,000 generations. The first 30% of the trees were discarded as burn-in, and the posterior probabilities were calculated from the remaining trees to ensure sampling from the stationary phase. *Pseudoperonospora cubensis* served as the outgroup for rooting phylogenetic reconstructions.

## Results

### Morphology

The morphological measurements of *Peronospora* species on *Veronica* are summarised in Table 3. Key morphological traits distinguishing the *Peronospora* species include conidial shape, conidiophore and trunk length, as well as the length of the ultimate branchlets. *Peronospora agrestis* on *Veronica polita*, *Veronica filiformis*, and *Veronica persica* had the smallest longer-to-shorter ratio of ultimate branchlet lengths, 1.4 on average (Fig. 2); *Peronospora aquatica* on *Veronica anagallis-aquatica* had the largest conidia, 33 × 21 µm on average, as well as the broadest trunks, up to 12 µm broad and conidiophore bases, up to 17 µm broad (Fig. 3); *Peronospora arvensis* on *Veronica hederifolia* only reached about 6 orders of subdichotomous branching (Fig. 4). *Peronospora* sp. on *Veronica chamaedrys* had the shortest ultimate branchlets (the longer ones 10 µm long, the shorter ones 7 µm long) (Fig. 5); *Peronospora* sp. on *Veronica verna* had subglobose conidia, with an average length-to-breadth ratio of 1.25 (Fig. 6). *Peronospora* sp. on *Veronica triphyllos* had rather short conidiophores (95 µm on average) and trunks (171 µm on average) (Fig. 7); *Peronospora* sp. on *Veronica praecox* had subglobose conidia, with an average length-to-breadth ratio of 1.25 (Fig. 8). *Peronospora grisea* on *Veronica beccabunga* had similarly short conidiophores, 293 µm long on average, and trunks 165 µm long on average (Fig. 9); *Peronospora* sp. on *Veronica teucrium* had the smallest conidia, 19.5 × 15.5 µm on average (Fig. 10); *Peronospora* sp. on *Veronica urticifolia* and *Paederota lutea* had shorter trunks, 165 µm on average, and higher conidiophores-to-trunks ratio, up to 3.1 (Fig. 11); *Peronospora palustris* on *Veronica scutellata* had the longest conidiophores, 443 µm long on average, up to 648 µm long, and trunks 299 µm long on average, up to 469 µm long, as well as the most elongate conidia, with an average length-to-breadth ratio of 1.7 (Fig. 12); *Peronospora* sp. on *Veronica arvensis* had the narrowest trunks, 6 µm broad on average, as well as small conidia, 20 × 15.5 µm on average (Fig. 13); *Peronospora* sp. on *Veronica catenata* had the longest ultimate branchlets, longer ones up to 32.6 µm long, the shorter ones up to 25 µm long, as well as larger conidia, 32 × 21 µm on average (Fig. 14); *Peronospora* sp. on *Veronica longifolia* had more elongated conidia, with an average length-to-breadth ratio of 1.6, which are the narrowest among the species investigated, 14 µm on average (Fig. 15); *Peronospora silvestris* on *Veronica officinalis* and *Globularia nudicaulis* had similarly more elongated conidia, with an average length-to-breadth ratio of 1.6 (Fig. 16); *Peronospora veronicae-cymbalariae* on *Veronica cymbalaria* had the shortest conidiophores and trunks, 246 µm and 117 µm on average, respectively, the highest conidiophores-to-trunks ratio, up to 3.3, as well as larger conidia, 32 × 23 µm on average, which are the broadest among the species investigated, up to 32 µm broad (Fig. 17); *Peronospora saxatilis* on *Veronica fruticans* had larger conidia as well, 30.5 × 22.5 µm on average (Gäumann 1918).

**Table 3.** Morphological comparison of species of *Peronospora* on *Veronica*.

Species	Host	Conidiophores (n = 50)		Trunks (n = 50)		Conidiophores/trunks	Branching orders	
		Length (μm)	Base (μm)	Length (μm)	Breadth (μm)			
*Peronospora agrestis*	*Veronica polita*, *V. filiformis*, *V. persica*	(221–)271–334–396(−453)	9–11–13	(115–)156–200–243(−284)	(5.0–)6.4–7.7–9.1(−9.5)	(1.4–)1.5–1.7–1.9(−2.1)	4–6	
*Peronospora aquatica*	*Veronica anagallis-aquatica*	(207–)276–348–420(−486)	9–13–17	(119–)146–190–234(−267)	(4.5–)7.3–9.1–11.0(−12.2)	(1.4–)1.6–1.9–2.2(−2.7)	4–8	
*Peronospora arvensis*	*Veronica hederifolia*	(286–)287–347–406(−483)	11–12–13	(155–)168–214–260(−318)	(6.3–)6.8–8.1–9.4(−10.1)	(1.4–)1.5–1.6–1.8(−2.0)	4–6	
*Peronospora brevibrachia*	*Veronica chamaedrys*	(276–)313–380–447(−497)	8–10–12	(117–)177–240–304(−365)	(4.2–)5.5–7.0–8.5(−9.6)	(1.3–)1.4–1.6–1.9(−2.5)	4–8	
*Peronospora chionodendron*	*Veronica verna*	(205–)255–312–369(−461)	8–10–12	(102–)137–176–216(−265)	(6.5–)6.7–8.3–9.9(−11.3)	(1.4–)1.6–1.8–2.0(−2.6)	4–7	
*Peronospora fidelia*	*Veronica triphyllos*	(210–)238–295–352(−428)	9–11–14	(80–)123–171–220(−268)	(6.6–)7.0–7.8–8.6(−8.8)	(1.4–)1.5–1.8–2.1(−2.7)	4–6	
*Peronospora gracilis*	*Veronica praecox*	(237–)266–298–329(−355)	8–12–16	(102–)139–172–205(−241)	(3.9–)4.8–6.6–8.5(−9.7)	(1.4–)1.5–1.8–2.1(−2.8)	4–6	
*Peronospora grisea*	*Veronica beccabunga*, *V. serpyllifolia*	(203–)250–293–335(−379)	8–10–13	(95–)122–165–207(−267)	(6.1–)6.7–7.5–8.2(−8.4)	(1.3–)1.5–1.8–2.2(−2.7)	4–7	
*Peronospora microspora*	*Veronica teucrium*	(180–)245–295–346(−394)	7–10–12	(64–)135–179–223(−267)	(4.5–)5.3–6.5–7.7(−9.4)	(1.2–)1.4–1.7–2.0(−2.8)	4–6	
*Peronospora obscura*	*Veronica urticifolia*, *Paederota lutea*	(248–)279–330–380(−452)	8–10–12	(108–)130–165–199(−267)	(5.4–)5.2–7.2–9.1(−11.6)	(1.5–)1.7–2.1–2.5(−3.1)	4–7	
*Peronospora palustris*	*Veronica scutellata*	(283–)335–443–550(−648)	9–11–14	(174–)204–299–394(−469)	(4.3–)5.9–7.6–9.3(−10.3)	(1.2–)1.3–1.5–1.8(−2.1)	4–8	
*Peronospora petricosa*	*Veronica arvensis*	(214–)237–295–353(−453)	8–11–15	(97–)130–176–222(−346)	(4.4–)5.1–6.1–7.1(−8.3)	(1.0–)1.4–1.7–2.0(−2.4)	4–7	
*Peronospora sagittaria*	*Veronica catenata*	(247–)319–405–490(−628)	7–11–15	(122–)178–243–307(−406)	(6.1–)6.3–8.0–9.7(−11.1)	(1.3–)1.5–1.7–1.9(−2.2)	4–7	
*Peronospora seminaria*	*Veronica longifolia*	(159–)221–365–508(–561)	5–10–16	(80–)103–180–257(–429)	(5.8–)6.4–7.9–9.5(–10.4)	(1.3–)1.5–1.8–2.1(–2.5)	4–8	
*Peronospora silvestris*	*Veronica officinalis*, *Globularia nudicaulis*	(269–)332–411–490(−571)	9–12–14	(107–)203–256–309(−326)	(4.8–)6.0–7.8–9.6(−10.1)	(1.3–)1.4–1.6–1.9(−2.5)	4–7	
*Peronospora veronicae-cymbalariae*	*Veronica cymbalaria*	(166–)208–246–284(–332)	9–12–16	(61–)88–117–145(–185)	(6.1–)8.1–9.8–11.4(–14.3)	(1.5–)1.8–2.2–2.6(–3.3)	4–5	
Species	Host	Ultimate Branchlets (UB) (n = 100)			Conidia (n = 100)			Oospores (μm)
		Length of the longer UB (μm)	Length of the shorter UB (μm)	Longer/shorter	Length (μm)	Breadth (μm)	Length/breadth	
*Peronospora agrestis*	*Veronica polita*, *V. filiformis*, *V. persica*	(5.97–)9.27–12.37–15.47(−19.28)	(4.19–)6.54–8.84–11.15(−13.85)	(1.06–)1.21–1.42–1.63(−1.88)	(18.70–)19.92–21.22–22.52(−23.52)	(14.04–)14.94–16.01–17.08(−17.94)	(1.13–)1.24–1.33–1.42(−1.49)	—
*Peronospora aquatica*	*Veronica anagallis-aquatica*	(8.81–)12.79–17.12–21.46(−26.26)	(5.74–)7.81–10.28–12.75(−15.84)	(1.21–)1.52–1.74–1.95(−2.19)	(18.14–)29.43–33.21–37.00(−39.66)	(9.23–)18.41–21.24–24.07(−25.96)	(1.33–)1.43–1.58–1.72(−1.97)	28.1–30.5–32.9
*Peronospora arvensis*	*Veronica hederifolia*	(8.61–)10.59–14.03–17.46(−21.26)	(4.86–)6.55–8.94–11.33(−12.52)	(1.10–)1.31–1.63–1.95(−2.24)	(24.53–)24.81–26.36–27.91(−29.41)	(18.89–)19.03–20.17–21.30(−22.36)	(1.15–)1.23–1.31–1.39(−1.50)	34.3–38.6–41.3
*Peronospora brevibrachia*	*Veronica chamaedrys*	(6.37–)8.18–10.06–11.94(−14.85)	(3.41–)5.24–7.03–8.83(−10.38)	(1.05–)1.22–1.48–1.74(−2.14)	(18.94–)20.62–21.90–23.19(−23.95)	(13.44–)14.90–15.94–16.98(−17.31)	(1.23–)1.24–1.38–1.52(−1.65)	—
*Peronospora chionodendron*	*Veronica verna*	(6.41–)8.53–11.21–13.89(−17.50)	(4.60–)5.71–7.72–9.72(−13.09)	(1.01–)1.25–1.47–1.70(−1.92)	(17.00–)19.29–21.14–22.98(−25.11)	(14.22–)15.60–16.93–18.26(−19.91)	(1.06–)1.16–1.25–1.34(−1.42)	—
*Peronospora fidelia*	*Veronica triphyllos*	(8.10–)9.92–12.81–15.70(−18.70)	(5.56–)6.33–8.23–10.14(−14.03)	(1.11–)1.32–1.58–1.83(−2.30)	(16.89–)19.99–22.48–24.97(−27.25)	(13.52–)15.24–16.94–18.64(−20.03)	(1.12–)1.19–1.33–1.48(−1.68)	36.0–40.5–44.2
*Peronospora gracilis*	*Veronica praecox*	(6.88–)8.44–11.03–13.62(−17.88)	(5.09–)5.91–7.51–9.10(−10.92)	(1.05–)1.23–1.49–1.74(−2.06)	(16.73–)18.82–21.14–23.45(−26.85)	(14.31–)15.29–16.59–17.90(−19.52)	(1.11–)1.17–1.27–1.38(−1.59)	—
*Peronospora grisea*	*Veronica beccabunga*, *V. serpyllifolia*	(8.17–)8.87–11.35–13.84(−17.69)	(4.71–)5.96–8.03–10.11(−11.72)	(1.07–)1.22–1.44–1.67(−2.00)	(18.21–)20.14–22.52–24.91(−27.29)	(13.25–)15.05–16.43–17.80(−18.70)	(1.11–)1.24–1.38–1.52(−1.56)	—
*Peronospora microspora*	*Veronica teucrium*	(6.44–)8.98–11.84–14.69(−17.75)	(3.68–)5.91–8.06–10.22(−14.53)	(1.02–)1.20–1.51–1.82(−2.25)	(15.29–)17.20–19.34–21.48(−23.94)	(12.67–)13.52–15.28–17.04(−19.84)	(1.12–)1.16–1.27–1.38(−1.49)	35.03–41.0–49.4
*Peronospora obscura*	*Veronica urticifolia*, *Paederota lutea*	(7.67–)9.26–12.39–15.52(−19.70)	(5.06–)5.38–7.91–10.43(−16.05)	(1.03–)1.31–1.61–1.91(−2.32)	(23.40–)24.90–26.64–28.38(−30.73)	(16.67–)17.51–18.42–19.33(−21.06)	(1.21–)1.34–1.45–1.56(−1.72)	—
*Peronospora palustris*	*Veronica scutellata*	(8.70–)9.81–12.15–14.50(−17.28)	(5.38–)5.88–7.24–8.59(−10.18)	(1.25–)1.47–1.69–1.91(−2.13)	(23.53–)26.22–28.87–31.52(−33.55)	(14.40–)15.22–17.48–19.75(−24.42)	(1.08–)1.41–1.68–1.94(−2.08)	—
*Peronospora petricosa*	*Veronica arvensis*	(6.57–)8.11–11.04–13.98(−19.05)	(5.02–)6.02–8.19–10.37(−14.01)	(1.02–)1.15–1.36–1.57(−1.78)	(16.69–)18.72–20.23–21.75(−23.33)	(13.29–)14.33–15.64–16.94(−18.00)	(1.12–)1.19–1.30–1.41(−1.60)	—
*Peronospora sagittaria*	*Veronica catenata*	(8.78–)12.08–17.72–23.36(−32.63)	(5.64–)7.32–10.87–14.42(−25.00)	(1.06–)1.32–1.61–1.90(−2.43)	(23.97–)28.72–31.87–35.03(−36.70)	(16.67–)18.79–21.11–23.43(−27.54)	(1.18–)1.34–1.52–1.70(−2.00)	—
*Peronospora seminaria*	*Veronica longifolia*	(5.47–)7.81–10.61–13.42(–15.60)	(3.16–)4.95–7.33–9.71(–12.10)	(1.05–)1.28–1.49–1.71(–1.88)	(13.74–)17.15–22.14–27.13(–34.26)	(10.07–)10.70–13.78–16.87(–21.44)	(1.25–)1.46–1.61–1.77(–2.00)	—
*Peronospora silvestris*	*Veronica officinalis*, *Globularia nudicaulis*	(7.83–)9.58–11.94–14.30(−16.19)	(5.67–)5.94–7.31–8.67(−10.64)	(1.23–)1.41–1.65–1.89(−2.06)	(20.19–)24.09–27.54–31.00(−32.30)	(13.23–)14.79–17.34–19.89(−25.98)	(1.04–)1.38–1.61–1.84(−2.08)	—
*Peronospora veronicae-cymbalariae*	*Veronica cymbalaria*	(7.60–)11.05–14.55–18.06(–22.52)	(5.39–)7.23–9.71–12.19(–15.08)	(1.03–)1.29–1.52–1.75(–2.30)	(21.19–)27.41–31.96–36.51(–42.66)	(16.59–)19.90–22.95–25.99(–31.98)	(1.01–)1.21–1.40–1.59(–1.80)	30.0–44.4–56.8

### Phylogenetic analyses

**Figure 1.**
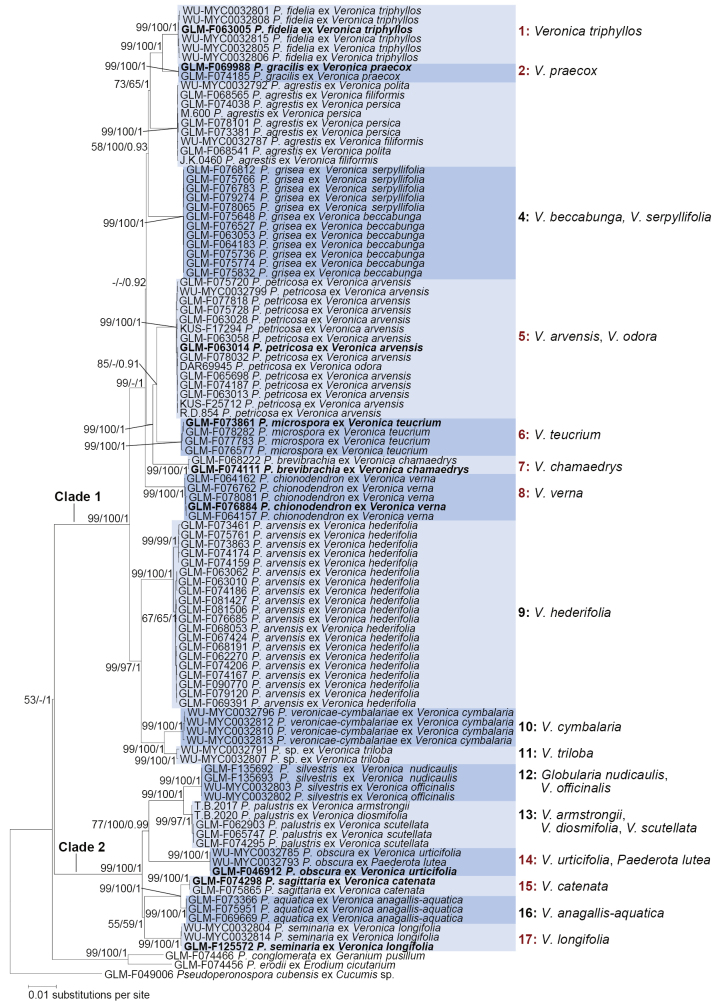
Phylogenetic tree inferred from Maximum Likelihood analysis of a concatenated alignment of two nuclear loci (ITS and nrLSU) and five mitochondrial loci (*COX*1, *COX*2, *COX*2-1 spacer, *NAD1*, *RPS10*), with *Pseudoperonospora cubensis* as outgroup. Red clade numbers indicate new species. Bootstrap support values of Minimum Evolution and Maximum Likelihood methods, as well as Bayesian posterior probabilities are indicated along branches, in the respective order. The scale bar denotes the number of nucleotide substitutions per site. Ex-holotype sequences of the new species described in this study are printed in bold type.

The phylogenetic reconstructions based on individual gene trees (Additional file 1), derived from two nuclear loci (ITS, nrLSU) and five mitochondrial loci (*COX*1, *COX*2, *COX*2-1 spacer, *NAD1*, *RPS10*), showed no strong support for conflicting topologies. Thus, the alignments were concatenated for phylogenetic inference; the resulting alignment included 104 sequences and spanned 5,767 aligned sites. Phylogenetic inference using Minimum Evolution (ME), Maximum Likelihood (ML), and Bayesian Inference (BI) on the same dataset showed no significant topological conflicts, thus, only the tree from the ML inference is shown in Fig. 1, with support values from all three analyses. The phylogenetic reconstruction revealed 17 lineages of *Peronospora* spp. on *Veronica*, comprising 7 described species, one newly combined species, and 10 previously undescribed species. As one lineage was represented only by DNA sequences and lacked specimens for morphological analysis, only 9 new species are described in this study.

### Taxonomy

Based on phylogenetic reconstructions and morphological differences, 16 species were accepted in this study. These include seven previously described species and nine new species of *Peronospora* on *Veronica*, which are presented in alphabetical order below.

#### Peronospora agrestis

Taxon classificationFungiPeronosporalesPeronosporaceae

Gäum., Annales Mycologici 16 (1–2): 198. 1918.

F521E4B6-492B-5A02-8310-BEBF3F1B8286

216046

Fig. 2

##### Description.

***Lesions*** on leaves at first yellowish, later dark yellow to brown, diffuse to vein-delimited. ***Down*** present on the lower leaf surface, consisting of scattered to dense felt-like conidiophore outgrowth. ***Conidiophores*** hyaline, straight to curved, thin-walled, 271–396 µm long, av. 334 µm; trunk 156–243 µm long, av. 200 µm, 6.5–9 µm broad, sometimes slightly swollen to up to 13 µm at the base, ratio of the total length to trunk length 1.5–2, callose plugs absent. ***Branching*** subdichotomous in 4–6 orders, with sub-straight to curved branches, gradually attenuate, often at right angles. ***Ultimate branchlets*** sub-straight to curved, paired branchlets differing in length, with the longer ones 9.5–15.5 µm long, the shorter ones 6.5–11 µm long, with a ratio of the longer to the shorter ultimate branchlet of 1.2–1.65, base 2–2.5 µm broad, apex slightly sharp. ***Conidia*** ellipsoid to ovoid with a dark brown colour, 20–22.5 µm long, 15–17 µm broad, length-to-breadth ratio 1.25–1.4, basal part of the conidia mostly protruding. ***Oospores*** not observed.

**Figure 2.**
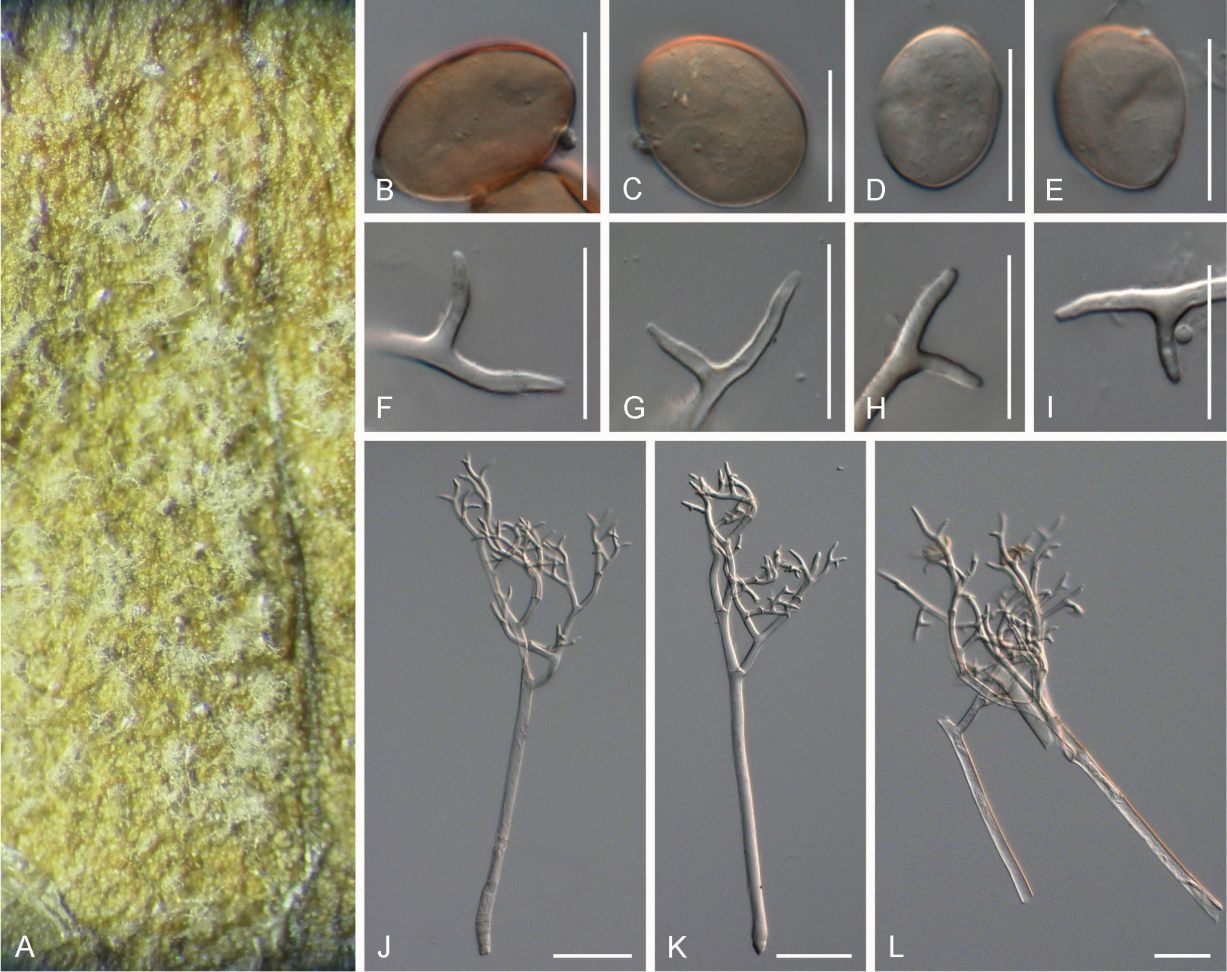
Symptoms and morphology of *Peronospora agrestis* parasitic on *Veronica polita* (GLM-F068541). **A** Down on the leaf surface. **B–E** Conidia. **F–I** Ultimate branchlets. **J–L** Conidiophores. Scale bars: 20 µm (**B–I**); 50 µm (**J–L**).

##### Type host.

*Veronica polita* (Gäumann 1918; Stevenson 1926).

##### Reported hosts.

*Veronica agrestis* (Gäumann 1918; Stevenson 1926; Dennis 1986), *V. arvensis* (Dennis 1986), *V. polita* (Gäumann 1918; Stevenson 1926), *V. opaca* (Notizen 1934), *Veronica chamaedrys* (Voglmayr 2003).

##### Reported distribution.

Austria, Russia, Spain, Switzerland (Gäumann 1923; Stevenson 1926; Voglmayr 2003).

##### Specimens examined.

AUSTRIA. Lower Austria: Mödling, Guntramsdorf, gappy embankment at Weingarten, on living leaves of *Veronica polita*, 4 Apr 2004, H. Voglmayr (WU-MYC0032792). GERMANY. Baden-Württemberg: Tübingen, on living leaves of *Veronica filiformis*, 28 Apr 2001, H. Voglmayr (WU-MYC0032787); Bayern: unknown, on living leaves of *Veronica filiformis*, 10 May 2013, J. Kruse (J.K.0460); Saxony: Dresden, on living leaves of *Veronica filiformis*, 11 July 1988, H. Jage (GLM-F068565); Hermsdorf, on living leaves of *Veronica persica*, 28 Jun 1998, H. Jage (GLM-F074038); Saxony-Anhalt: Lammsdorf, on living leaves of *Veronica persica*, 25 May 2002, H. Jage (GLM-F078101); Kemberg, on living leaves of *Veronica polita*, 29 Sep 1984, H. Jage (GLM-F068541); Saaleck, on living leaves of *Veronica persica*, 19 Jun 2003, H. Jage (GLM-F073381). Unknown, on living leaves of *Veronica persica*, unknown, M. Thines (M.600).

##### Notes.

*Peronospora agrestis* has been reported in Europe infecting various hosts, including *Veronica agrestis*, *V. arvensis*, *V. chamaedrys*, *V. opaca*, and *V. polita* (Gäumann 1918, 1923; Stevenson 1926; Notizen 1934; Dennis 1986; Voglmayr 2003), with *V. polita* being the type host of *P. agrestis*. However, our phylogenetic analysis reveals that *P. agrestis* is restricted to its type host, *V. polita*, and closely related hosts *V. filiformis* and *V. persica*. The *Peronospora* species found on *V. arvensis* represents a distinct, previously undescribed taxon, which is phylogenetically distinct from *P. agrestis* infecting *V. polita*, *V. filiformis*, and *V. persica* across all phylogenetic trees. Furthermore, an ITS sequence, AY198243, from specimen WU-MYC0022873 collected in Austria on *Veronica chamaedrys* was previously identified as *P. agrestis* (Voglmayr 2003). However, our ITS phylogenetic tree shows that this sequence does not form a clade with *P. agrestis*, but rather with two other specimens from Germany, also parasitic on *V. chamaedrys*, suggesting that *V. chamaedrys* is not a host of *P. agrestis*, but a previously undescribed species. Phylogenetic analyses indicate that *P. agrestis* has two closely related sister species, *P. fidelia* and *P. gracilis*, which were previously undescribed. However, *P. fidelia* and *P. gracilis* are characterised by shorter conidiophores and trunks, with lengths shorter than 300 µm and 200 µm on average, respectively.

#### Peronospora aquatica

Taxon classificationFungiPeronosporalesPeronosporaceae

Gäum., Annales Mycologici 16 (1–2): 199. 1918.

54626B00-BC68-5AE0-A3ED-7855F0E4CEB2

220784

Fig. 3

##### Description.

***Lesions*** on leaves at first yellow to brown then darken to become reddish, diffuse to vein-delimited. ***Down*** present on the lower leaf surface, consisting of scattered to dense felt-like conidiophore outgrowth. ***Conidiophores*** hyaline, straight to curved, thin-walled, 276–420 µm long, av. 348 µm; trunk 146–234 µm long, av. 190 µm, 7.5–11 µm broad, sometimes slightly swollen to up to 17 µm at the base, ratio of the total length to trunk length 1.5–2, callose plugs absent. ***Branching*** subdichotomous in 4–8 orders, with sub-straight to curved branches, gradually attenuate, often at acute angles. ***Ultimate branchlets*** sub-straight to curved, paired branchlets differing in length, with the longer ones 13–21.5 µm long, the shorter ones 8–13 µm long, with a ratio of the longer to the shorter ultimate branchlet of 1.5–2, base 2.5–3.5 µm broad, apex slightly sharp. ***Conidia*** ellipsoid to elongated with a pale brown to reddish colour, 29.5–37 µm long, 18.5–24 µm broad, length-to-breadth ratio 1.45–1.7, basal part of the conidia mostly protruding. ***Oospores*** 28–33 µm in diameter.

**Figure 3.**
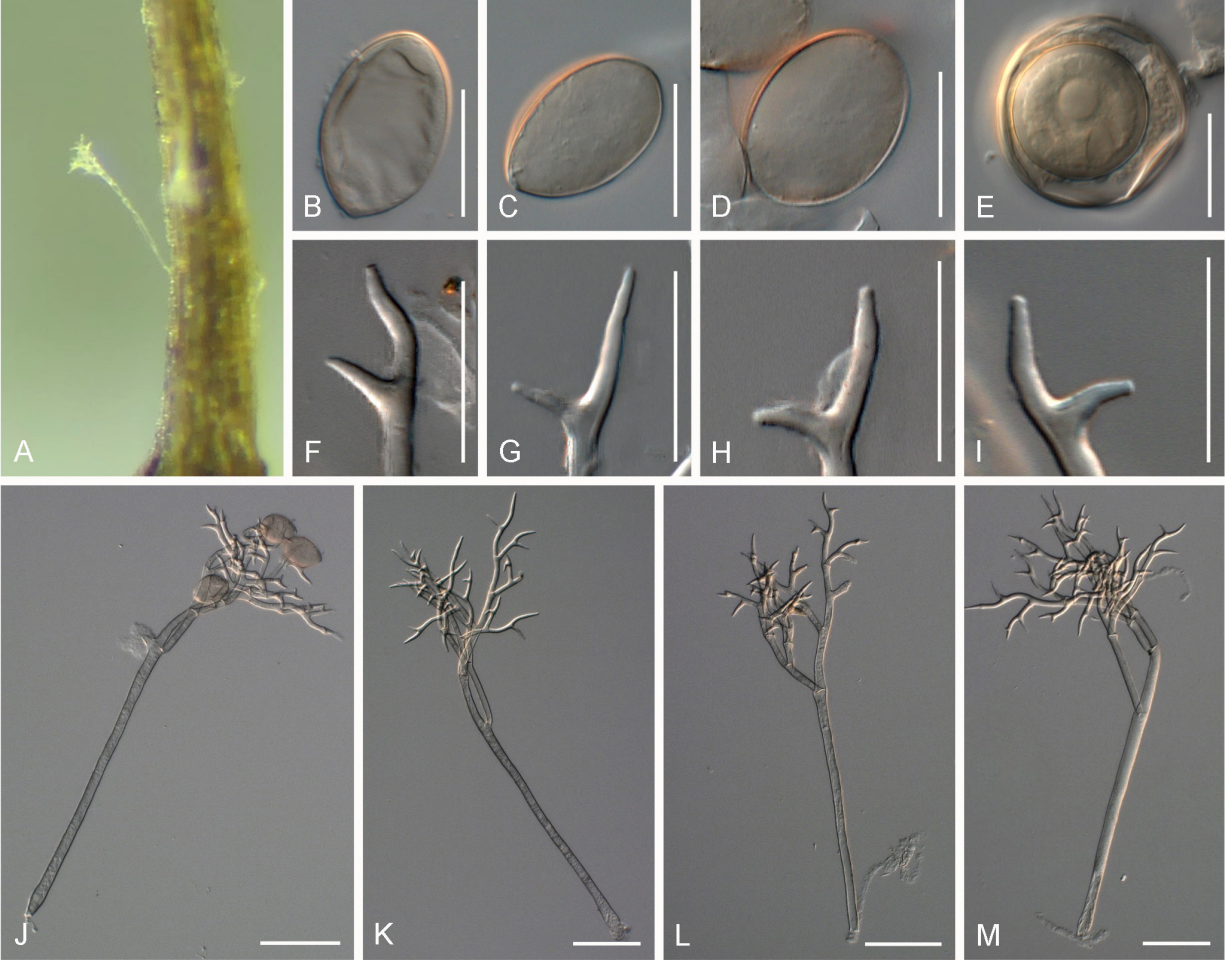
Symptoms and morphology of *Peronospora aquatica* parasitic on *Veronica anagallis-aquatica* (GLM-F075951). **A** Down on the leaf surface. **B–D** Conidia. **E** Oospores. **F–I** Ultimate branchlets. **J–M** Conidiophores. Scale bars: 20 µm (**B–I**); 50 µm (**J–M**).

##### Type host.

*Veronica anagallis-aquatica* (cited under its orthographic variant *Veronica anagallis* Cham. & Schltdl.) (Gäumann 1918).

##### Reported distribution.

Austria, Germany, Poland, Switzerland, Sweden, United States, China, Denmark, France, Latvia, Lithuania, Romania, Russia (Gäumann 1923; Yu 1998; Göker et al. 2003, 2007).

##### Specimens examined.

GERMANY. Saxony-Anhalt: Helme-Unstrut Buntsandsteinland, on living leaves of *Veronica anagallis-aquatica*, 29 May 2003, H. Jage (GLM-F073366); Querfurt Plate, Lodersleben, at the castle, in the Querne, on living leaves of *Veronica anagallis-aquatica*, 6 May 2005, H. Jage (GLM-F075951); Trebitz, Krähenberg, on living leaves of *Veronica anagallis-aquatica*, 22 Aug 1996, H. Jage (GLM-F069669). SWEDEN. Gotland: unknown, on living leaves of *Veronica anagallis-aquatica*, 20 Jul 1910, T. Vestergren (ZT81575).

##### Notes.

*Peronospora aquatica* is widely reported to be distributed across Europe, Asia, and North America, where it parasitises *Veronica anagallis-aquatica* (=*Veronica anagallis* Cham. & Schltdl.) (Gäumann 1918; Gäumann 1923; Yu 1998; Göker et al. 2003, 2007). Phylogenetic analyses reveal that *P. aquatica* has a closely related sister species, *P. sagittaria*, which was previously undescribed and parasitises *Veronica catenata*. Although the two species are morphologically similar, *P. sagittaria* can be distinguished by its smaller conidia (32 × 21 µm on average), longer conidiophores and trunks (405 µm and 243 µm long on average, respectively), as well as narrower trunks and conidiophore bases (11 µm and 8 µm broad on average, respectively).

#### Peronospora arvensis

Taxon classificationFungiPeronosporalesPeronosporaceae

Gäum., Annales Mycologici 16 (1–2): 198. 1918.

A4742090-F3C5-5D07-A564-DB1E3A7050A8

217675

Fig. 4

##### Description.

***Lesions*** on leaves at first chlorotic, later often darkening to become reddish, and diffuse to vein-delimited. ***Down*** present on the lower leaf surface, consisting of scattered to dense felt-like conidiophore outgrowth. ***Conidiophores*** hyaline, straight to curved, thin-walled, 287–406 µm long, av. 347 µm; trunk 168–260 µm long, av. 214 µm, 7–9.5 µm broad, sometimes slightly swollen to up to 13 µm at the base, ratio of the total length to trunk length 1.5–2, callose plugs absent. ***Branching*** subdichotomous in 4–6 orders, with sub-straight to curved branches, gradually attenuate, often at acute angles. ***Ultimate branchlets*** sub-straight to curved, paired branchlets differing in length, with the longer ones 10.5–17.5 µm long, the shorter ones 6.5–11.5 µm long, with a ratio of the longer to the shorter ultimate branchlet of 1.3–1.95, base 2–3 µm broad, apex slightly sharp. ***Conidia*** broadly ellipsoid to ellipsoid with a pale brown to dirty grey colour, 25–28 µm long, 19–21.5 µm broad, length-to-breadth ratio 1.25–1.4, basal part of the conidia mostly protruding. ***Oospores*** 34–41 µm in diameter.

**Figure 4.**
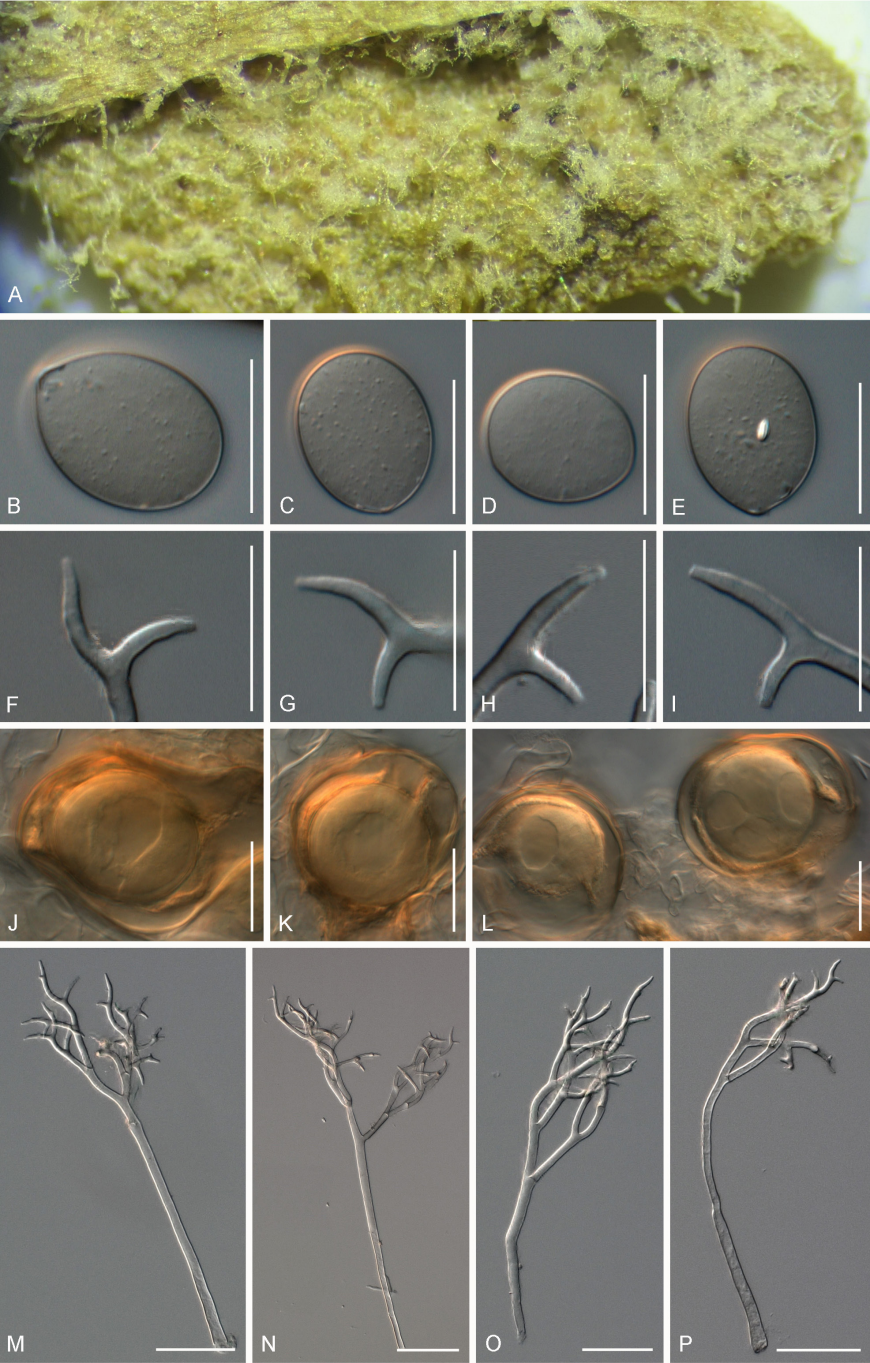
Symptoms and morphology of *Peronospora arvensis* parasitic on *Veronica hederifolia* (GLM-F090770). **A** Down on the leaf surface. **B–E** Conidia. **F–I** Ultimate branchlets. **J–L** Oospores. **M–P** Conidiophores. Scale bars: 20 µm (**B–L**); 50 µm (**M–P**).

##### Type host.

*Veronica hederifolia* (Gäumann 1918).

##### Reported hosts.

Living leaves of *Veronica hederifolia* (Gäumann 1918, 1923; Stevenson 1926; Riethmüller et al. 2002; Göker et al. 2003, 2007), *V. sublobata* (Ellis 2025), *V. triloba* (Voglmayr 2003) and *V. triphyllos* (Gäumann 1918, 1923; Stevenson 1926).

##### Reported distribution.

Germany, France, Switzerland, Austria, Hungary, Denmark, Sweden. Poland, Austria (Gäumann 1923; Stevenson 1926; Riethmüller et al. 2002; Göker et al. 2003, 2007; Voglmayr 2003).

##### Specimens examined

**(all on *Veronica hederifolia*)**. GERMANY. Hessen: Rheingau, Rüdesheim am Rhein, on living leaves of *Veronica hederifolia*, 2 Apr 1916, A. Volkart (ZT81629); Saxony: Görlitz-Rauschwalde, 24 Apr 2005, S. Hoeflich (GLM-F062270); Kunnersdorf, 7 May 2008, H. Boyle (GLM-F090770); Leipzig, 17 May 1985, H. Jage (GLM-F069391); Leipzig, 11 Mar 1990, H. Jage (GLM-F079120); Lodenau, 29 Apr 2008, H. Boyle (GLM-F081506); Podrosche, 24 Apr 2008, H. Boyle (GLM-F081427); Prositz, 1 May 1998, H. Jage (GLM-F073863); Thümmlitz, 16 Apr 1993, H. Jage (GLM-F068053); Saxony-Anhalt: Dessau, 24 Apr 2002, H. Jage (GLM-F076685); Field between Wallwitz and the Petersberg, 5 May 1878, Oertel (ZT81621); Gänsefurth, 27 Mar 2004, H. Jage (GLM-F063010); Gänsefurth, 12 Mar 2004, H. Jage (GLM-F063062); Hohenkirchen, 14 Apr 2001, H. Jage (GLM-F074167); Klein Wanzleben, 4 May 2001, H. Jage (GLM-F074159); Klitzschena, 29 Apr 1992, H. Jage (GLM-F067424); Könnern, 4 May 2003, H. Jage (GLM-F073461); Meuro, 17 May 1986, H. Jage (GLM-F068191); Roßla, 20 Apr 2005, H. Jage (GLM-F075761); Schadeleben, Cemetery, 22 Apr 2001, H. Jage (GLM-F074206); Seeburg, 1 May 2001, H. Jage (GLM-F074186); Wittgendorf, 14 Apr 2001, H. Jage (GLM-F074174).

##### Notes.

*Peronospora arvensis* is widely distributed in Europe and has been reported from *Veronica hederifolia*, its type host, *V. sublobata* (Ellis 2025), *V. triloba*, and *V. triphyllos* (Gäumann 1918, 1923; Stevenson 1926; Riethmüller et al. 2002; Göker et al. 2003, 2007; Voglmayr 2003; this study). These host species are very difficult to distinguish, and identification errors are common. In fact, many of the specimens included in this study were initially misidentified as *V. sublobata*. However, they only had two nucleotide differences compared to the ITS sequences of *Veronica hederifolia* in GenBank, and based on the host DNA barcode (*matK*), they are identical to *Veronica hederifolia*. Based on our phylogenetic analyses, the *Peronospora* species parasitising *Veronica triphyllos* represents a distinct previously undescribed taxon, and *P. arvensis* is largely confined to its type host, *Veronica hederifolia*. One *P. arvensis* specimen, WU-MYC0022883, collected from Austria in 1999, was recorded infecting *Veronica triloba* (Voglmayr 2003). However, based on the host ITS and *matK* sequences, two specimens (WU-MYC0032791 and WU-MYC0032807) from *Veronica triloba* actually represent a previously undescribed species distinct from *P. arvensis*, therefore, the host of specimen WU-MYC0022883, identified as *Veronica triloba*, is likely a misidentification. This highlights the importance of DNA molecular identification for hosts that are not easily identified, as it is crucial for determining the species specificity of *Peronospora*. Phylogenetic analyses indicate that *P. arvensis* is closely related to the *Peronospora* sp. on *Veronica triloba* and *P. veronicae-cymbalariae* on *Veronica cymbalaria*. Due to the lack of specimens of *Peronospora* sp. on *Veronica triloba*, this new species has not been described in this study. Despite this, when compared with *P. arvensis*, *P. veronicae-cymbalariae* still had larger conidia, 32 × 23 µm on average, and shorter conidiophores and trunks, 246 µm and 117 µm in breadth on average, respectively.

#### Peronospora brevibrachia

Taxon classificationFungiPeronosporalesPeronosporaceae

M. Mu & Thines sp. nov.

DDDE76A5-246A-5242-B30A-539CE5627A7A

860227

Fig. 5

##### Etymology.

The Latin word “brevis” means short, and “brachium” means branch, referring to the shortest branches of this species.

**Figure 5.**
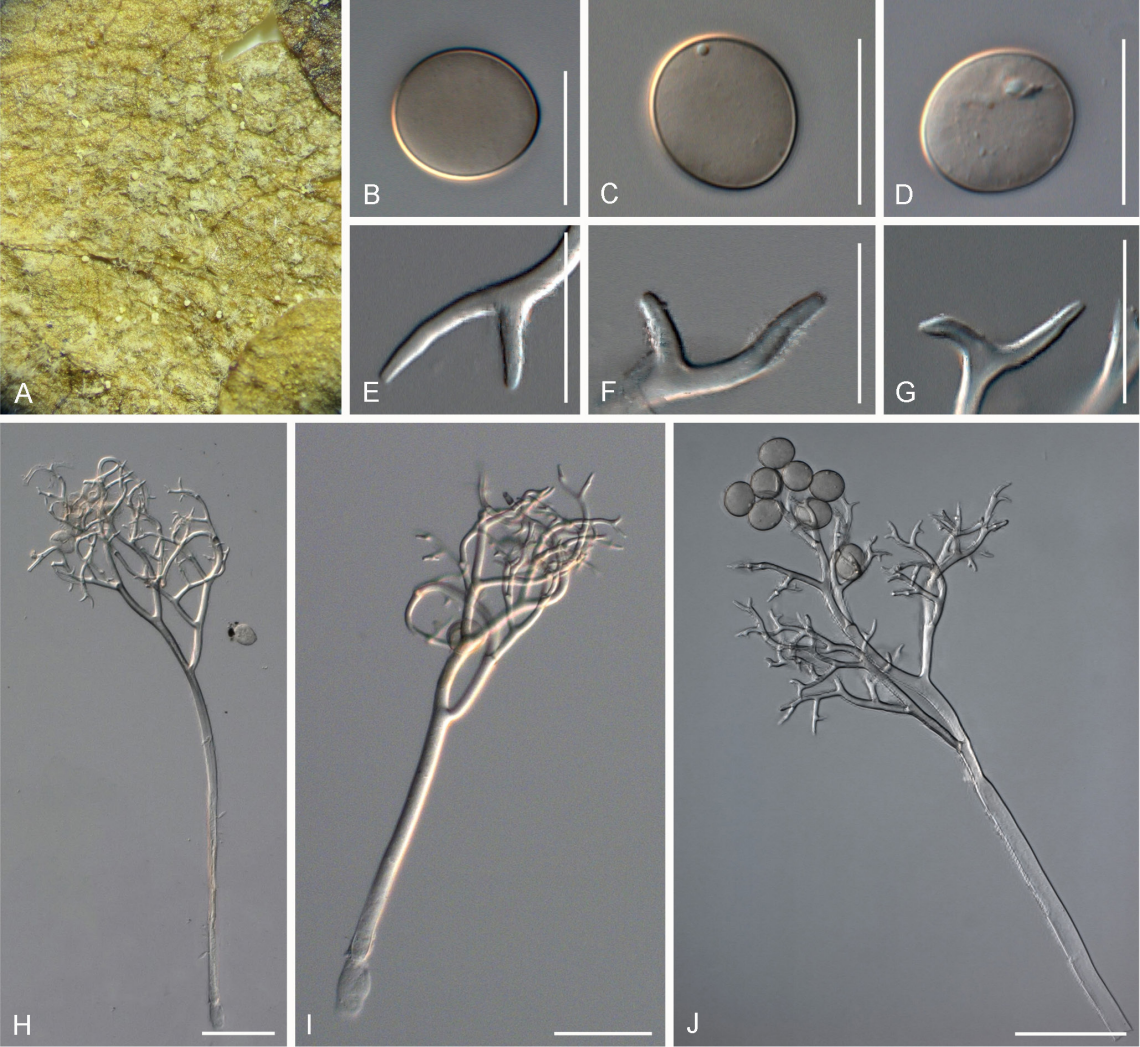
Symptoms and morphology of *Peronospora brevibrachia* parasitic on *Veronica chamaedrys* (GLM-F074111). **A** Down on the leaf surface. **B–D** Conidia. **E–G** Ultimate branchlets. **H–J** Conidiophores. Scale bars: 20 µm (**B–G**); 50 µm (**H–J**).

##### Diagnosis.

Differs from *P. microspora* in having larger conidia, longer conidiophores and trunks, as well as shorter ultimate branchlets. Differs from *P. petricosa* in having more elongated conidia, as well as longer conidiophores and trunks.

##### Type.

GERMANY. Saxony-Anhalt: Hohes Holz, on living leaves of *Veronica chamaedrys*, 9 May 1998, H. Jage (holotype GLM-F074111).

##### Description.

***Lesions*** on leaves initially yellow-green or light brown, then reddish brown or dark brown upon drying, diffuse to vein-delimited. ***Down*** present on the lower leaf surface, grey-violet, consisting of scattered to dense felt-like conidiophore outgrowth. ***Conidiophores*** hyaline, straight to curved, thin-walled, 313–447 µm long, av. 380 µm; trunk 177–304 µm long, av. 240 µm, 5.5–8.5 µm broad, sometimes slightly swollen to up to 12 µm at the base, ratio of the total length to trunk length 1.5–2, callose plugs absent. ***Branching*** subdichotomous in 4–8 orders, with sub-straight to curved branches, gradually attenuate, often at acute angles. ***Ultimate branchlets*** sub-straight to curved, paired branchlets differing in length, with the longer ones 8–12 µm long, the shorter ones 5–9 µm long, with a ratio of the longer to the shorter ultimate branchlet of 1.2–1.75, base 1.5–2 µm broad, apex slightly sharp. ***Conidia*** broadly ellipsoidal to ellipsoidal with a yellow-brown colour, 20.5–23 µm long, 15–17 µm broad, length-to-breadth ratio 1.25–1.5. ***Oospores*** not observed.

##### Habitat.

Living leaves of *Veronica chamaedrys*.

##### Distribution.

Austria, Germany.

##### Additional specimen examined.

GERMANY. Saxony-Anhalt: Uthausen, Mark Naundorf, on living leaves of *Veronica chamaedrys*, 12 Oct 1998, H. Jage (GLM-F068222).

##### Notes.

*Peronospora brevibrachia* is a parasite of *Veronica chamaedrys*. Another specimen, WU-MYC0022873, also parasitic on *Veronica chamaedrys*, has been identified as *P. agrestis* (Voglmayr 2003), based on the assumption of a broader host range for that species. However, in the ITS phylogenetic analyses, WU-MYC0022873 on *Veronica chamaedrys* forms a subclade together with *P. brevibrachia* on *Veronica chamaedrys* (Additional file 1). Previous research suggests that close relationships among *Peronospora* species are often associated with morphological similarities (Mu et al. 2024a, b). In line with this, *Peronospora brevibrachia*, *P. microspora*, and *P. petricosa* form a well-supported clade, all characterised by more globose conidia, with average length-to-breadth ratios of 1.4, 1.25, and 1.3, respectively. However, *P. brevibrachia* differs by having longer conidiophores and trunks, averaging 380 µm and 240 µm, respectively, as compared to *P. microspora* and *P. petricosa*.

#### Peronospora chionodendron

Taxon classificationFungiPeronosporalesPeronosporaceae

M. Mu & Thines sp. nov.

30241389-2DD5-5CAB-A81F-3505D22D8055

860231

Fig. 6

##### Etymology.

The specific epithet “chionodendron” is derived from the Greek chion (“snow”) and *-dendron* (“tree”) as a noun in apposition, referring to the snow-covered, tree-like appearance of the species as observed macroscopically, reminiscent of a snow-laden winter tree.

**Figure 6.**
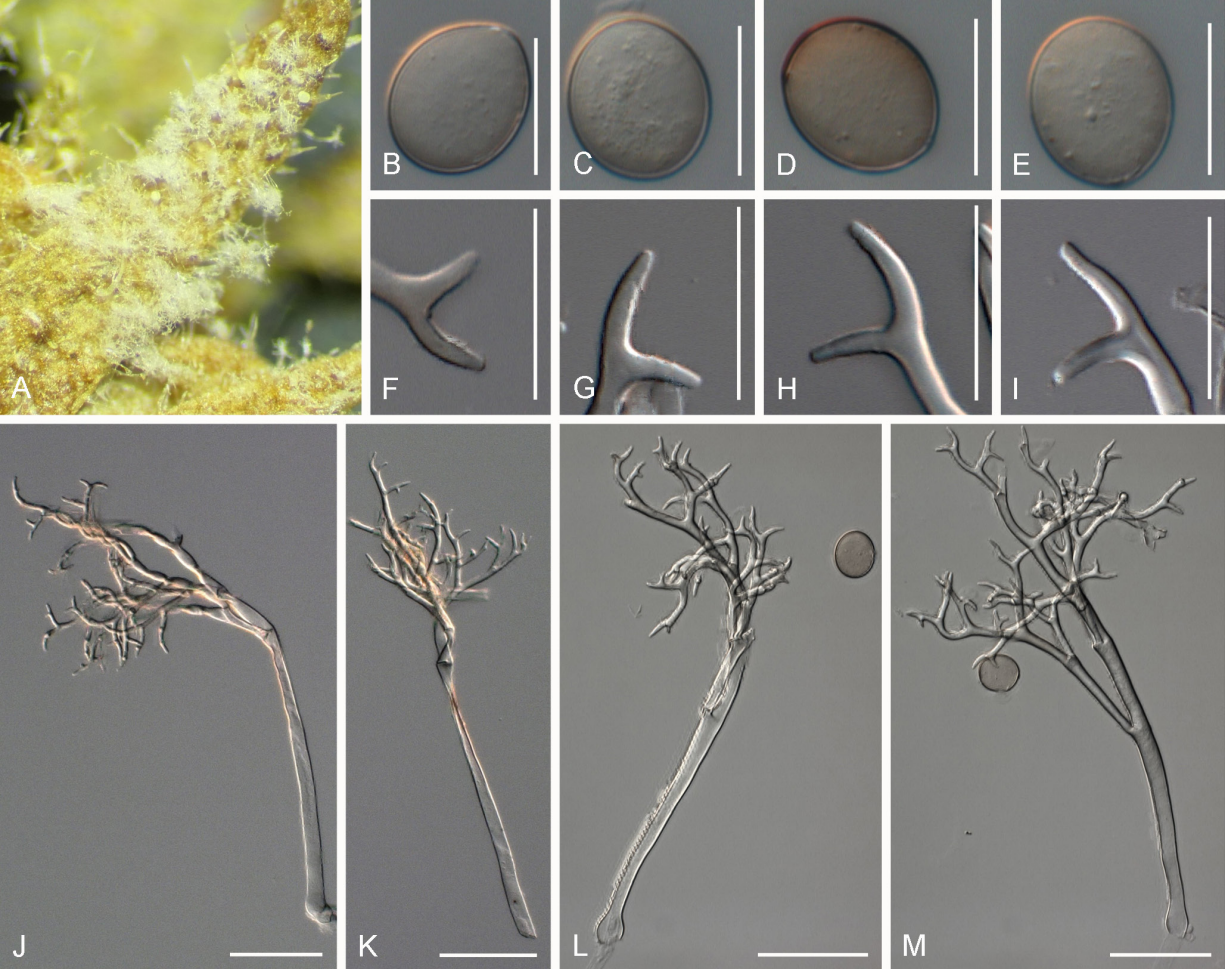
Symptoms and morphology of *Peronospora chionodendron* parasitic on *Veronica verna* (GLM-F076884). **A** Down on the leaf surface. **B–E** Conidia. **F–I** Ultimate branchlets. **J–M** Conidiophores. Scale bars: 20 µm (**B–I**); 50 µm (**J–M**).

##### Diagnosis.

Differs from *P. brevibrachia* in having broader and more globose conidia, shorter conidiophores and trunks, broader trunks, as well as longer ultimate branchlets. Differs from *P. microspora* in having larger conidia and broader trunks. Differs from *P. petricosa* in having larger conidia and broader trunks.

##### Type.

GERMANY. Saxony-Anhalt: Mücheln, on living leaves of *Veronica verna*, 26 May 2000, H. Jage (holotype GLM-F076884).

##### Description.

***Lesions*** on leaves dark brown to violet, diffuse to vein-delimited. ***Down*** present on the lower leaf surface, consisting of scattered to dense felt-like conidiophore outgrowth. ***Conidiophores*** hyaline, straight to curved, thin-walled, 255–369 µm long, av. 312 µm; trunk 137–216 µm long, av. 176 µm, 6.5–10 µm broad, sometimes slightly swollen to up to 12 µm at the base, ratio of the total length to trunk length 1.5–2, callose plugs absent. ***Branching*** subdichotomous in 4–7 orders, with sub-straight to curved branches, gradually attenuate, often at acute angles. ***Ultimate branchlets*** sub-straight to curved, paired branchlets differing in length, with the longer ones 8.5–14 µm long, the shorter ones 5.5–9.5 µm long, with a ratio of the longer to the shorter ultimate branchlet of 1.25–1.7, base 2–2.5 µm broad, apex slightly sharp. ***Conidia*** subglobose to broadly ellipsoidal with a pale grey to pale violet colour, 19.5–23 µm long, 15.5–18.5 µm broad, length-to-breadth ratio 1.15–1.35. ***Oospores*** not observed.

##### Habitat.

Living leaves of *Veronica verna*.

##### Distribution.

Germany.

##### Additional specimens examined.

GERMANY. Saxony-Anhalt: Bülzig, on living leaves of *Veronica verna*, 28 Apr 2004, H. Jage (GLM-F064157); Kemberg, on living leaves of *Veronica verna*, 18 May 2002, H. Jage (GLM-F076762); Ritzleben, Riebau, on living leaves of *Veronica verna*, 24 Apr 2004, H. Jage (GLM-F064162); Wahlitz, on living leaves of *Veronica verna*, 15 May 2003, H. Jage (GLM-F078081).

##### Notes.

It is noteworthy that *Veronica verna* from North America, and one ITS sequence (MG220171) of this species collected in Canada, show identical ITS sequences as our samples collected in Germany (see also Kuzmina et al. 2017). Although *Peronospora grisea* and *Peronospora verna* have been reported to infect *Veronica verna* (Gäumann 1923), the *Peronospora* species on *Veronica verna* appears to represent a previously undescribed species, named *Peronospora chionodendron* in this study. In terms of morphology, *P. chionodendron* had the roundest conidia compared to all *Peronospora* species on *Veronica*, with a length-to-breadth ratio of 1.25 ± 0.09.

#### Peronospora fidelia

Taxon classificationFungiPeronosporalesPeronosporaceae

M. Mu & Thines sp. nov.

074A9BE1-E26C-5C66-B380-A7B5DF551728

860228

Fig. 7

##### Etymology.

*Veronica* symbolizes loyalty, the epithet is derived from Latin “fides” (loyal), which refers to the symbolism of flowers.

**Figure 7.**
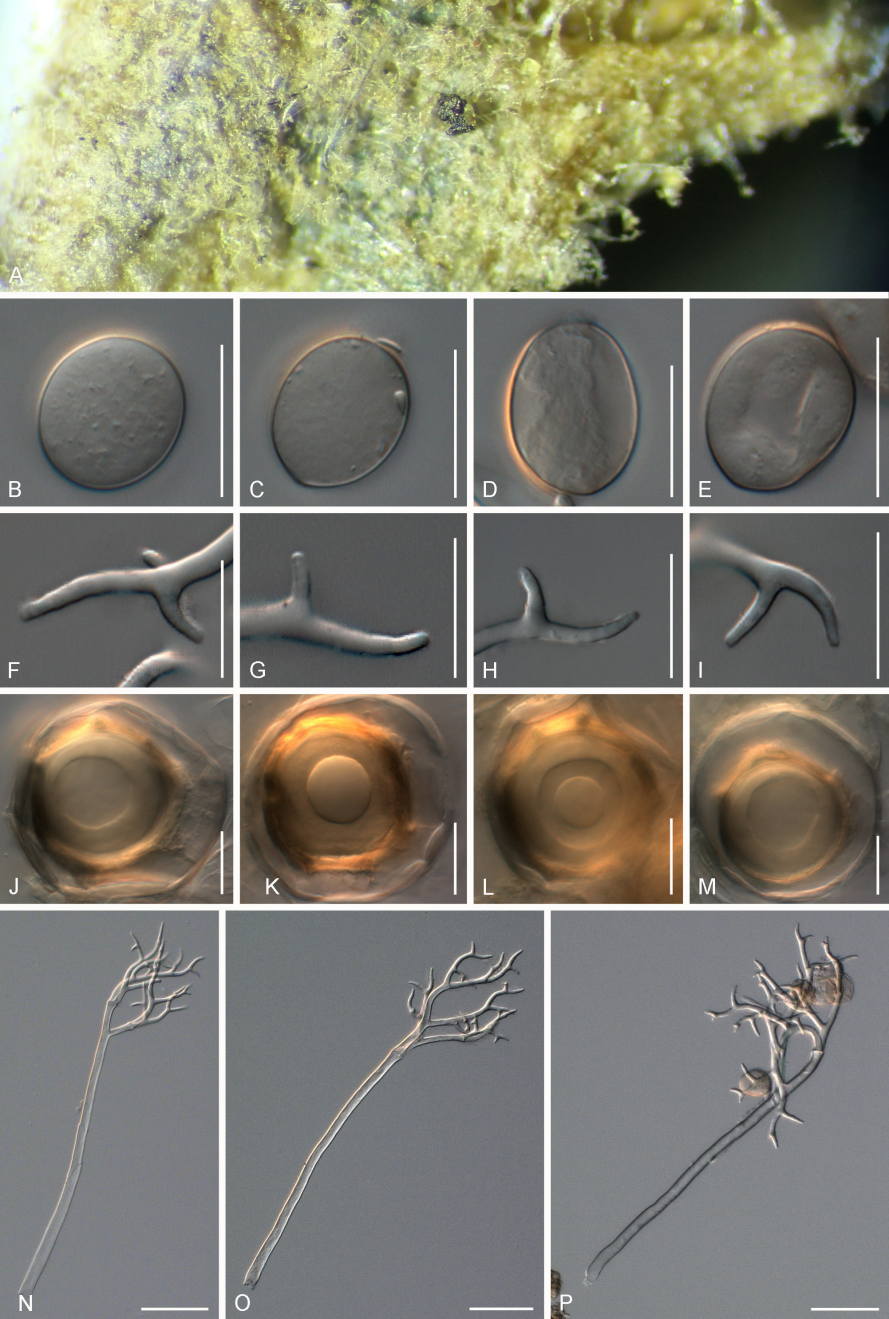
Symptoms and morphology of *Peronospora fidelia* parasitic on *Veronica triphyllos* (GLM-F063005). **A** Down on the leaf surface. **B–E** Conidia. **F–I** Ultimate branchlets. **J–M** Oospores. **N–P** Conidiophores. Scale bars: 20 µm (**B–M**); 50 µm (**N–P**).

##### Diagnosis.

Differs from *P. agrestis* in having larger conidia, as well as shorter conidiophores and trunks. Differs from *P. gracilis* in having larger and more elongated conidia, as well as longer ultimate branchlets.

##### Type.

GERMANY. Saxony-Anhalt: Annaburg southwest, on living leaves of *Veronica triphyllos*, 1 May 2004, H. Jage (holotype GLM-F063005).

##### Description.

***Lesions*** on leaves at first pale green, later expanding to dark brown, diffuse to vein-delimited. ***Down*** present on the lower leaf surface, consisting of scattered to dense felt-like conidiophore outgrowth. ***Conidiophores*** hyaline, straight to curved, thin-walled, 238–352 µm long, av. 295 µm; trunk 123–220 µm long, av. 171 µm, 7–8.5 µm broad, sometimes slightly swollen to up to 14 µm at the base, ratio of the total length to trunk length 1.5–2, callose plugs absent. ***Branching*** subdichotomous in 4–6 orders, with sub-straight to curved branches, gradually attenuate, often at right angles. ***Ultimate branchlets*** sub-straight to curved, paired branchlets differing in length, with the longer ones 10–15.5 µm long, the shorter ones 6.5–10 µm long, with a ratio of the longer to the shorter ultimate branchlet of 1.3–1.85, base 2.5–3 µm broad, apex slightly sharp. ***Conidia*** ellipsoid with a grey to reddish colour, 20–25 µm long, 15–18.5 µm broad, length-to-breadth ratio 1.2–1.5. ***Oospores*** 36–44 µm in diameter.

##### Habitat.

Living leaves of *Veronica triphyllos*.

##### Distribution.

Austria, Germany.

##### Additional specimens examined.

AUSTRIA. Burgenland: District of Neusiedl am See, Municipality of Breitenbrunn, Tenauriegel, Weingarten, on living leaves of *Veronica triphyllos*, 21 Apr 2013, H. Voglmayr (WU-MYC0032815); District of Neusiedl am See, Municipality of Winden, Hackelsberg S Jois, Annuellesflur at the summit, on living leaves of *Veronica triphyllos*, 13 Apr 2012, H. Voglmayr (WU-MYC0032805); Lower Austria: District of Bruck/Leitha, Municipality of Prellenkirchen, Spitzerberg, S-Slope S Edelstal, Weingarten, on living leaves of *Veronica triphyllos*, 14 Apr 2012, H. Voglmayr (WU-MYC0032806); District of Hollabrunn, Municipality of Retz, Oberalb, Roßberg, Neubergen, Weingarten, on living leaves of *Veronica triphyllos*, 27 Apr 2011, H. Voglmayr (WU-MYC0032801); District of Tulln, Municipality of Fels am Wagram, Dorner S Gösing am Wagram, vineyards, on living leaves of *Veronica triphyllos*, 17 Apr 2012, H. Voglmayr (WU-MYC0032808).

##### Notes.

*Peronospora fidelia* and *P. gracilis* differ only by two gaps in the ITS region and a single base difference in the LSU D1–3 region, with no variation in the LSU D6–8 region. However, significant and numerous base differences are observed in the four mitochondrial loci (*COX1*, *COX2*, *NAD1*, and *RPS10*). A similar pattern is found between *Peronospora* sp. on *Veronica triloba* and *P. veronicae-cymbalariae* on *Veronica cymbalaria* in this study. In both cases, nuclear loci do not offer sufficient resolution to effectively distinguish these species. This phenomenon has also been reported in previous studies (Choi et al. 2015b), particularly in LSU regions, but is rarely seen in ITS. These findings highlight the importance of multi-locus approaches for accurate species identification (Choi et al. 2015b). In the mitochondrial loci phylogenies, *P. fidelia* and *P. gracilis* are confirmed as sister species. Despite their close relationship and morphological similarities, *P. gracilis* differs from *P. fidelia* by having smaller and more globose conidia, 21 × 16.5 µm on average, with a length-to-breadth ratio of 1.25 ± 0.1, and shorter ultimate branchlets, the longer branchlets averaging 11 µm and the shorter ones 7.5 µm. *Peronospora fidelia* is also closely related to *P. agrestis*, but *P. agrestis* has smaller conidia, 21 × 16 µm on average, as well as longer conidiophores and trunks, averaging 334 µm and 200 µm, respectively.

#### Peronospora gracilis

Taxon classificationFungiPeronosporalesPeronosporaceae

M. Mu & Thines sp. nov.

B16FFD51-4FE6-5518-A99B-1892E9929D34

860229

Fig. 8

##### Etymology.

“Gracilis” meaning slender or small, refers to the slenderness and small size of the host plants infected with the species.

**Figure 8.**
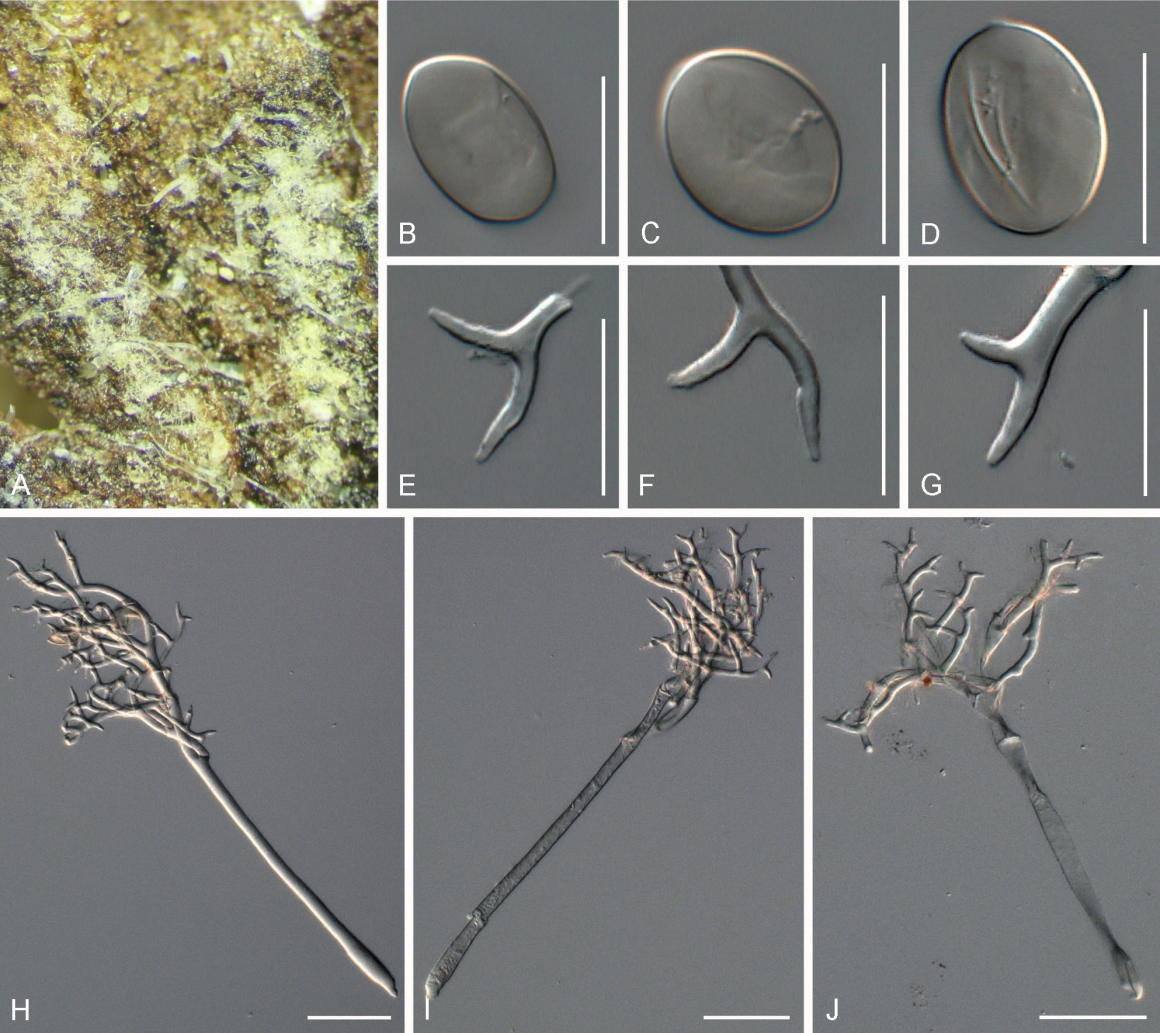
Symptoms and morphology of *Peronospora gracilis* parasitic on *Veronica praecox* (GLM-F069988). **A** Down on the leaf surface. **B–D** Conidia. **E–G** Ultimate branchlets. **H–J** Conidiophores. Scale bars: 20 µm (**B–G**); 50 µm (**H–J**).

##### Diagnosis.

Differs from *P. agrestis* in having shorter conidiophores and trunks, as well as shorter ultimate branchlets. Differs from *P. fidelia* in having smaller and more globose conidia, as well as shorter ultimate branchlets.

##### Type.

GERMANY. Saxony-Anhalt: Lieskau, on living leaves of *Veronica praecox*, 18 Apr 1990, H. Jage (holotype GLM-F069988).

##### Description.

***Lesions*** on leaves purplish later often darkening to become brownish, often vein-delimited. ***Down*** present on the lower leaf surface, consisting of scattered to dense felt-like conidiophore outgrowth. ***Conidiophores*** hyaline, straight to curved, thin-walled, 266–329 µm long, av. 298 µm; trunk 139–205 µm long, av. 172 µm, 5–8.5 µm broad, sometimes slightly swollen to up to 16 µm at the base, ratio of the total length to trunk length 1.5–2, callose plugs absent. ***Branching*** subdichotomous in 4–6 orders, with sub-straight to curved branches, gradually attenuate, often at right angles. ***Ultimate branchlets*** sub-straight to curved, paired branchlets differing in length, with the longer ones 8.5–13.5 µm long, the shorter ones 6–9 µm long, with a ratio of the longer to the shorter ultimate branchlet of 1.25–1.75, base 2–3 µm broad, apex slightly sharp. ***Conidia*** subglobose to broadly ellipsoid with a grey colour, 19–23.5 µm long, 15.5–18 µm broad, length-to-breadth ratio 1.15–1.4, basal part of the conidia mostly protruding. ***Oospores*** not observed.

##### Habitat.

Living leaves of *Veronica praecox*.

##### Distribution.

Germany.

##### Additional specimens examined.

GERMANY. Saxony-Anhalt: Seeburg, on living leaves of *Veronica praecox*, 1 May 2001, H. Jage (GLM-F074185).

##### Notes.

*Peronospora gracilis* has two closely related sister species, *P. fidelia* and *P. agrestis*. However, *P. gracilis* is distinguished by its shorter ultimate branchlets, with the longer branchlets averaging 11 µm and the shorter ones averaging 7.5 µm.

#### Peronospora grisea

Taxon classificationFungiPeronosporalesPeronosporaceae

Unger, Bot. Ztg. 5 (18): 315. (1847).

522CD6EC-FC65-50A9-885A-897E6900DAA3

225682

Fig. 9

≡ Botrytis grisea (Link) Fr., Systema Mycologicum 3: 396. 1832.
= Peronospora verna Gäum., Annls mycol. 16 (1/2): 198. 1918.

##### Description.

***Lesions*** on leaves greyish, reddening to dark brown when dried, often vein-delimited. ***Down*** present on the lower leaf surface, greyish-white, consisting of scattered to dense felt-like conidiophore outgrowth. ***Conidiophores*** hyaline, straight to curved, thin-walled, 250–335 µm long, av. 293 µm; trunk 122–207 µm long, av. 165 µm, 6.5–8 µm broad, sometimes slightly swollen to up to 13 µm at the base, ratio of the total length to trunk length 1.5–2, callose plugs absent. ***Branching*** subdichotomous in 4–7 orders, with sub-straight to curved branches, gradually attenuate, often at right angles. ***Ultimate branchlets*** sub-straight to curved, paired branchlets differing in length, with the longer ones 9–14 µm long, the shorter ones 6–10 µm long, with a ratio of the longer to the shorter ultimate branchlet of 1.2–1.65, base 2–2.5 µm broad, apex slightly sharp. ***Conidia*** ovate to ellipsoid with a brown colour, 20–25 µm long, 15–18 µm broad, length-to-breadth ratio 1.25–1.5, basal part of the conidia mostly protruding. ***Oospores*** not observed.

**Figure 9.**
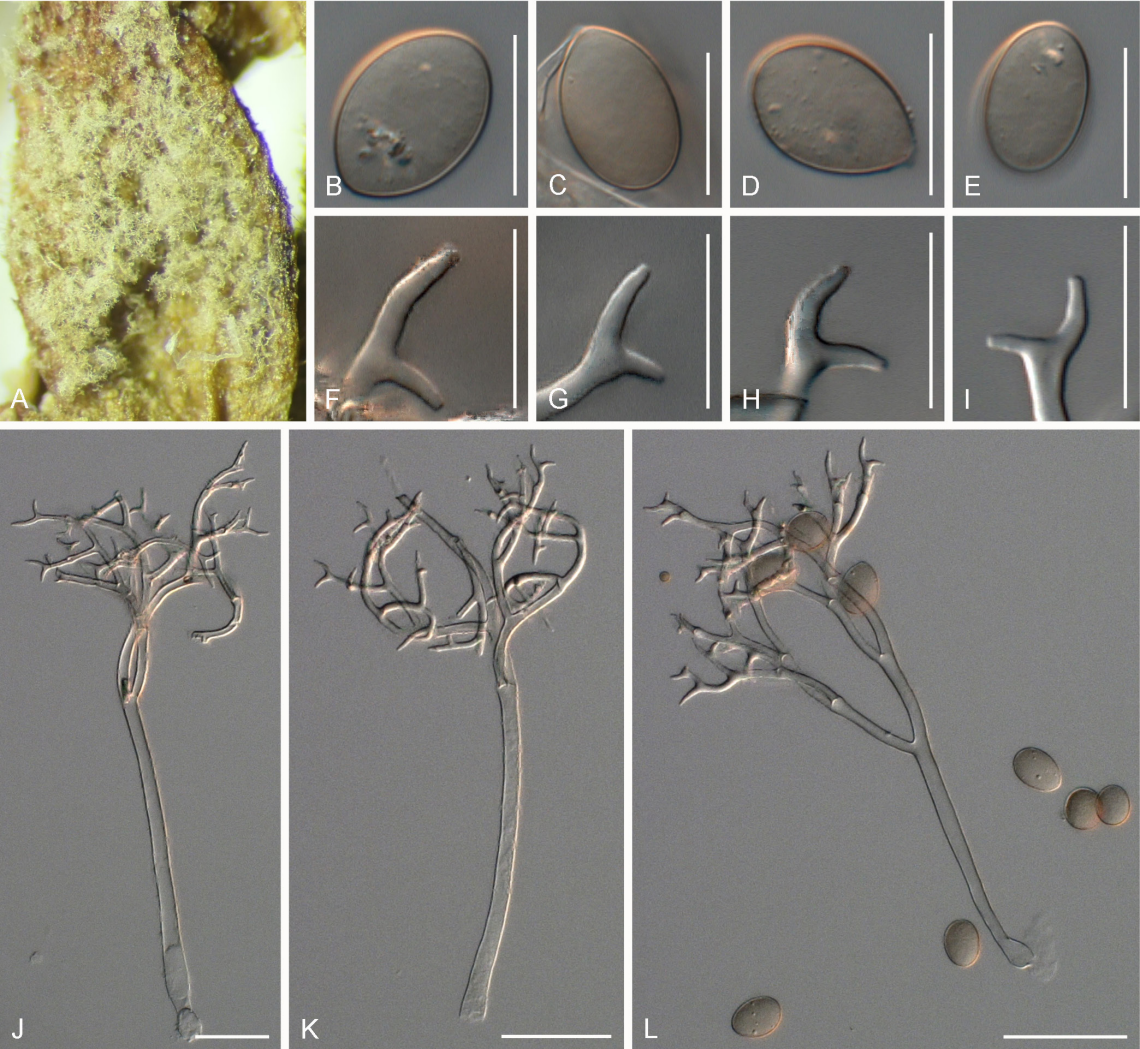
Symptoms and morphology of *Peronospora grisea* parasitic on *Veronica beccabunga* (GLM-F075648). **A** Down on the leaf surface. **B–E** Conidia. **F–I** Ultimate branchlets. **J–L** Conidiophores. Scale bars: 20 µm (**B–I**); 50 µm (**J–L**).

##### Type host.

*Veronica beccabunga* (Unger 1847).

##### Confirmed additional host.

*Veronica serpyllifolia*.

##### Reported hosts.

*Veronica beccabunga* (Unger 1847; Gäumann 1923; Stevenson 1926; Riethmüller et al. 2002), *V. arvensis*, *V. hederifolia*, *V. verna* (de Bary 1863), *V. peregrina* (Swingle 1887), *Veronica serpyllifolia* (de Bary 1863; Voglmayr 2003).

##### Reported distribution.

Germany (de Bary 1863; Stevenson 1926; Riethmüller et al. 2002), USA (Swingle 1887), Austria, Croatia, Denmark, France, Hungary, Ireland, Netherlands, Norway Russia, Sweden, Switzerland, UK (Fries 1832; Gäumann 1923; Voglmayr 2003).

##### Specimens examined.

GERMANY. Saxony-Anhalt: Bergwitz, on living leaves of *Veronica serpyllifolia*, 16 Apr 2002, H. Jage (GLM-F076812); Bülzig, on living leaves of *Veronica beccabunga*, 28 Apr 2004, H. Jage (GLM-F064183); Hayn, on living leaves of *Veronica serpyllifolia*, 20 Apr 2005, H. Jage (GLM-F075766); Lodersleben, on living leaves of *Veronica beccabunga*, 9 Apr 2004, H. Jage (GLM-F063053); Lützen-Hohenmölsener Platte, Gieckau east, Nautschke Creek, on living leaves of *Veronica beccabunga*, 14 Jun 2005, H. Richter (GLM-F075648); Morungen, on living leaves of *Veronica beccabunga*, 21 Apr 2005, A. Hoch (GLM-F075774); Neckendorf, Helme, on living leaves of *Veronica beccabunga*, 1 Sep 2002, H. Jage (GLM-F076527); Obhausen, on living leaves of *Veronica beccabunga*, 3 May 2005, H. Jage (GLM-F075736); Straßberg, on living leaves of *Veronica beccabunga*, 10 Sep 2005, H. Jage (GLM-F075832); Wörlitz, Auwiese, on living leaves of *Veronica serpyllifolia*, 20 May 2002, H. Jage (GLM-F078065); Saxony: Torgau, on living leaves of *Veronica serpyllifolia*, 3 May 2002, H. Jage (GLM-F076783); SWEDEN. Uppsala: Uppsala-Norby, on living leaves of *Veronica serpyllifolia*, 16 May 2007, H. Boyle (GLM-F079274).

##### Notes.

It is surprising that *P. grisea* on *Veronica beccabunga* (the type host of *P. grisea*) and *P. verna* on *Veronica serpyllifolia* (the type host of *P. verna*) are genetically identical in LSU D1-3, LSU D6-8, *COX*1, *COX*2, and *RPS10*, with only a single base difference in the ITS and *NAD1* regions. Given the principle of priority by chronological order, *P. verna* is relegated to synonymy with *P. grisea* here. However, it would be meaningful to investigate the relationships between the populations of both hosts to determine how far they are genetically separated enough to be considered independent subspecies. In any case, the pathogens on these two hosts might provide a model for studying speciation processes in downy mildews in particular, and obligate biotrophic pathogens in general. Furthermore, this finding emphasises the limitations of relying solely on host-specificity for species identification of the pathogens. Although in most cases *Peronospora* species are highly host-specific, there are some exceptions due to recent host jumping (Thines 2019; Ploch et al. 2022) or multiple colonisations of the same host species (Mu et al. 2024a). One specimen, WU-MYC0022901, parasitic on *Veronica serpyllifolia*, was also identified as *P. grisea* in Voglmayr’s 2003 study, based on ITS sequences (AY198241), although *Veronica serpyllifolia* was the type host for *P. verna* (Gäumann 1918). The differences observed by Gäumann can likely be attributed to the effect of the host matrix, as it has been previously shown in *Pseudoperonospora cubensis*, where the host matrix can influence several morphological characteristics of the pathogen significantly (Runge et al. 2012). Morphologically, *P. grisea* is characterised by its rather short conidiophores and trunks, with average lengths of 293 µm and 165 µm, respectively.

#### Peronospora microspora

Taxon classificationFungiPeronosporalesPeronosporaceae

M. Mu & Thines sp. nov.

2A83EC2A-B1E9-536F-ACEE-B663DE5CD0BA

860230

Fig. 10

##### Etymology.

“Microspora” refers to the small conidia of this species.

**Figure 10.**
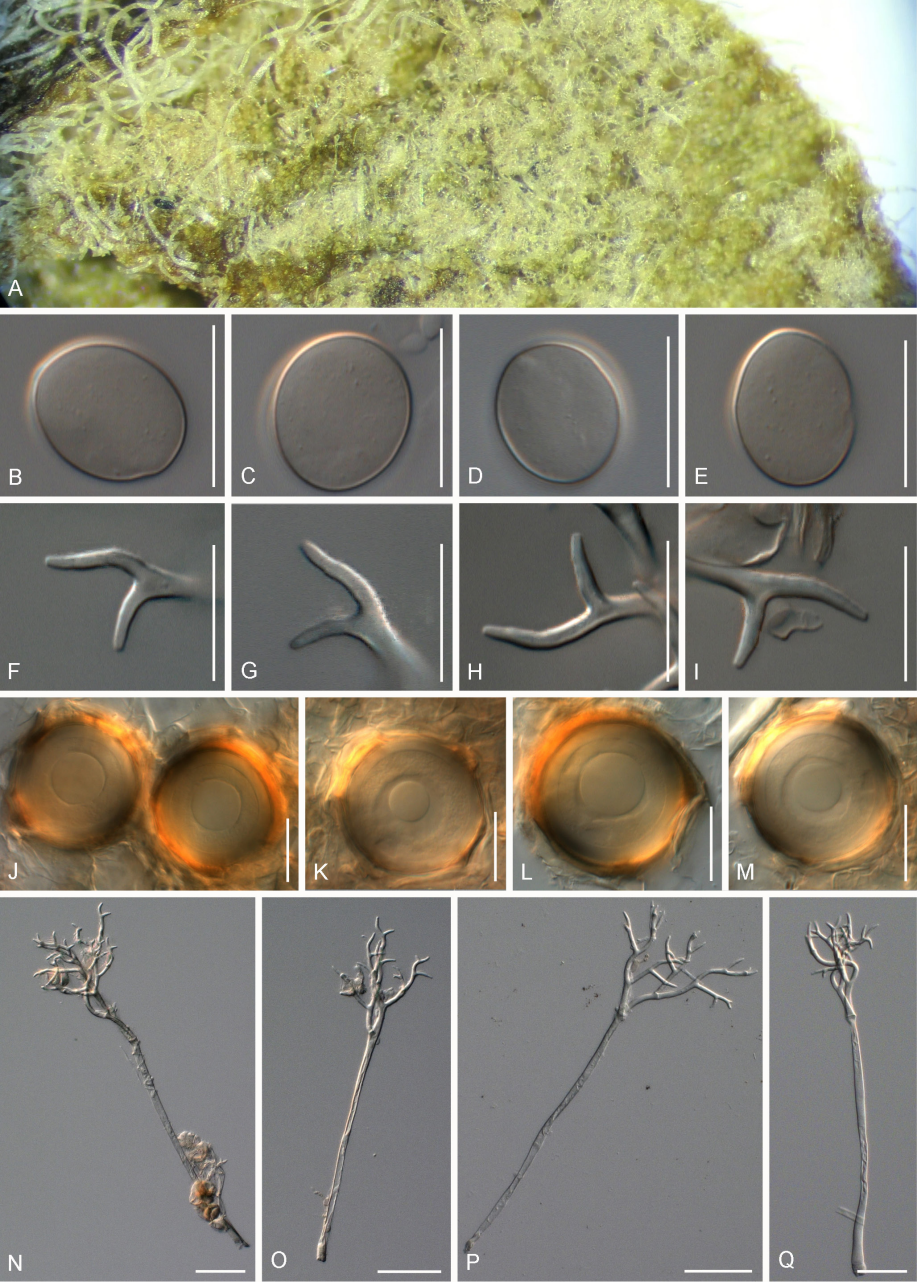
Symptoms and morphology of *Peronospora microspora* parasitic on *Veronica teucrium* (GLM-F073861). **A** Down on the leaf surface. **B–E** Conidia. **F–I** Ultimate branchlets. **J–M** Oospores. **N–Q** Conidiophores. Scale bars: 20 µm (**B–M**); 50 µm (**N–Q**).

##### Diagnosis.

Differs from *P. brevibrachia* in having smaller conidia, shorter conidiophores and trunks, as well as longer ultimate branchlets. Differs from *P. petricosa* in having smaller conidia.

##### Type.

GERMANY. Saxony: Prositz, on living leaves of *Veronica teucrium*, 1 May 1998, H. Jage (holotype GLM-F073861).

##### Description.

***Lesions*** on leaves chlorotic, then turning dark yellow to brown when dried, diffuse to vein-delimited. ***Down*** present on the lower leaf surface, consisting of scattered to dense felt-like conidiophore outgrowth. ***Conidiophores*** hyaline, straight to curved, thin-walled, 245–346 µm long, av. 295 µm; trunk 135–223 µm long, av. 179 µm, 5.3–7.7 µm broad, sometimes slightly swollen to up to 12 µm at the base, ratio of the total length to trunk length 1.4–2, callose plugs absent. ***Branching*** subdichotomous in 4–6 orders, with sub-straight to curved branches, gradually attenuate, often at right angles. ***Ultimate branchlets*** sub-straight to curved, paired branchlets differing in length, with the longer ones 9–14.5 µm long, the shorter ones 6–10 µm long, with a ratio of the longer to the shorter ultimate branchlet of 1.2–1.8, base 2–3 µm broad, apex slightly sharp. ***Conidia*** subglobose to broadly ellipsoidal with a grey to pale reddish colour, 17–21.5 µm long, 13.5–17 µm broad, length-to-breadth ratio 1.15–1.4. ***Oospores*** 35–49 µm in diameter.

##### Habitat.

Living leaves of *Veronica teucrium*.

##### Distribution.

Germany.

##### Additional specimens examined.

GERMANY. Saxony: Prositz, on living leaves of *Veronica teucrium*, 6 Jun 1999, H. Jage (GLM-F078282); Saxony-Anhalt: Eilenstedt, on living leaves of *Veronica teucrium*, 2 Jul 2002, H. Jage (GLM-F076577); Eilenstedt, on living leaves of *Veronica teucrium*, 13 May 1999, H. Jage (GLM-F077783).

##### Notes.

*Peronospora microspora*, *P. brevibrachia*, and *P. petricosa* form a strongly supported clade. However, *P. microspora* differs by having smaller conidia, averaging 19.5 µm in length and 15.5 µm in breadth.

#### Peronospora obscura

Taxon classificationFungiPeronosporalesPeronosporaceae

M. Mu & Thines sp. nov.

29254060-FF24-53D2-87BD-240F1E26DDFA

860232

Fig. 11

##### Etymology.

The epithet refers to the species being concealed from discovery due to the erroneous assumption of a broader host range for *P. silvestris*.

**Figure 11.**
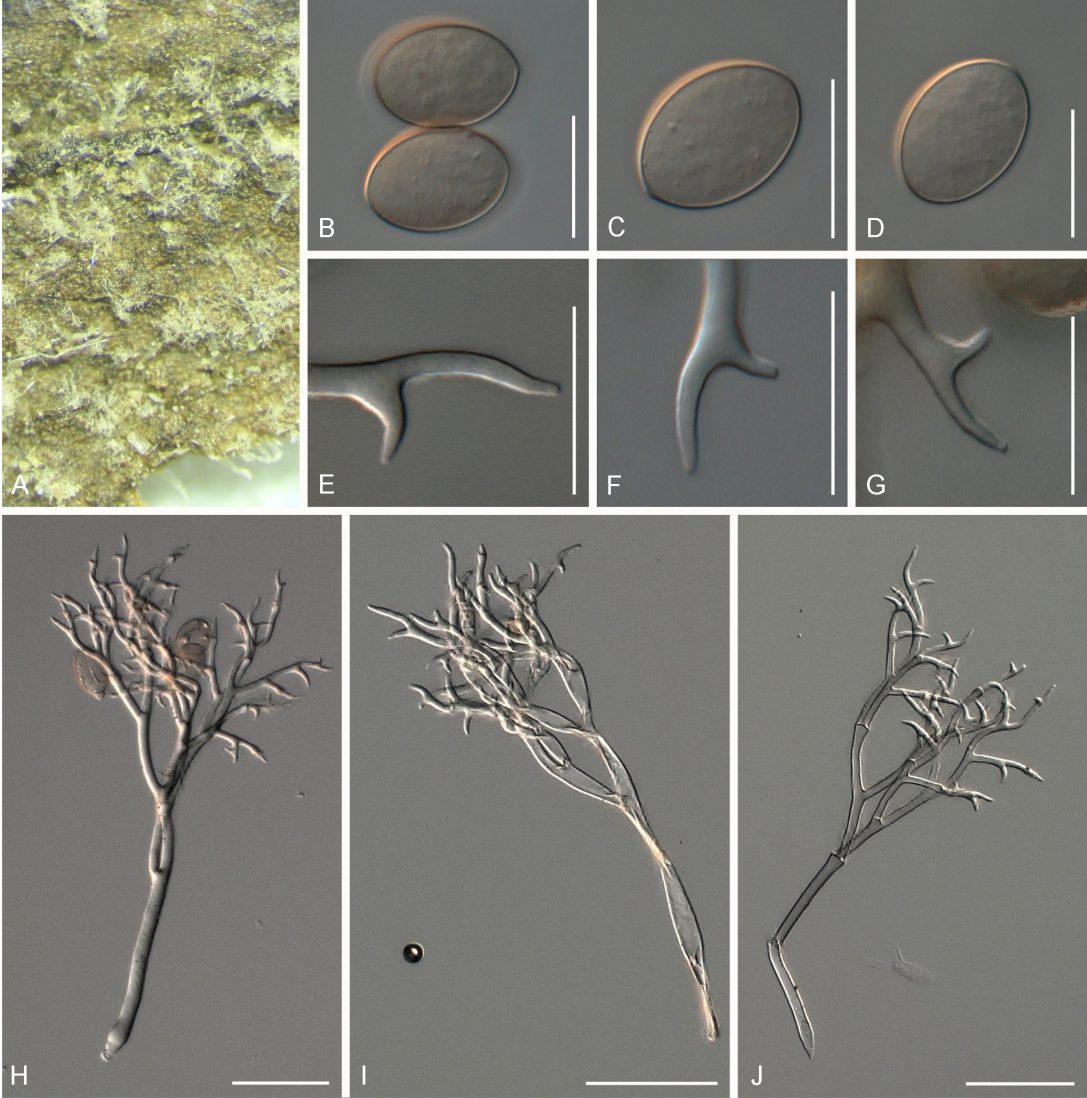
Symptoms and morphology of *Peronospora obscura* parasitic on *Veronica urticifolia* (GLM-F046912). **A** Down on the leaf surface. **B–D** Conidia. **E–G** Ultimate branchlets. **H–J** Conidiophores. Scale bars: 20 µm (**B–G**); 50 µm (**H–J**).

##### Diagnosis.

Differs from *P. palustris* in having more globose conidia, as well as shorter conidiophores and trunks. Differs from *P. silvestris* in having more globose conidia, shorter conidiophores and trunks, narrower trunks and conidiophore bases, as well as longer ultimate branchlets.

##### Type.

AUSTRIA. Tyrol: Steeg, on living leaves of *Veronica urticifolia*, 2 Jun 2000, H. Jage (holotype GLM-F046912).

##### Description.

***Lesions*** on leaves at first yellowish or chlorotic, later often darkening to become dark yellow, often vein-delimited. ***Down*** present on the lower leaf surface, consisting of scattered to dense felt-like conidiophore outgrowth. ***Conidiophores*** hyaline, straight to curved, thin-walled, 279–380 µm long, av. 330 µm; trunk 130–199 µm long, av. 165 µm, 5.2–9.1 µm broad, sometimes slightly swollen to up to 12 µm at the base, ratio of the total length to trunk length 1.5–2.5, callose plugs absent. ***Branching*** subdichotomous in 4–7 orders, with sub-straight to curved branches, gradually attenuate, often at right angles. ***Ultimate branchlets*** sub-straight to curved, paired branchlets differing in length, with the longer ones 9.5–15.5 µm long, the shorter ones 5.5–10.5 µm long, with a ratio of the longer to the shorter ultimate branchlet of 1.3–1.9, base 2–3 µm broad, apex slightly sharp. ***Conidia*** ellipsoid with a pale yellow-brown colour, 25–28.5 µm long, 17.5–19.5 µm broad, length-to-breadth ratio 1.35–1.55, basal part of the conidia mostly protruding. ***Oospores*** not observed.

##### Habitat.

Living leaves of *Veronica urticifolia* and *Paederota lutea* (syn. *Veronica lutea*).

##### Distribution.

Austria, Germany.

##### Additional specimens examined.

AUSTRIA. Carinthia: District of Spittal/Drau, municipality of Flattach, Großfragant, Rollbahnweg, 400 m before Kreuzbödele, on living leaves of *Veronica urticifolia*, 11 Jul 2000, H. Voglmayr (WU-MYC0032785); Tscheppaschlucht S Ferlach on the Loiblstraße, N of the inn *Deutscher Peter*, on living leaves of *Paederota lutea*, 14 Jun 2009, H. Voglmayr (WU-MYC0032793).

##### Notes.

A specimen, AR194, recorded in GenBank as parasitic on *Veronica urticifolia* (Riethmüller et al. 2002), was identified as *P. silvestris*. However, the LSU D1-3 phylogenetic tree reveals that the sequence AY035490 actually forms a clade with our newly described species, *P. obscura*, found on *Veronica urticifolia* and *Paederota lutea*. This indicates that AY035490 does not represent *P. silvestris*, whose type host is *Veronica officinalis*. This distinction is not limited to the LSU D1-3 region but is also evident in the ITS, LSU D6-8, *COX*1, *COX*2, *NAD1*, and *RPS10* regions. Phylogenetic analyses consistently show that *P. obscura* forms a sister clade with two species: *P. silvestris* and *P. palustris*. However, compared to *P. obscura*, they have more elongated conidia, with an average length-to-breadth ratio of both higher than 1.6, longer conidiophores and trunks (average length longer than 400 µm and 250 µm, respectively), shorter ultimate branchlets, with the longer branchlets shorter than 12.2 µm and the shorter branchlets shorter than 7.3 µm on average, and broader conidiophore bases, maximum breadth up to 14 µm, and trunks, average breadth broader than 7.6 µm.

#### Peronospora palustris

Taxon classificationFungiPeronosporalesPeronosporaceae

Gäum., Annales Mycologici 16 (1–2): 198. 1918.

29C24BF9-FD6D-55CC-964B-69E118AF840C

234929

Fig. 12

##### Description.

***Lesions*** on leaves yellow to brown, diffuse to vein-delimited. ***Down*** present on the lower leaf surface, consisting of scattered to dense felt-like conidiophore outgrowth. ***Conidiophores*** hyaline, straight to curved, thin-walled, 335–550 µm long, av. 443 µm; trunk 204–394 µm long, av. 299 µm, 6–9.5 µm broad, sometimes slightly swollen to up to 14 µm at the base, ratio of the total length to trunk length 1.5–2, callose plugs absent. ***Branching*** subdichotomous in 4–8 orders, with sub-straight to curved branches, gradually attenuate, often at acute angles. ***Ultimate branchlets*** sub-straight to curved, paired branchlets differing in length, with the longer ones 10–14.5 µm long, the shorter ones 6–8.5 µm long, with a ratio of the longer to the shorter ultimate branchlet of 1.5–1.9, base 2.5–3 µm broad, apex slightly sharp. ***Conidia*** ellipsoid to elongate with a pale reddish colour, 26–31.5 µm long, 15–20 µm broad, length-to-breadth ratio 1.4–1.95, basal part of the conidia mostly protruding. ***Oospores*** not observed.

**Figure 12.**
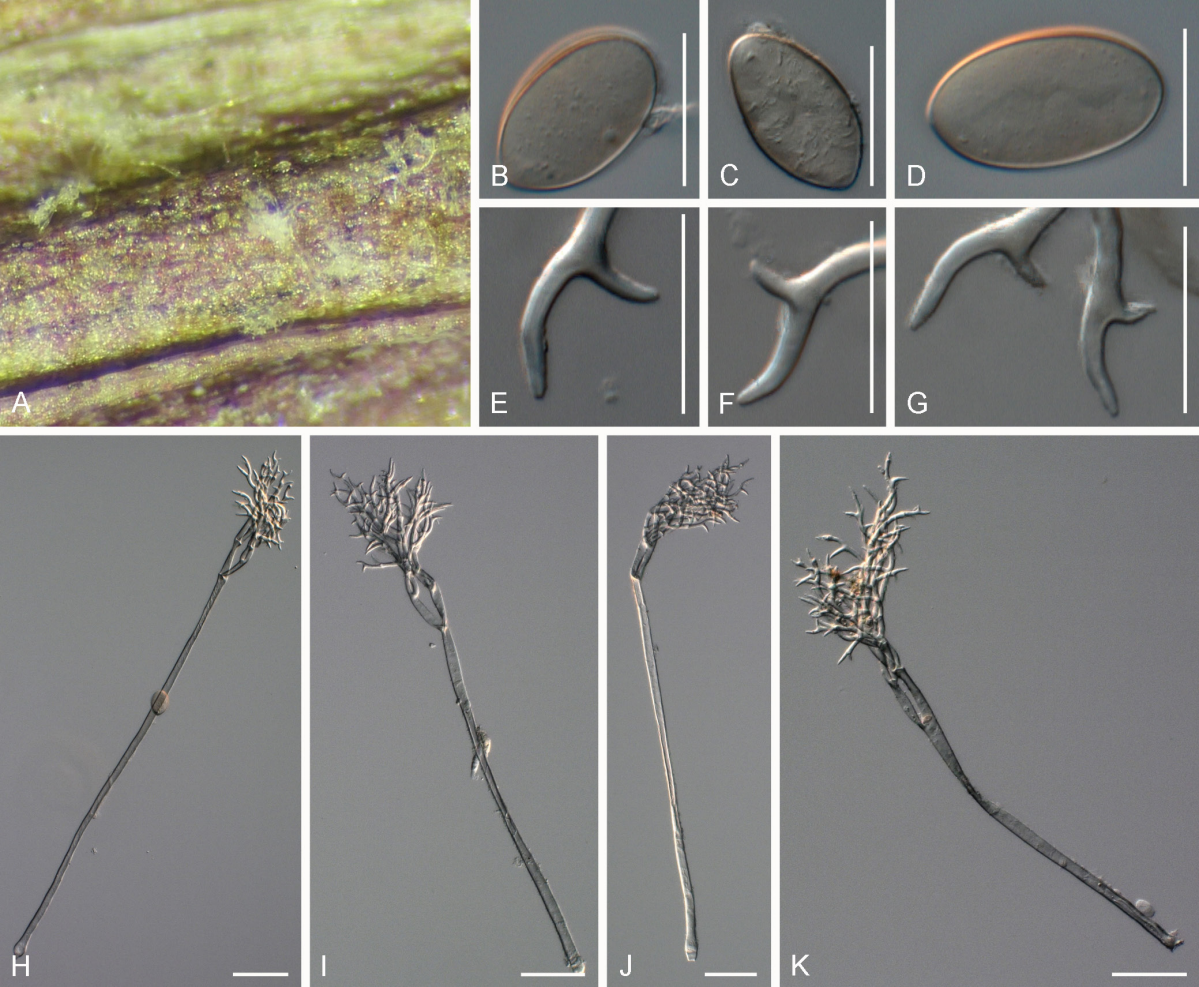
Symptoms and morphology of *Peronospora palustris* parasitic on *Veronica scutellata* (GLM-F074295). **A** Down on the leaf surface. **B–D** Conidia. **E–G** Ultimate branchlets. **H–K** Conidiophores. Scale bars: 20 µm (**B–G**); 50 µm (**H–K**).

##### Type host.

*Veronica scutellata* (Gäumann 1918; Stevenson 1926).

##### Confirmed hosts.

*Veronica armstrongii*, *Veronica diosmifolia*, *Veronica scutellata*.

##### Reported hosts.

*Veronica scutellata* (Gäumann 1918; Stevenson 1926).

##### Reported distribution.

Denmark, Norway, Poland (Gäumann 1923), Switzerland (Stevenson 1926).

##### Specimens examined.

GERMANY. Lower Saxony: Ammerland, on living leaves of *Veronica armstrongii*, 17 Sep 2017, T. Brand (T.B.2017); same location, on living leaves of *Veronica diosmifolia*, 2020, T. Brand (T.B.2020); Saxony-Anhalt: Allstedt, on living leaves of *Veronica scutellata*, 1 Aug 2004, H. Jage (GLM-F062903); Bleddin, Dammfuß, on living leaves of *Veronica scutellata*, 3 Aug 2004, H. Jage (GLM-F065747); Schönebeck, Buschhaus, on living leaves of *Veronica scutellata*, 3 Jul 2003, H. Jage (GLM-F074295).

##### Notes.

*Peronospora palustris* is distributed in Europe and has been reported only from *Veronica scutellata* before this study (Gäumann 1918; Stevenson 1926). However, the current study shows that it is also able to infect the ornamental shrubs *Veronica diosmifolia* (syn. *Hebe diosmifolia*) and *Veronica armstrongii* (syn. *Hebe armstrongii*), likely representing a host shift to a host naïve to the pathogen (Thines 2019). The LSU D1–3 region has insufficient resolution for distinguishing between *P. palustris*, *P. silvestris*, and *P. seminaria*. Similarly, the LSU D6–8 and *RPS10* regions cannot differentiate *P. palustris* from *P. silvestris*. However, these species are distinct based on other loci, including ITS, *COX*1, *COX*2, and *NAD1*. Morphologically, compared to *P. silvestris* and *P. seminaria*, *P. palustris* had more elongated conidia, with an average length of 29 µm and a length-to-breadth ratio of 1.7.

#### Peronospora petricosa

Taxon classificationFungiPeronosporalesPeronosporaceae

M. Mu & Thines sp. nov.

A15FA6A2-976B-59EB-890C-07ACC5D0A8E8

860233

Fig. 13

##### Etymology.

“Petricosus” means “very confusing”, referring to the host *Veronica arvensis*, which has been reported to be parasitised by many *Peronospora* species but seems to be parasitised by only a single species.

**Figure 13.**
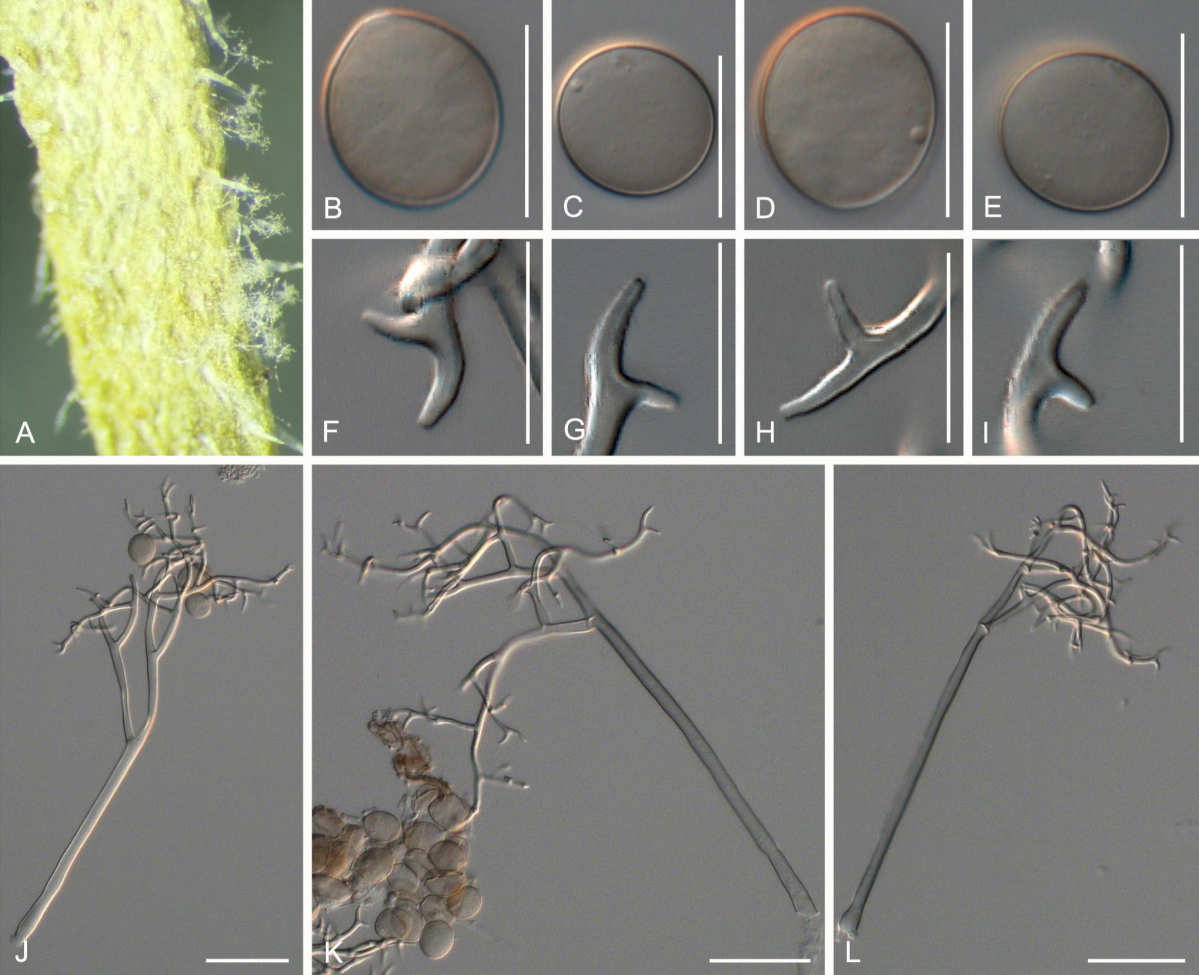
Symptoms and morphology of *Peronospora petricosa* parasitic on *Veronica arvensis* (GLM-F063014). **A** Down on the leaf surface. **B–E** Conidia. **F–I** Ultimate branchlets. **J–L** Conidiophores. Scale bars: 20 µm (**B–I**); 50 µm (**J–L**).

##### Diagnosis.

Differs from *P. microspora* in having more elongated conidia. Differs from *P. brevibrachia* in having more globose conidia, shorter conidiophores and trunks, as well as longer ultimate branchlets.

##### Type.

GERMANY. Saxony-Anhalt: Prositz, Ketzerbachtal, on living leaves of *Veronica arvensis*, 17 Mar 2004, H. Jage (holotype GLM-F063014).

##### Description.

***Lesions*** yellow to dark violet, diffuse to vein-delimited. ***Down*** present on the lower leaf surface, consisting of scattered to dense felt-like conidiophore outgrowth. ***Conidiophores*** hyaline, straight to curved, thin-walled, 237–353 µm long, av. 295 µm; trunk 130–222 µm long, av. 176 µm, 5–7 µm broad, sometimes slightly swollen to up to 15 µm at the base, ratio of the total length to trunk length 1.5–2, callose plugs absent. ***Branching*** subdichotomous in 4–7 orders, with sub-straight to curved branches, gradually attenuate, often at right angles. ***Ultimate branchlets*** sub-straight to curved, paired branchlets differing in length, with the longer ones 8–14 µm long, the shorter ones 6–10.5 µm long, with a ratio of the longer to the shorter ultimate branchlet of 1.15–1.6, base 2–3 µm broad, apex slightly sharp. ***Conidia*** subglobose to ellipsoid with a grey to reddish colour, 18.5–22 µm long, 14.5–17 µm broad, length-to-breadth ratio 1.2–1.4. ***Oospores*** not observed.

##### Habitat.

Living leaves of *Veronica arvensis* and *Veronica odora*.

##### Distribution.

Argentina, Australia, Austria, Germany, South Korea, Switzerland.

##### Additional specimens examined.

ARGENTINA. Unknown, on living leaves of *Veronica arvensis*, 1991, B. Blanca (R.D.854). AUSTRALIA. Paradise Plants, Cherrylane, RMB 2117, Greta Road, Kulnura R.D. 61 on living leaves of *Veronica odora*, 19 May 1993, I. Paananen (DAR69945). AUSTRIA. Lower Austria: District of Bruck/Leitha, Municipality of Prellenkirchen, Spitzerberg, S-Hang S Edelstal, Weingarten, on living leaves of *Veronica arvensis*, 9 Apr 2011, H. Voglmayr (WU-MYC0032799). GERMANY, Saxony-Anhalt: Aseleben, on living leaves of *Veronica arvensis*, 7 Jan 2001, H. Jage (GLM-F074187); Gänsefurth, on living leaves of *Veronica arvensis*, 27 Mar 2004, H. Jage (GLM-F063013); Gröna, on living leaves of *Veronica arvensis*, 11 Apr 1999, H. Jage (GLM-F077818); Kemberg, Kuhgasse, on living leaves of *Veronica arvensis*, 18 Jan 2005, H. Jage (GLM-F065698); Löbejün, on living leaves of *Veronica arvensis*, 13 May 2002, H. Jage (GLM-F078032); Winkel, on living leaves of *Veronica arvensis*, 8 Apr 2004, H. Jage (GLM-F063058); Saxony: Bad Muskau, on living leaves of *Veronica arvensis*, 17 May 2005, H. Jage (GLM-F075728); Görlitz-Nikolaivorstadt, on living leaves of *Veronica arvensis*, 17 Apr 2004, H. Jage (GLM-F063028); Halbendorf, on living leaves of *Veronica arvensis*, 15 May 2005, H. Jage (GLM-F075720). SOUTH KOREA. Gangwon: Gangneung, near Gangneung-Wonju National University, on living leaves of *Veronica arvensis*, 12 May 2000, Y-J. Choi (KUS-F17294); Gyeonggi-do: Suwon-si, near Horticultural Research Institute, on living leaves of *Veronica arvensis*, 24 May 2011, Y-J. Choi (KUS-F25712). SWITZERLAND. Canton of Bern: Blatten, Lötschenthal, on living leaves of *Veronica arvensis*, 1 Jun 1941, S. Blumer (ZT81370).

##### Notes.

*Veronica arvensis* is native to Macaronesia, North-West Africa, Europe, southwestern Siberia, and the western Himalayas. Due to human activity, this host plant has been widely introduced and has become invasive in several regions, including North and South America and Australasia (Kew Science 2024). *Veronica arvensis* has been reported as a host for *P. agrestis* (Dennis 1986) and *P. grisea* (de Bary 1863). However, our phylogenetic analysis indicates that the *Peronospora* species found on *Veronica arvensis* are distinct from *P. agrestis* on *Veronica polita* and *P. grisea* on *Veronica beccabunga*. Additionally, there is a specimen, MG1969, collected from *Veronica arvensis* that has been previously identified as *P. verna* (Göker et al. 2003, 2007). However, all *Peronospora* specimens on *Veronica arvensis* are phylogenetically grouped together in a distinct clade, suggesting that the *Peronospora* species on *Veronica arvensis* represents a single, previously unrecognised species, for which we propose to name *P. petricosa*. Although numerous *Veronica arvensis* samples have been collected from many countries, including Argentina, Australia, Austria, Germany, South Korea, and Switzerland, only one sample has been found on *Veronica odora* (syn. *Hebe buxifolia*). This may be due to the widespread distribution of the host facilitated by human activity, leading to infections of this host, naïve to *Peronospora* in its native range, in some regions. A similar case can be seen with *Peronospora gaponenkoae*, initially found on *Plantago lanceolata*. Davis et al. (2021) described a *Peronospora* species on *Plantago princeps* as *P. kuewa*, which was later shown to be synonymous with *P. gaponenkoae*, which shifted hosts to the Hawai’ian indigenous host (Mu et al. 2024a). This case highlights that host jumping and range expansion occur more frequently than previously thought. Morphologically, *P. petricosa* is characterised by having the narrowest trunks, with an average breadth of 6 µm. Compared to the closely related species, *P. microspora* had more globose conidia, with a length-to-breadth ratio of 1.25 ± 0.1, while *P. brevibrachia* had more elongated conidia, a length-to-breadth ratio of 1.4 ± 0.1, as well as longer conidiophores and trunks, averaging 380 µm and 240 µm, respectively, as well as shorter ultimate branchlets, the longer ones averaging 10 µm and the shorter ones 7 µm.

#### Peronospora sagittaria

Taxon classificationFungiPeronosporalesPeronosporaceae

M. Mu & Thines sp. nov.

491E6E79-4B22-5A1C-B379-C00ED2057E37

860234

Fig. 14

##### Etymology.

The Latin word “sagittarius” means archer, referring to the ultimate branchlets of the species, which are curved and shaped like a bow and arrow.

**Figure 14.**
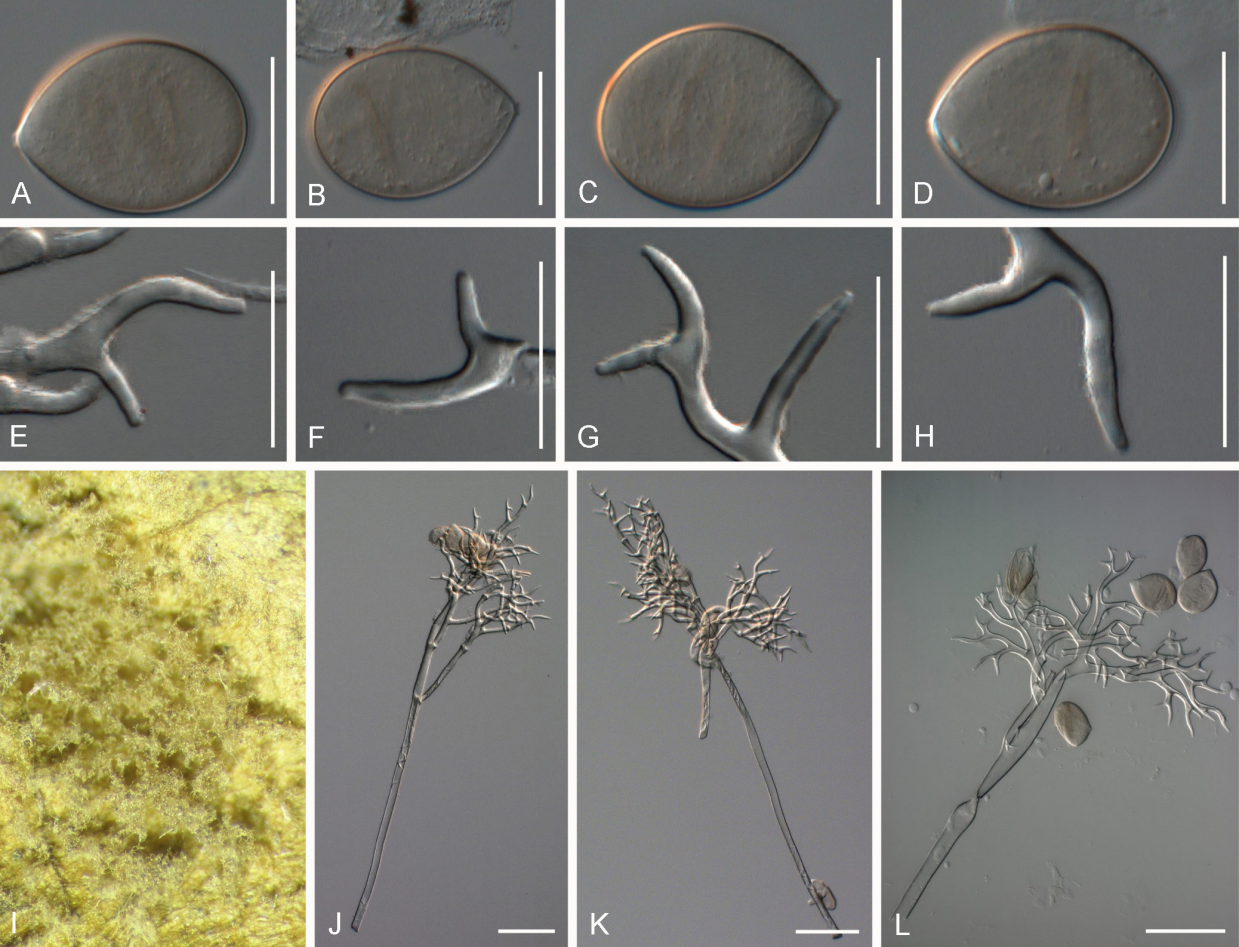
Symptoms and morphology of *Peronospora sagittaria* parasitic on *Veronica catenata* (GLM-F074298). **A–D** Conidia. **E–H** Ultimate branchlets. **I** Down on the leaf surface. **J–L** Conidiophores. Scale bars: 20 µm (**A–H**); 50 µm (**J–L**).

##### Diagnosis.

Differs from *P. aquatica* in having smaller conidia, longer conidiophores and trunks, as well as narrower trunks and conidiophore bases.

##### Type.

GERMANY. Saxony-Anhalt: Schönebeck, Buschhaus, on living leaves of *Veronica catenata*, 3 Jul 2003, H. Jage (holotype GLM-F074298).

##### Description.

***Lesions*** on leaves greyish-white to light blue-purple, diffuse to vein-delimited. ***Down*** present on the lower leaf surface, sparse, consisting of scattered to dense felt-like conidiophore outgrowth. ***Conidiophores*** hyaline, straight to curved, thin-walled, 319–490 µm long, av. 405 µm; trunk 178–307 µm long, av. 243 µm, 6.5–9.5 µm broad, sometimes slightly swollen to up to 15 µm at the base, ratio of the total length to trunk length 1.5–2, callose plugs absent. ***Branching*** subdichotomous in 4–7 orders, with sub-straight to curved branches, gradually attenuate, often at acute angles. ***Ultimate branchlets*** sub-straight to curved, paired branchlets differing in length, with the longer ones 12–23.5 µm long, the shorter ones 7.5–14.5 µm long, with a ratio of the longer to the shorter ultimate branchlet of 1.3–1.9, base 2.5–3 µm broad, apex slightly sharp. ***Conidia*** ellipsoid to ovate with a brown to dark grey colour, 28.7–35 µm long, 19–23.5 µm broad, length-to-breadth ratio 1.35–1.7, basal part of the conidia mostly protruding. ***Oospores*** not observed.

##### Habitat.

Living leaves of *Veronica catenata*.

##### Distribution.

Germany.

##### Additional specimens examined.

GERMANY. Saxony-Anhalt: Döllnitz, on living leaves of *Veronica catenata*, 10 Oct 2005, H. Jage (GLM-F075865).

##### Notes.

The LSU D1-3 tree analysis indicated that *P. sagittaria* on *Veronica catenata* and *P. aquatica* on *Veronica anagallis-aquatica* represent the same taxa. However, phylogenetic analyses using a multi-locus approach including ITS, LSU D6-8, *COX*1, *COX*2, *NAD1*, and *RPS10* sequences demonstrate that they are in fact distinct species. This finding suggests that the LSU D1-3 locus alone may not always provide sufficient resolution for distinguishing certain closely related species, reinforcing the need for multi-locus approaches for accurate identification. These results are consistent with the conclusions of Choi et al. (2015b). The LSU D6-8 (AY273956) and *NAD*1 (DQ361185) phylogenetic trees showed *P. sagittaria* on *Veronica catenata* is the same taxon as specimen MG1968, which was collected from *Veronica anagallis-aquatica* (Göker et al. 2003, 2007). Apparently, MG1968 had been misidentified in terms of its host plant. Morphologically, *Veronica catenata* is distinguished by having leaves with the broadest part in the lower third and more pointed tips, whereas *Veronica anagallis-aquatica* has leaves with the broadest part at the middle and less pointed tips, but otherwise look rather similar, especially when not in the flowering state. Both ITS and *matK* sequences provided a clear distinction between these two species, further supporting an identification mistake. The concatenated phylogenetic tree suggests that the *Peronospora* species on *Veronica catenata* represents a previously undescribed species, distinct from *P. aquatica*. While these two taxa are sister species and phylogenetically closely related, compared to *P. sagittaria* morphologically, *P. aquatica* is characterised by more elongated conidia with an average length of 33 µm and an average length-to-breadth ratio of 1.6, as well as shorter conidiophores and trunks, averaging 348 µm and 190 µm, respectively, and broader trunks and conidiophore bases, averaging 9 µm and 13 µm, respectively.

#### Peronospora seminaria

Taxon classificationFungiPeronosporalesPeronosporaceae

M. Mu & Thines sp. nov.

BF1A15A0-83EC-5C4C-88DE-60A64FDE1718

860235

Fig. 15

##### Etymology.

The epithet “seminaria” is derived from the Latin “seminarium”, meaning seedbed or nursery. It refers to the host plant of the holotype, which originates from an ornamental plant farm near Osnabrück.

**Figure 15.**
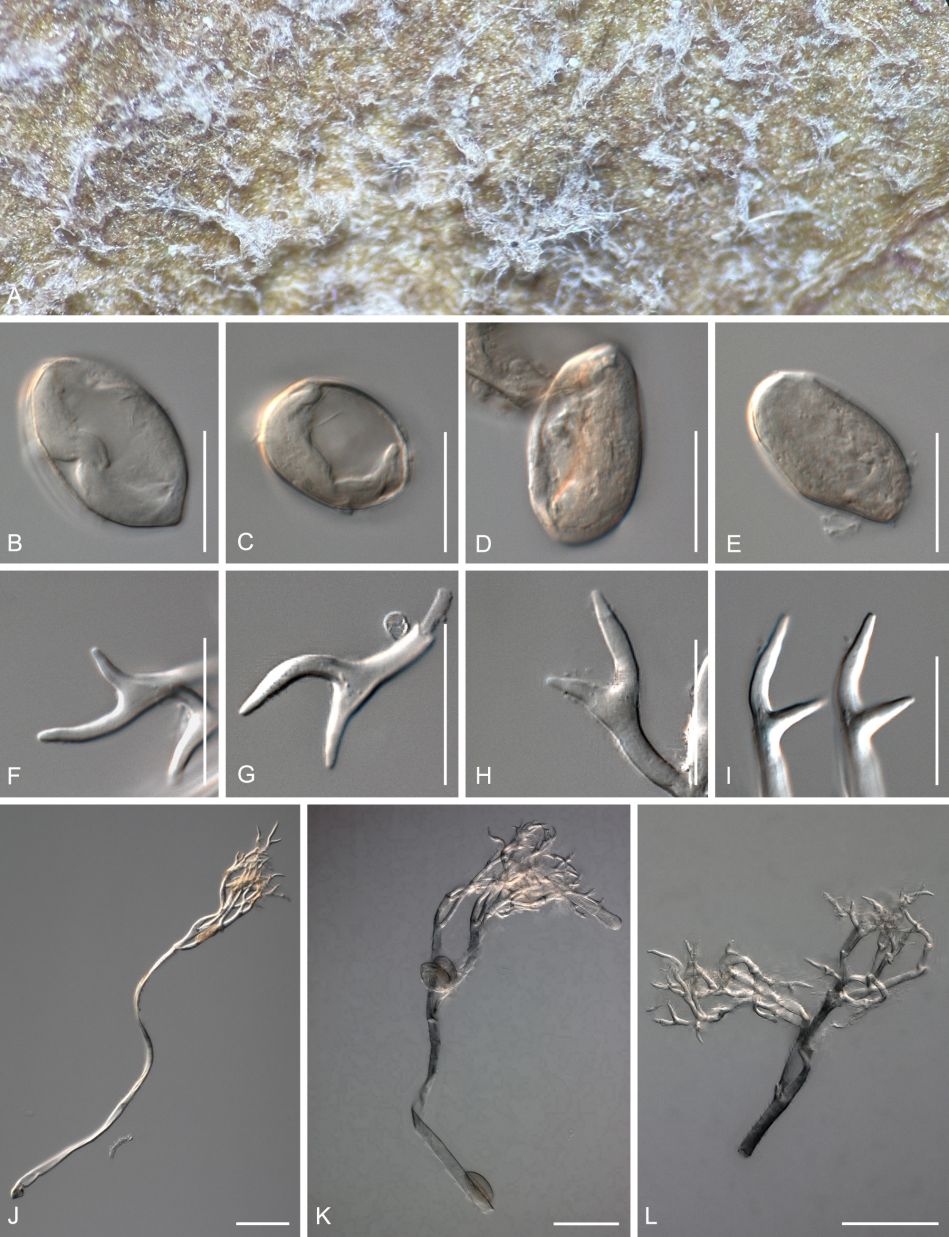
Symptoms and morphology of *Peronospora seminaria* parasitic on *Veronica longifolia* (GLM-F125572). **A** Down on the leaf surface. **B–E** Conidia. **F–I** Ultimate branchlets. **J–L** Conidiophores. Scale bars: 20 µm (**B–I**); 50 µm (**J–L**).

##### Diagnosis.

Differs from *P. aquatica* in having smaller and elongated conidia, narrower trunks, as well as shorter ultimate branchlets. Differs from *P. sagittaria* in having smaller and elongated conidia, shorter conidiophores and trunks, as well as shorter ultimate branchlets.

##### Type.

GERMANY. Lower Saxony: near Osnabrück, on living leaves of *Veronica longifolia*, 7 Jun 2023, T. Brand (holotype GLM-F125572).

##### Description.

***Lesions*** chlorotic to dark violet, diffuse to vein-delimited. ***Down*** present on the lower leaf surface, consisting of scattered to dense felt-like conidiophore outgrowth. ***Conidiophores*** hyaline, sub-straight to curved, thin-walled, 221–508 µm long, av. 365 µm; trunk 103–257 µm long, av. 180 µm, 6.5–9.5 µm broad, sometimes slightly swollen to up to 16 µm at the base, ratio of the total length to trunk length 1.5–2, callose plugs absent. ***Branching*** subdichotomous in 4–8 orders, with sub-straight to curved branches, gradually attenuate, often at acute angles. ***Ultimate branchlets*** sub-straight to curved, paired branchlets differing in length, with the longer ones 8–13.5 µm long, the shorter ones 5–9.5 µm long, with a ratio of the longer to the shorter ultimate branchlet of 1.3–1.7, base 2–3 µm broad, apex slightly sharp. ***Conidia*** ellipsoid to elongate with a pale brown to pale grey colour, 17–27 µm long, 10.5–17 µm broad, length-to-breadth ratio 1.45–1.75, basal part of the conidia mostly protruding. ***Oospores*** not observed.

##### Habitat.

Living leaves of *Veronica longifolia*.

##### Distribution.

Germany.

##### Additional specimens examined.

AUSTRIA. Lower Austria: District of Gänserndorf, Municipality of Ringelsdorf-Niederabsdorf, Große Wiesen, on living leaves of *Veronica longifolia*, 5 May 2012, H. Voglmayr and I. Greilhuber (WU-MYC0032814). CZECH REPUBLIC. Moravia: N Brno, N Křtiny, near Arboretum, species-rich wet meadow, on living leaves of *Veronica longifolia*, 23 Jun 2011, H. Voglmayr and I. Greilhuber (WU-MYC0032804).

##### Notes.

*Veronica longifolia* is widely distributed across the Northern Hemisphere, yet no reports of infection by downy mildew have been documented. In this study, we identified a previously undescribed *Peronospora* species that forms a clade with five closely related species: *P. obscura*, *P. sagittaria*, *P. aquatica*, *P. palustris*, and *P. silvestris*. This clade is characterised by elongated conidia with an average length-to-breadth ratio higher than 1.45. Morphologically, *P. seminaria* differs by having smaller conidia, averaging 22 µm × 14 µm, and the narrowest conidia among all *Peronospora* species on *Veronica*, with an average breadth of 14 µm.

#### Peronospora silvestris

Taxon classificationFungiPeronosporalesPeronosporaceae

Gäum., Annales Mycologici 16 (1–2): 199. 1918.

F9427707-5EB1-5E0D-933C-280F9B9FF63B

142535

Fig. 16

##### Description.

***Lesions*** often hardly visible and systemic, infected plant parts often discolouring at later stages, drying or getting necrotic in ***Veronica officinalis***; lesions chlorotic, then turning dark brown to brown upon drying, often vein-delimited in ***Globularia nudicaulis***. ***Down*** present on the lower leaf surface, consisting of scattered to dense felt-like conidiophore outgrowth. ***Conidiophores*** hyaline, straight to curved, thin-walled, 332–490 µm long, av. 441 µm; trunk 203–309 µm long, av. 256 µm, 6–9.5 µm broad, sometimes slightly swollen to up to 14 µm at the base, ratio of the total length to trunk length 1.5–2, callose plugs absent. ***Branching*** subdichotomous in 4–7 orders, with sub-straight to curved branches, gradually attenuate, often at acute angles. ***Ultimate branchlets*** sub-straight to curved, paired branchlets differing in length, with the longer ones 9.5–14.5 µm long, the shorter ones 6–8.5 µm long, with a ratio of the longer to the shorter ultimate branchlet of 1.4–1.9, base 2–2.5 µm broad, apex slightly sharp. ***Conidia*** ellipsoid to elongate with a dark brown colour, 24–31 µm long, 15–20 µm broad, length-to-breadth ratio 1.4–1.85, basal part of the conidia mostly protruding. ***Oospores*** not observed.

**Figure 16.**
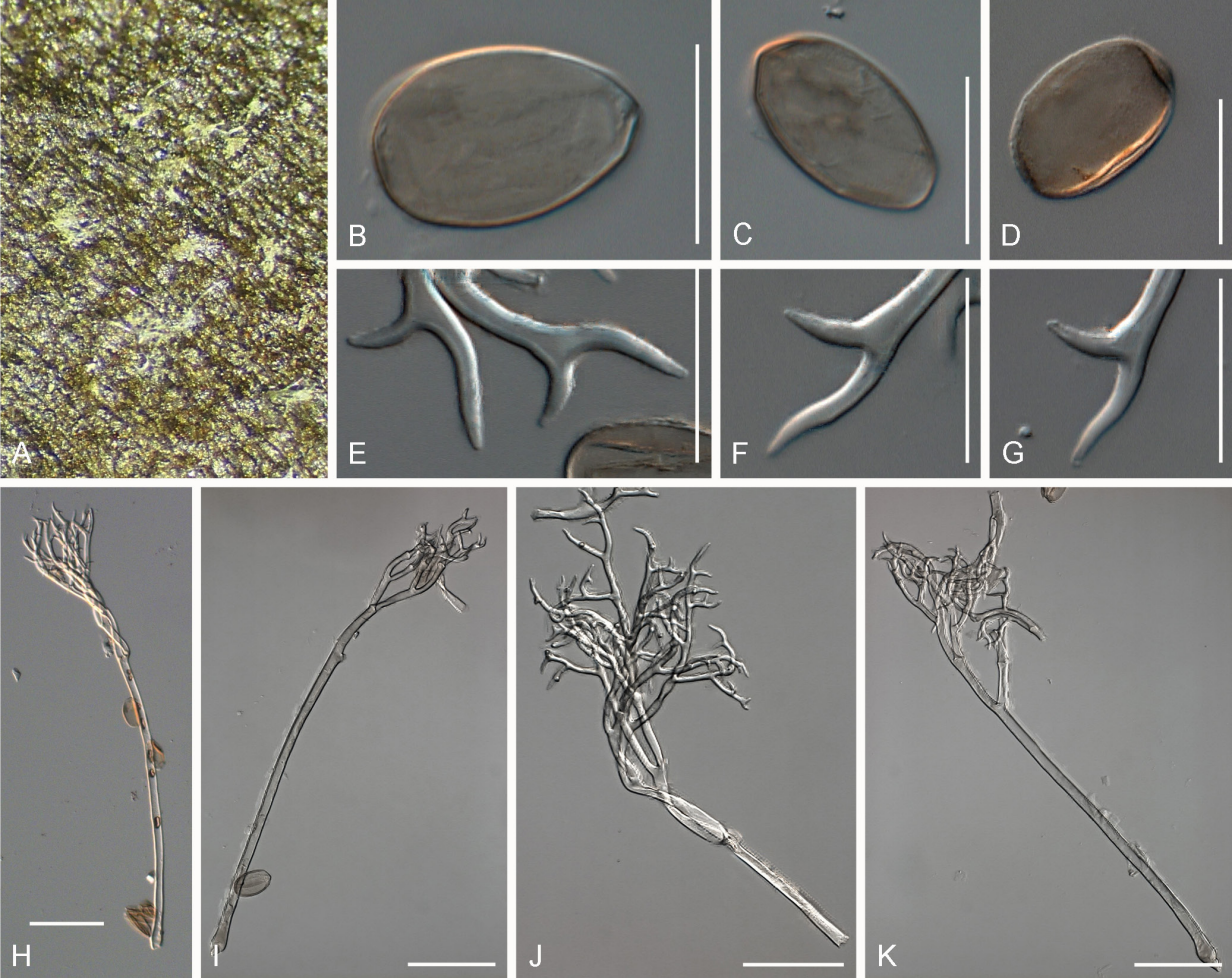
Symptoms and morphology of *Peronospora silvestris* parasitic on *Globularia nudicaulis* (GLM-F135692). **A** Down on the leaf surface. **B–D** Conidia. **E–G** Ultimate branchlets. **H–K** Conidiophores. Scale bars: 20 µm (**B–G**); 50 µm (**H–K**).

##### Type host.

*Veronica officinalis* (Gäumann 1918).

##### Confirmed hosts.

*Veronica officinalis* and *Globularia nudicaulis*.

##### Reported hosts.

*Veronica officinalis* (Gäumann 1918), *V. urticifolia* (Gäumann 1923; Riethmüller et al. 2002).

##### Reported distribution.

Austria, Denmark, France, Germany, Italy. Spain, Sweden, Switzerland (Gäumann 1923; Riethmüller et al. 2002).

##### Specimens examined.

AUSTRIA. Styria: District of Judenburg, Municipality of Pusterwald, forest path near Assmann, on living leaves of *Veronica officinalis*, 14 Jun 2011, H. Voglmayr and I. Greilhuber (WU-MYC0032802); District of Judenburg, Municipality of Pusterwald, Hinterwinkel, Waldschlag at Vorderer Pölsenbach, on living leaves of *Veronica officinalis*, 14 Jun 2011, H. Voglmayr and I. Greilhuber (WU-MYC0032803). SWITZERLAND. Canton of Bern: Boltigen, Berner Oberland, on living leaves of *Globularia nudicaulis*, 29 Jul 2016, T. Brodtbeck (GLM-F135693); Canton of Uri: Spiringen, Klausenpass, on living leaves of *Globularia nudicaulis*, 24 Aug 2016, T. Brodtbeck (GLM-F135692).

##### Notes.

Gäumann (1918) described *P. silvestris* on *Veronica officinalis*, the type host, but also recorded it on *Veronica urticifolia* (Gäumann 1918, 1923). Subsequently, a specimen (AR194) collected from *Veronica urticifolia* was identified as *P. silvestris* based on its host (Riethmüller et al. 2002). However, phylogenetic analysis based on the LSU sequence (AY035490) placed specimens from these hosts on a separate branch, with the parasite of *V. urticifolia* being a previously unrecognised species, *P. obscura*. Morphologically, *P. silvestris* is characterised by more globose conidia, with a length-to-breadth ratio of 1.6 ± 0.2, distinguishing it from its sister species, *P. palustris*.

#### Peronospora veronicae-cymbalariae

Taxon classificationFungiPeronosporalesPeronosporaceae

Rayss, Palestine Journal of Botany 3: 160. 1945.

A6E448DA-6115-5EF0-90F5-B38EF2813D1D

289173

Fig. 17

##### Description.

***Conidiophores*** hyaline, straight to sub-straight, slightly curved, 208–284 µm long, av. 246 µm; trunk 88–145 µm long, av. 117 µm, 8–11.5 µm broad, sometimes slightly swollen to up to 16 µm at the base, ratio of the total length to trunk length 2–2.5, callose plugs absent. ***Branching*** subdichotomous in 4–5 orders, with sub-straight to slightly curved branches, gradually attenuate, often at right angles. ***Ultimate branchlets*** with straight to sub-straight, paired branchlets differing in length, with the longer ones 11–18 µm long, the shorter ones 7–12 µm long, with a ratio of the longer to the shorter ultimate branchlet of 1.3–1.75, base 2.5–3.5 µm broad, apex slightly sharp. ***Conidia*** ellipsoid to ovate with a grey colour, 27.5–36.5 µm long, 20–26 µm broad, length-to-breadth ratio 1.2–1.6, basal part of the conidia mostly protruding. ***Oospores*** 30–57 µm in diameter.

**Figure 17.**
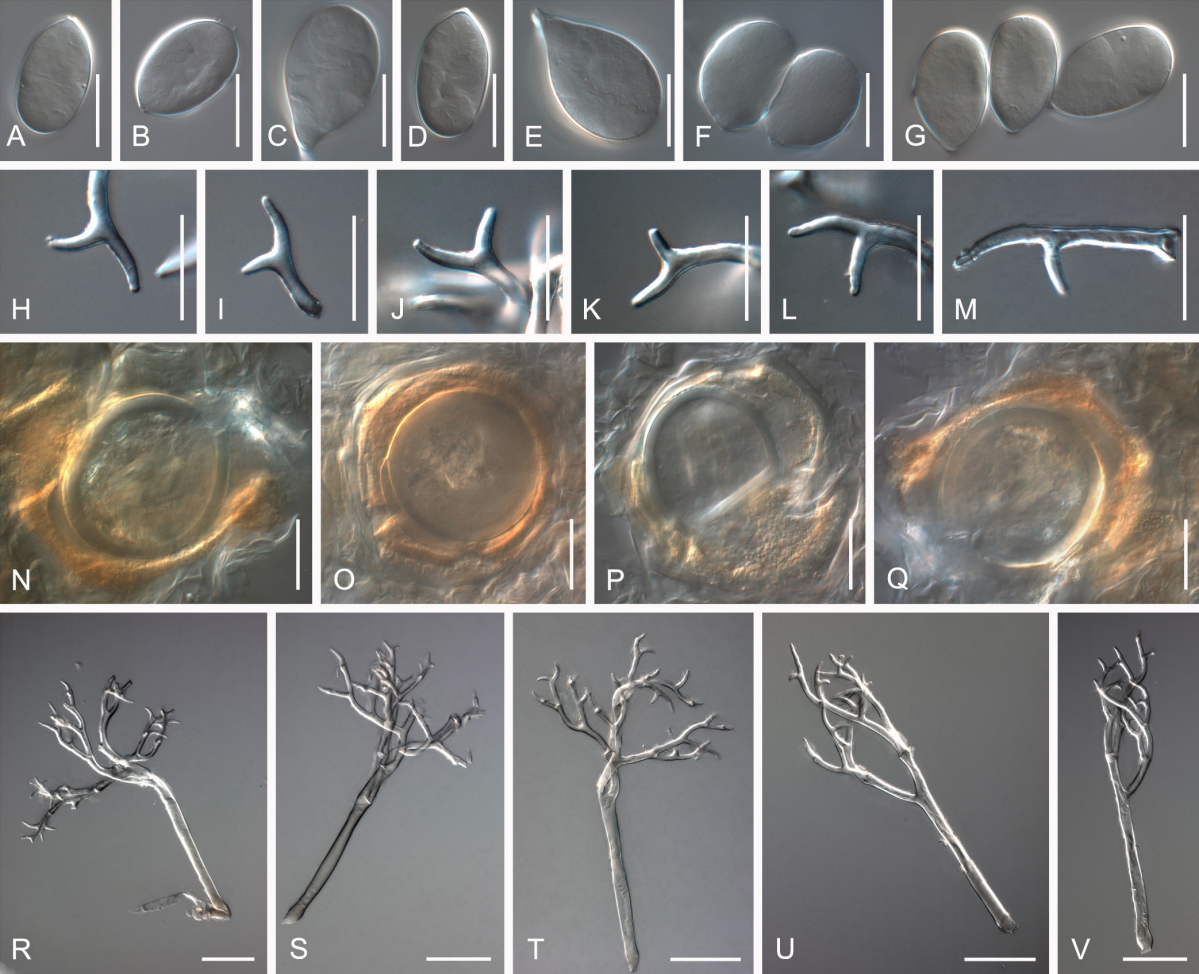
Morphology of *Peronospora veronicae-cymbalariae* parasitic on *Veronica cymbalaria* (ZT85386). **A–G** Conidia. **H–M** Ultimate branchlets. **N–Q** Oospores. **R–V** Conidiophores. Scale bars: 20 µm (**A–Q**); 50 µm (**R–V**).

##### Type host.

*Veronica cymbalaria* (Rayss 1945).

##### Reported distribution.

Palestine, Greece, Spain (Rayss 1945; this study).

##### Specimens examined.

GREECE. Corfu: Troumpettas, Ag. Anna, near the junction, fire site, on the living leaves of *Veronica cymbalaria*, 21 Apr 2012, H. Voglmayr (WU-MYC0032810); between Ano Garouna and Vouniatades, near Ag. Athanasios, on the living leaves of *Veronica cymbalaria*, 22 Apr 2012, H. Voglmayr (WU-MYC0032812); Troumpettas to Ag. Anna, olive grove, on the living leaves of *Veronica cymbalaria*, 23 Apr 2012, H. Voglmayr (WU-MYC0032813). PALESTINE. Without exact locality: on the living leaves of *Veronica cymbalaria*, 5 May 1937, T. Rayss (type ZT85386). SPAIN. Andalusia: Cadiz Province, SE Alcala de los Gazules, near El Jautor, Auwald, on the living leaves of *Veronica cymbalaria*, 17 Mar 2011, H. Voglmayr and W. Jaklitsch (WU-MYC0032796).

##### Notes.

*Peronospora veronicae-cymbalariae* was first identified in Western Asia, specifically in Palestine, in 1945 on *Veronica cymbalaria* (Rayss, 1945). Following its initial description, no additional records of this species were reported for several decades. However, since 2010, specimens of *P. veronicae-cymbalariae* have been collected in Europe. Notably, it has been newly recorded in Greece and Spain. Phylogenetically, as previously noted, *P. veronicae-cymbalariae* has a sister species, *Peronospora* sp. on *Veronica triloba*. While no samples of *Peronospora* sp. were available for detailed description in this study, it is worth mentioning that nuclear loci fail to effectively distinguish between these two species. In contrast, mitochondrial loci provide clear differentiation, emphasising the importance of multi-locus approaches in phylogenetic studies (Choi et al. 2015b). Morphologically, compared to other *Peronospora* species infecting *Veronica*, *P. veronicae-cymbalariae* is characterised by having the shortest conidiophores and trunks, averaging 246 µm and 117 µm long, respectively, the highest conidiophore-to-trunk length ratio, up to 3.3, and the larger conidia, averaging 32 × 23 µm, with conidia that are the broadest among the species examined, up to 32 µm broad.

## Discussion

Although *Peronospora* is the most species-rich genus within the *Oomycota*, with more than 400 species described to date (Constantinescu 1991), the majority of species in this genus are likely yet to be discovered. Identifying *Peronospora* species presents significant challenges due to the presence of numerous morphologically similar downy mildew species (Spring and Thines 2004; Thines and Choi 2016). In the second half of the 20^th^ century, species identification was mostly based on a broad species concept as identification was hampered by the lack of clear morphological features to distinguish closely related species. Moreover, infection trials, especially those involving numerous isolates, were logistically challenging. Before the advent of molecular phylogenies, the broad species concept, though not supported by clear evidence, influenced decisions on whether species should be separated or merged. Yerkes and Shaw (1959) proposed a broad species concept as a practical solution, which subsequently led to frequent misidentifications, inaccurate synonymy, and incorrect assessments of species boundaries (Choi and Thines 2014; Choi et al. 2015b).

There are only some rare cases where a single barcode locus cannot provide sufficient resolution to distinguish certain closely related sister species (Choi et al. 2015b). For instance, the LSU D1-3 locus alone fails to effectively differentiate *P. sagittaria* on *Veronica catenata* from *P. aquatica* on *Veronica anagallis-aquatica*. However, other loci, including ITS, LSU D6–8, *COX*1, *COX*2, *NAD1*, and *RPS10*, provide a clear separation between these species. Similarly, *P. fidelia* and *P. gracilis* show only two gaps in the ITS region and a single base difference in the LSU D1–3 region, with no variation in LSU D6–8. However, substantial differences were found in the four mitochondrial loci (*COX*1, *COX*2, *NAD1*, and *RPS*10). A comparable pattern is observed between *Peronospora* sp. on *Veronica triloba* and *P. veronicae-cymbalariae* on *Veronica cymbalaria* in this study, where nuclear loci fail to effectively distinguish these species. This phenomenon, particularly in the LSU regions, has been reported previously (Choi et al. 2015b) but is less common in the ITS region. However, the low sequence divergence often present in *Peronospora* calls for multi-locus approaches for accurate species identification in the genus (Choi et al. 2015b; Mu et al. 2024a, b, c).

Likewise, accurate identification of some hosts based solely on morphology can be challenging, complicating the determination of the appropriate host for downy mildew species. Taken together, these limitations have confounded species boundaries and insights on the epidemiology of *Peronospora* species and other obligate pathogens. *Peronospora* on *Veronica* is a good showcase for this. For example, *P. agrestis* has been reported to parasitise five host species: *Veronica agrestis*, *V. arvensis*, *V. polita*, *V. opaca*, and *V. chamaedrys* (Gäumann 1918; Stevenson 1926; Notizen 1934; Dennis 1986; Voglmayr 2003). *Peronospora arvensis* has been reported from two host species: *Veronica hederifolia* and *V. triphyllos* (Gäumann 1918, 1923; Stevenson 1926; Klenke and Scholler 2015). *Peronospora grisea* was documented parasitising five host species: *Veronica arvensis*, *V. beccabunga*, *V. hederifolia*, *V. peregrina*, and *V. verna* (Unger 1847; de Bary 1863; Swingle 1887; Gäumann 1923; Stevenson 1926). Additionally, *P. verna* was reported from nine host species: *Veronica arvensis*, *V. chamaedrys*, *V. praecox*, *V. prostrata*, *V. serpyllifolia*, *V. speciosa*, *V. teucrium*, *V. tournefortii*, and *V. verna* (Gäumann 1918, 1923). This suggests overlapping host ranges and might be seen as a hint at a single *Peronospora* species with a broad host range infecting *Veronica*. However, in line with previous studies that have found a high degree of host specificity in *Peronospora* (García-Blázquez et al. 2008; Voglmayr et al. 2014; Hoffmeister et al. 2020; Ploch et al. 2022), this study also found that host-parasite relationships are mostly more restricted than previously thought. Several *Veronica* species appear to be exclusively parasitised by specific *Peronospora* species, for instance, *Veronica arvensis* by *P. petricosa*, *V. chamaedrys* by *P. brevibrachia*, *V. triphyllos* by *P. fidelia*, *V. hederifolia* by *P. arvensis*, *V. verna* by *P. chionodendron*, *V. teucrium* by *P. microspora*, and *V. urticifolia* by *P. obscura*.

In the context of downy mildew evolution and diversification, further evidence was found for a trend suggesting that the relatedness of *Peronospora* species is reflected in their morphological characteristics (Mu et al. 2024a, b). For example, *Peronospora* species parasitising *Veronica* form two major clades in all phylogenetic trees. Morphologically, species of the strongly supported clade 1, such as *P. agrestis*, *P. arvensis*, *P. brevibrachia*, *P. chionodendron*, *P. fidelia*, *P. gracilis*, *P. grisea*, *P. microspora*, *P. petricosa* and *P. veronicae-cymbalariae* are characterised by more globose conidia with average length-to-breadth ratio of less than 1.4 (1.32, if building an average across species). In contrast, *P. obscura*, *P. silvestris*, *P. palustris*, *P. sagittaria*, and *P. aquatica*, which form a highly supported clade 2, feature elongated conidia with average length-to-breadth ratio greater than 1.45 (1.58 across species).

Within the genus *Veronica*, Europe harbours by far the highest number of recorded *Peronospora* species and host associations, whereas only a limited number of species has been reported from North America and Asia. This uneven geographic pattern suggests that diversification within the *Peronospora*–*Veronica* association has been more extensive in Europe, while the lower diversity observed elsewhere may reflect a combination of more recent establishment and lower sampling intensity.

Another emerging topic, not only in *Peronospora* but also other obligate plant pathogens, is the fact that species naïve to infections by a pathogen group during their evolution are prone to be affected by pathogens shifting hosts (Thines 2019). Evidence for this has been found, e.g. in *Erysiphe* (Kusch et al. 2024; Lebeda et al. 2024), *Peronosclerospora* (Telle et al. 2011; Suharjo et al. 2020; Crouch et al. 2022; Muis et al. 2023), *Plasmopara* (Kruse et al. 2016), and *Peronospora* (Thines et al. 2019, 2020). In this study, additional examples are added – downy mildew on *Veronica* subgenus *Hebe*, with *P. palustris* and *P. petricosa* being able to infect *Hebe armstrongii* and *Hebe odora*, respectively. The natural distribution range of the subgenus *Hebe* does not overlap with the native ranges of the primary hosts of *P. palustris* and *P. petricosa*, and there are no confirmed native *Peronospora* species in Oceania, so it can be assumed that the subgenus *Hebe* evolved in the absence of the genus *Peronospora*, rendering it susceptible to species infecting evolutionarily close hosts due to the likely presence of operable effector targets (Thines 2019). The finding of host shifts of *Peronospora* from herbaceous *Veronica* species to members of the subgenus *Hebe* suggests that care should be taken to avoid additional spill-overs, as they could potentially lead to global pandemics, such as in *P. belbahrii*, due to global trade with seeds and plants (Belbahri et al. 2005; Thines et al. 2009).

The high diversity of downy mildews infecting *Veronica* species found in this study highlights the need for ongoing research into species diversity within the genus *Peronospora*, particularly those infecting economically important plants. As *Peronospora* species are generally highly host-specific, but host shifts may occur to exotic plants, detailed investigations, especially on *Peronospora* infections newly occurring on economically important plants, are warranted, and active measures should be taken as soon as new occurrences of downy mildew on traded plant species are observed to prevent these pathogens from spreading and causing economic losses in agriculture and horticulture globally.

## Supplementary Material

XML Treatment for Peronospora agrestis

XML Treatment for Peronospora aquatica

XML Treatment for Peronospora arvensis

XML Treatment for Peronospora brevibrachia

XML Treatment for Peronospora chionodendron

XML Treatment for Peronospora fidelia

XML Treatment for Peronospora gracilis

XML Treatment for Peronospora grisea

XML Treatment for Peronospora microspora

XML Treatment for Peronospora obscura

XML Treatment for Peronospora palustris

XML Treatment for Peronospora petricosa

XML Treatment for Peronospora sagittaria

XML Treatment for Peronospora seminaria

XML Treatment for Peronospora silvestris

XML Treatment for Peronospora veronicae-cymbalariae

## Additional information

### Use of AI

No use of AI was reported.

### Adherence to national and international regulations

All the fungal strains used in this study have been legally obtained, respecting the Convention on Biological Diversity (Rio Convention).

### Author ORCIDs

Man Mu https://orcid.org/0000-0002-3613-5308

Young-Joon Choi https://orcid.org/0000-0002-0909-4723

Thomas Brand https://orcid.org/0000-0001-6026-0806

Sebastian Ploch https://orcid.org/0000-0002-7129-4523

Hermann Voglmayr https://orcid.org/0000-0001-7666-993X

Marco Thines https://orcid.org/0000-0001-7740-6875

### Data availability

All sequence data generated in this study is available on GenBank. All other data that support the findings of this study are available in the main text and supplementary materials.

## Supplementary materials

Supplementary material 1 Phylogenetic trees inferred from single loci, contains figs S1–S7 https://doi.org/10.3897/imafungus.17.186696.suppl1 Data type pdf Explanation note **fig. S1**. Phylogenetic tree inferred from Maximum Likelihood analysis of an alignment of ITS with *Pseudoperonospora cubensis* as the outgroup. **fig. S2**. Phylogenetic tree inferred from Maximum Likelihood analysis of an alignment of LSU D1–3 with *Pseudoperonospora cubensis* as the outgroup. **fig. S3**. Phylogenetic tree inferred from Maximum Likelihood analysis of an alignment of LSU D6–8 with *Pseudoperonospora cubensis* as the outgroup. **fig. S4**. Phylogenetic tree inferred from Maximum Likelihood analysis of an alignment of *COX*1 with *Pseudoperonospora cubensis* as the outgroup. **fig. S5**. Phylogenetic tree inferred from Maximum Likelihood analysis of an alignment of *COX*2 with *Pseudoperonospora cubensis* as the outgroup. **fig. S6**. Phylogenetic tree inferred from Maximum Likelihood analysis of an alignment of *NAD1* with *Pseudoperonospora cubensis* as the outgroup. **fig. S7**. Phylogenetic tree inferred from Maximum Likelihood analysis of an alignment of *RPS10* with *Pseudoperonospora cubensis* as outgroup. https://binary.pensoft.net/file/1567633This dataset is made available under the Open Database License (http://opendatacommons.org/licenses/odbl/1.0/). The Open Database License (ODbL) is a license agreement intended to allow users to freely share, modify, and use this Dataset while maintaining this same freedom for others, provided that the original source and author(s) are credited. Man Mu, Young-Joon Choi, Thomas Brand, Sebastian Ploch, Hermann Voglmayr, Marco Thines
