# Supplementary figures and images for: A revision of Peronospora species on Veronica unravels a species-rich group of downy mildew pathogens with host shifts to economically important ornamental plants
Source: IMA Fungus. 2026 Mar 23;17:e186696. doi: 10.3897/imafungus.17.186696 (PMC13036465; doi:10.3897/imafungus.17.186696)

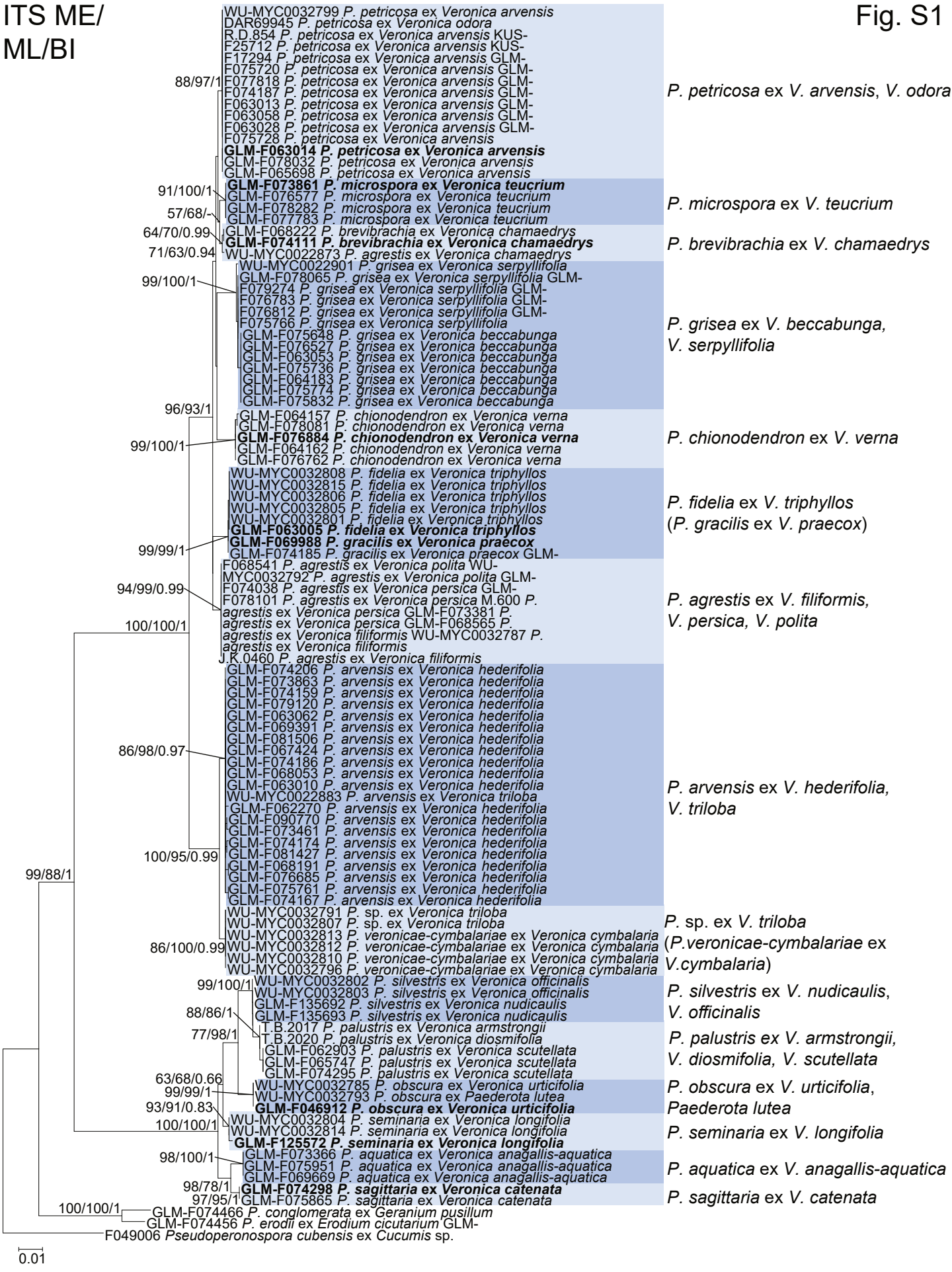

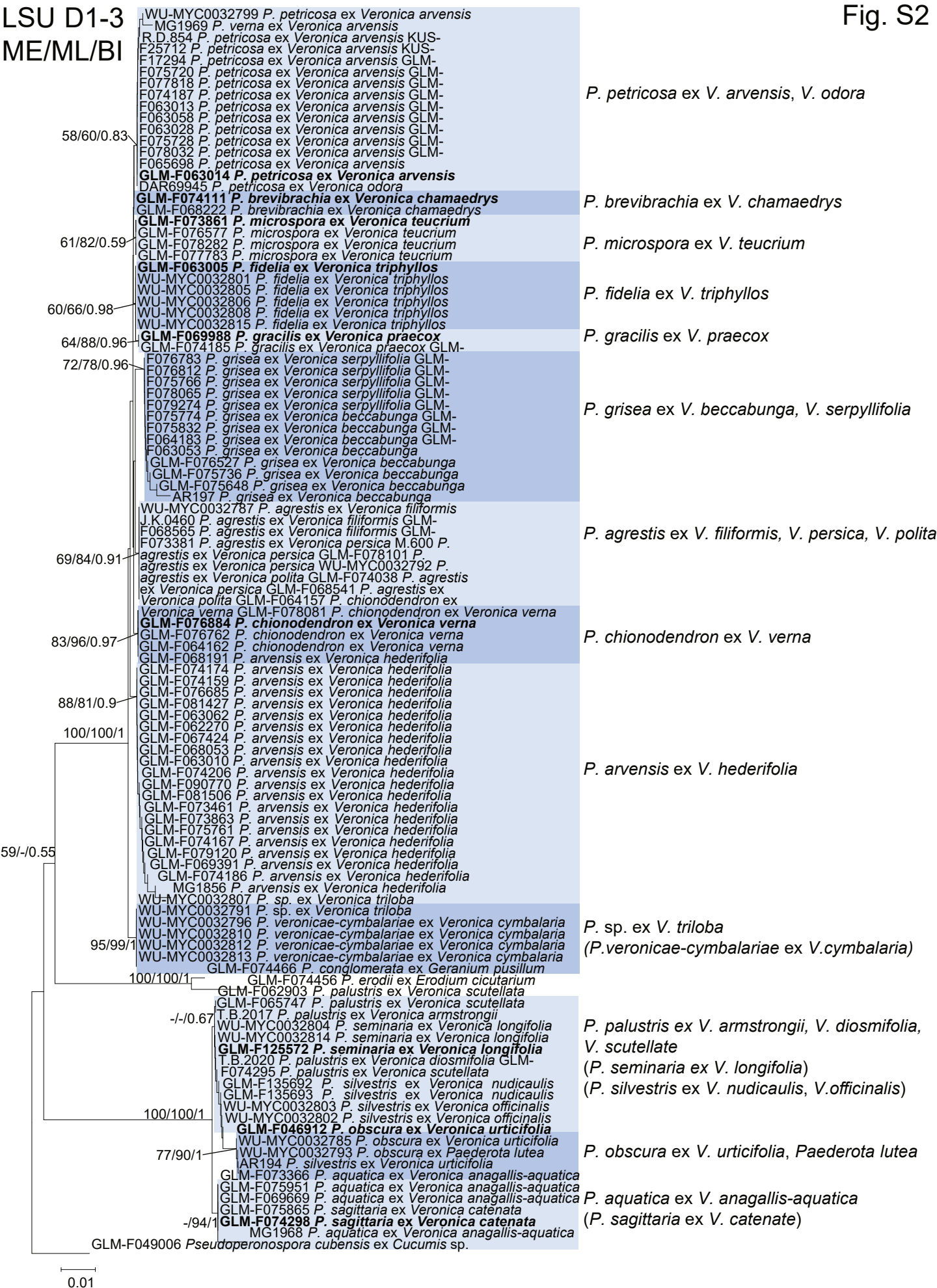

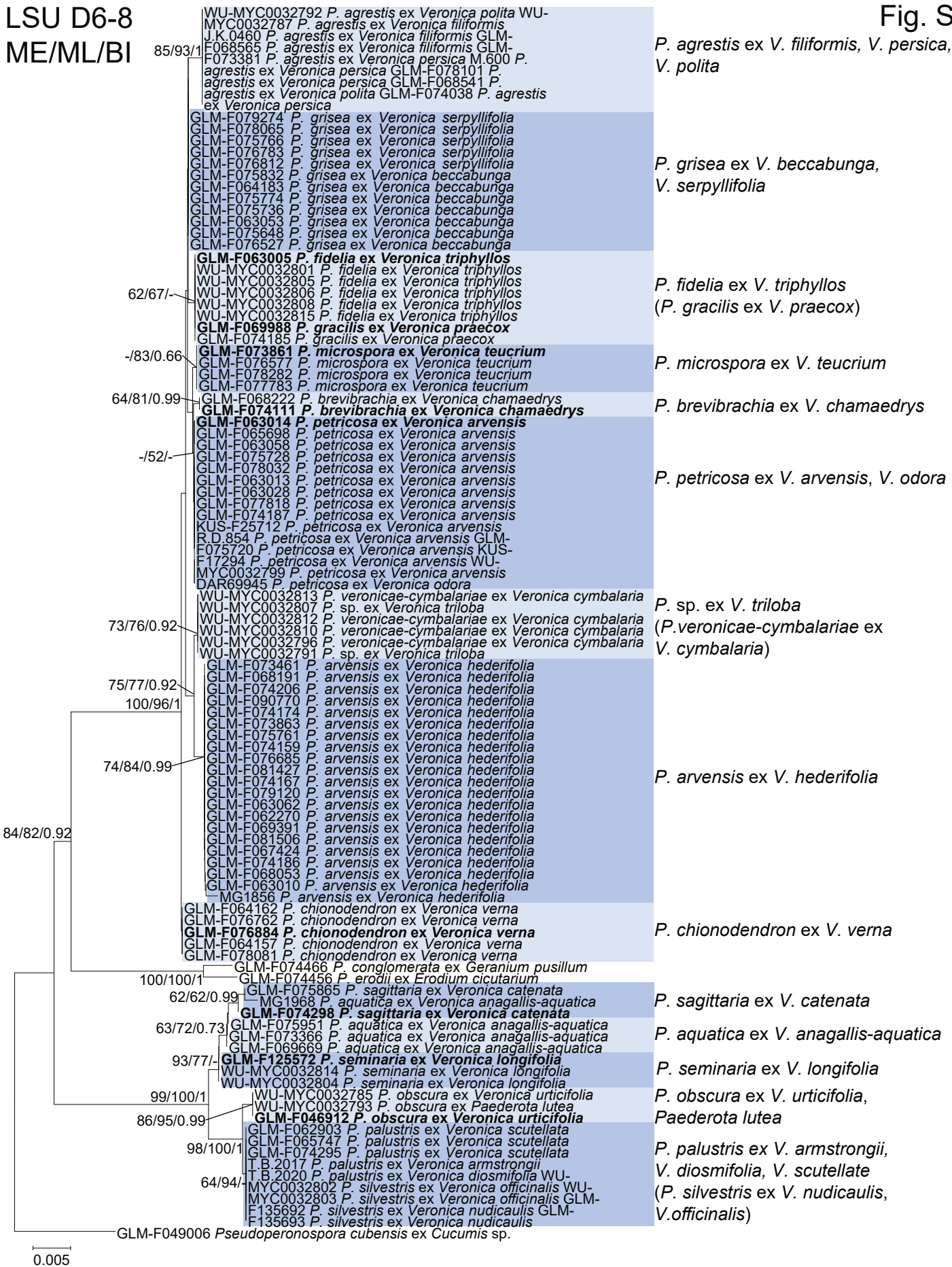

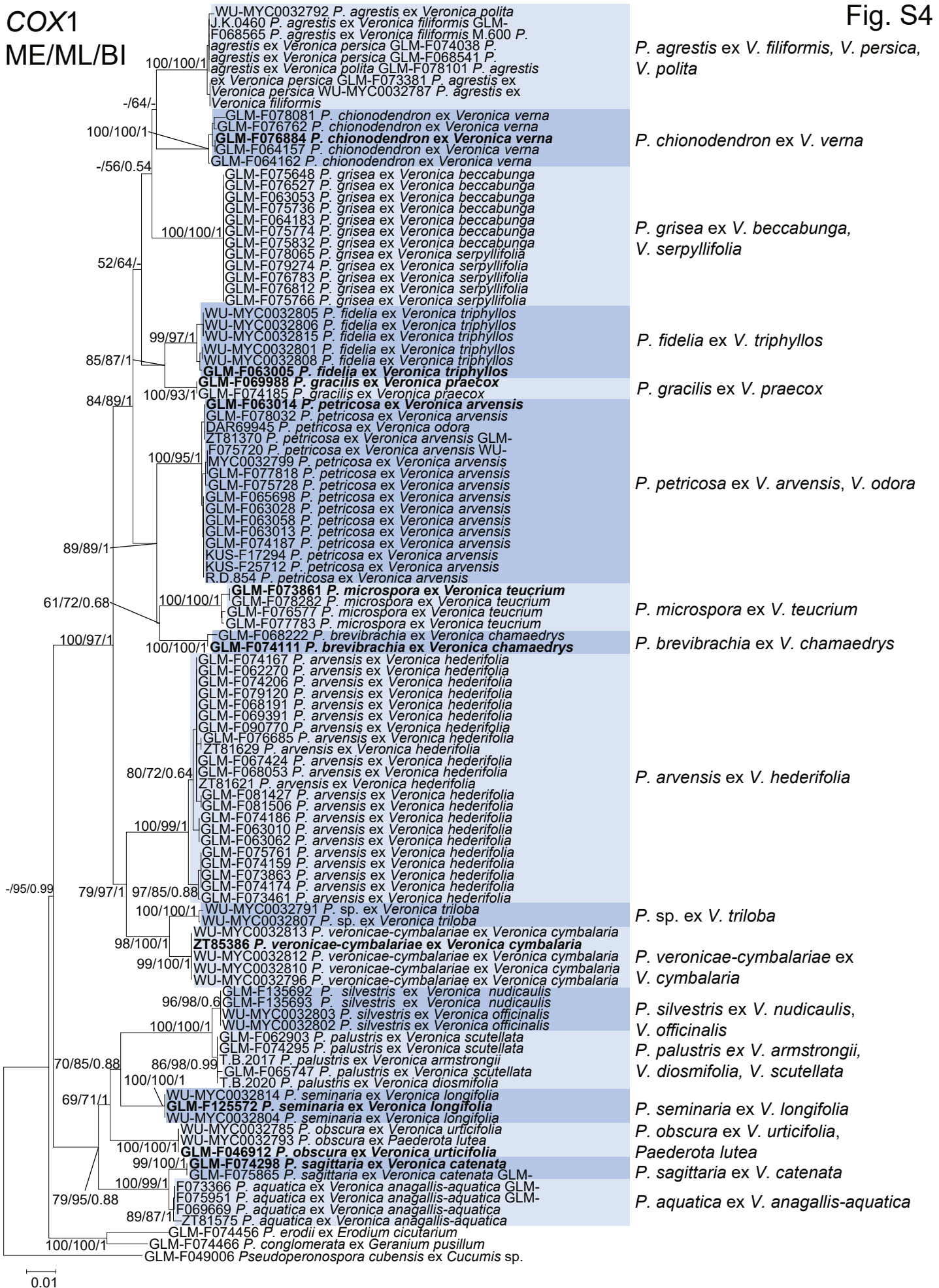

Fig. S5

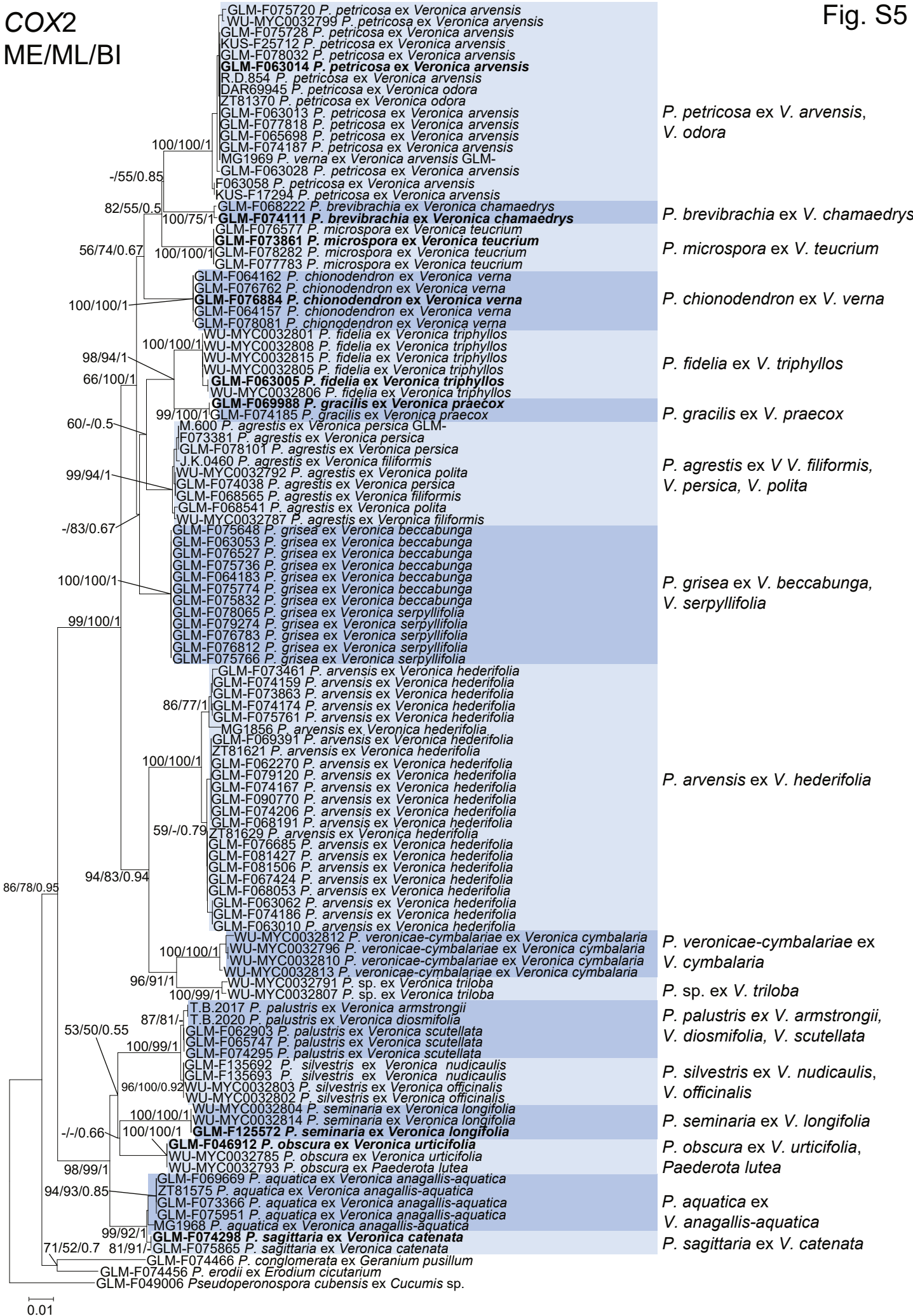

# NAD1

## ME/ML/BI

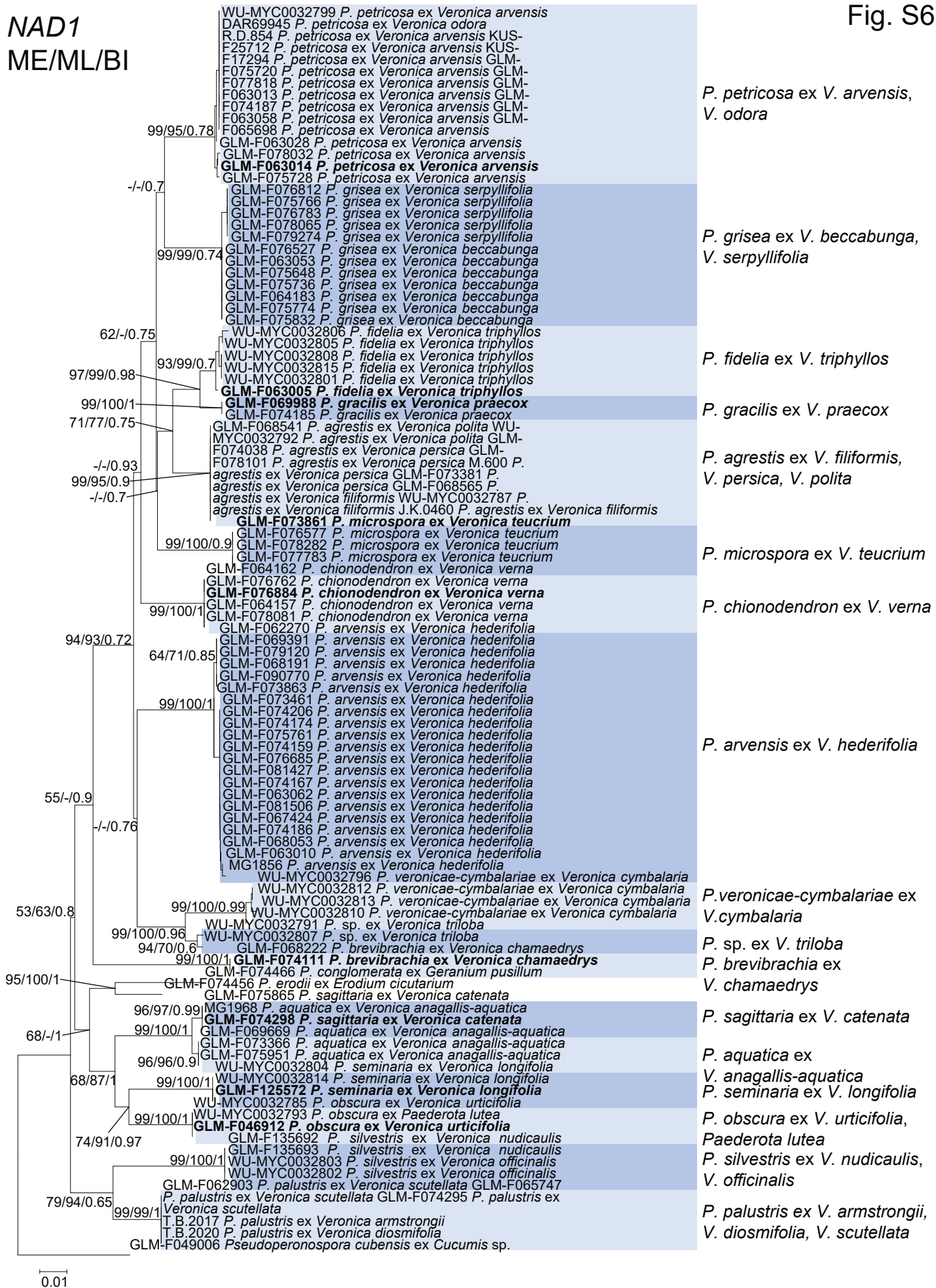

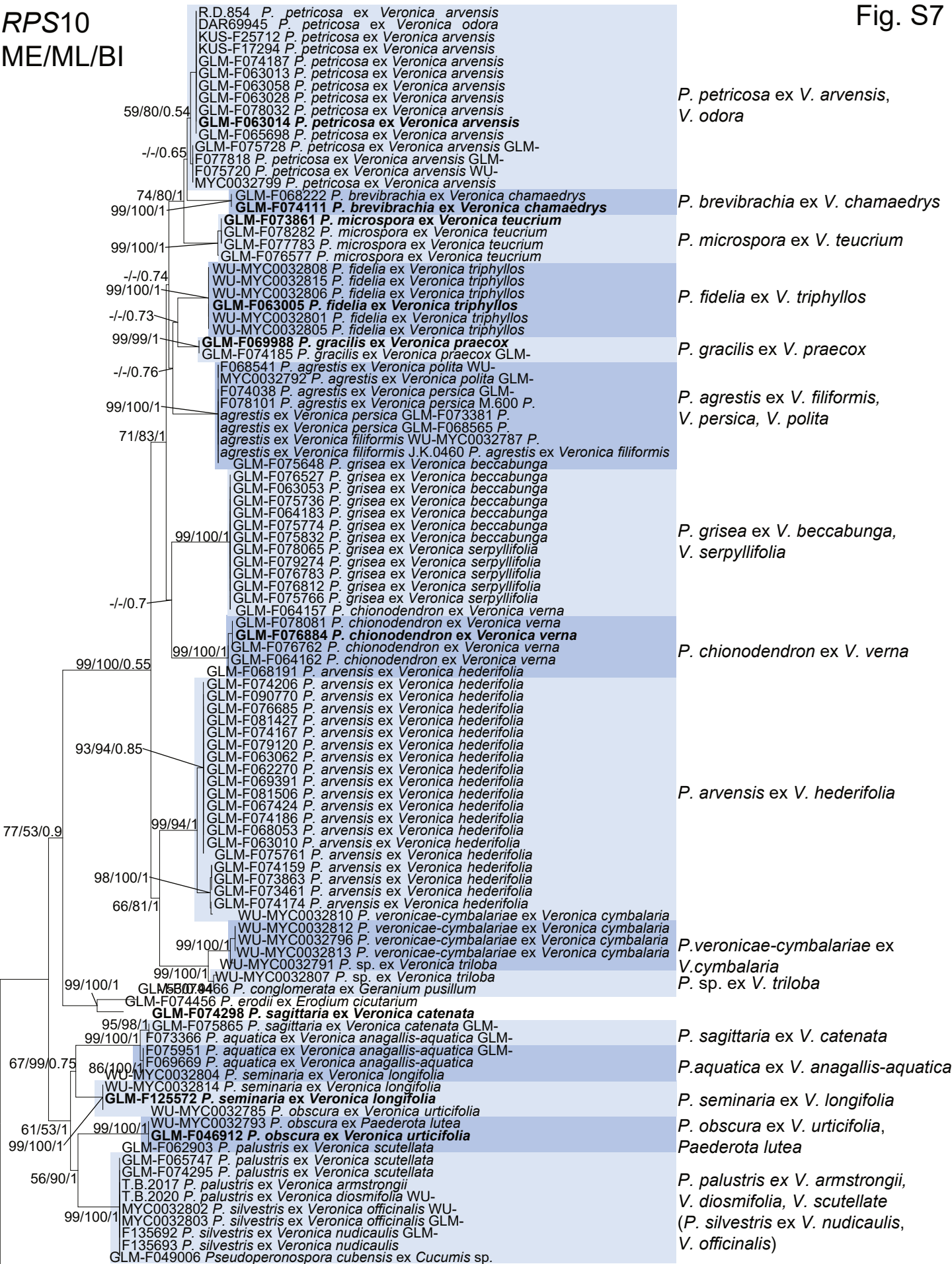

Supplement: Supplementary material 1 — Phylogenetic trees inferred from single loci, contains figs S1–S7 [file imafungus-17-e186696-s001.pdf]
